# Controlled sequential in situ self-assembly and disassembly of a fluorogenic cisplatin prodrug for cancer theranostics

**DOI:** 10.1038/s41467-023-36469-1

**Published:** 2023-02-13

**Authors:** Xidan Wen, Rui Zhang, Yuxuan Hu, Luyan Wu, He Bai, Dongfan Song, Yanfeng Wang, Ruibing An, Jianhui Weng, Shuren Zhang, Rong Wang, Ling Qiu, Jianguo Lin, Guandao Gao, Hong Liu, Zijian Guo, Deju Ye

**Affiliations:** 1 State Key Laboratory of Analytical Chemistry for Life Science, Chemistry and Biomedicine Innovation Center (ChemBIC), School of Chemistry and Chemical Engineering, Nanjing University, 163 Xianlin Road, Nanjing, 210023 China; 2 State Key Laboratory of Drug Research, Shanghai Institute of Materia Medica, Chinese Academy of Sciences, 555 Zu Chong Zhi Road, Shanghai, 201203 China; 3 State Key Laboratory of Coordination Chemistry, Chemistry and Biomedicine Innovation Center (ChemBIC), School of Chemistry and Chemical Engineering, Nanjing University, 163 Xianlin Road, Nanjing, 210023 China; 4 State Key Laboratory of Pollution Control and Resource Reuse, School of Environment, Nanjing University, 163 Xianlin Road, Nanjing, 210023 China; 5 NHC Key Laboratory of Nuclear Medicine, Jiangsu Key Laboratory of Molecular Nuclear Medicine, Jiangsu Institute of Nuclear Medicine, Wuxi, 214063 China

**Keywords:** Imaging techniques and agents, Drug delivery, Chemotherapy

## Abstract

Temporal control of delivery and release of drugs in tumors are important in improving therapeutic outcomes to patients. Here, we report a sequential stimuli-triggered in situ self-assembly and disassembly strategy to direct delivery and release of theranostic drugs in vivo. Using cisplatin as a model anticancer drug, we design a stimuli-responsive small-molecule cisplatin prodrug (P-CyPt), which undergoes extracellular alkaline phosphatase-triggered in situ self-assembly and succeeding intracellular glutathione-triggered disassembly process, allowing to enhance accumulation and elicit burst release of cisplatin in tumor cells. Compared with cisplatin, P-CyPt greatly improves antitumor efficacy while mitigates off-target toxicity in mice with subcutaneous HeLa tumors and orthotopic HepG2 liver tumors after systemic administration. Moreover, P-CyPt also produces activated near-infrared fluorescence (at 710 nm) and dual photoacoustic imaging signals (at 700 and 750 nm), permitting high sensitivity and spatial-resolution delineation of tumor foci and real-time monitoring of drug delivery and release in vivo. This strategy leverages the advantages offered by in situ self-assembly with those of intracellular disassembly, which may act as a general platform for the design of prodrugs capable of improving drug delivery for cancer theranostics.

## Abstract (web summary)

Manipulating molecular self-assembly and disassembly in vivo may permit temporal control of drug delivery and release. Here, the authors report a fluorogenic cisplatin prodrug for cancer theranostics by leveraging stimuli-triggered in situ self-assembly and intracellular disassembly processes.

## Introduction

Self-assembly and disassembly is a reverse process pervasive in biology, participating in many essential physiological processes and functions^1–3^. Learned from nature, people have utilized the self-assembly process to synthesize nano/microstructures, and the disassembly process to control drug release in biology^4–6^. Of particular interest is the stimuli-triggered in situ self-assembly process that allows on-site synthesis of high-order nanostructures in living subjects^7,8^. In this respect, small-molecule compounds with a well-defined chemical structure are administrated instead of preformed nanomaterials, which are shown to enter disease tissues and self-assemble into nanocomposites upon activation by biological targets. The small size of molecules permits facile diffusion into disease tissues, while the on-site formed nanocomposites are not cleared as easily benefited from the increased molecular size, which retards diffusion and leads to a higher local accumulation in the target tissues. This stimuli-triggered in situ self-assembly strategy has offered versatile applications that range from molecular imaging to controlled drug delivery^9–13^. Integration of this process with stimuli-triggered disassembly could permit not only enhanced drug delivery but also on-demand drug release in disease tissues, which would be promising to improve therapeutic efficacy^14–17^. However, the design of a small-molecule prodrug capable of undergoing in situ self-assembly and intracellular disassembly to direct delivery and release of drugs in vivo remains elusive. The highly dynamic and complex in vivo environment presents significant difficulties to precisely control and monitor this delicate process. Recently, we reported an alkaline phosphatase (ALP)-triggered in situ self-assembly approach that enabled to design an activatable near-infrared (NIR) fluorescence and magnetic resonance imaging (MRI) small-molecule probe for in vivo imaging of ALP activity^12^. In this paper, using cisplatin (CDDP) as a model drug, we report a sequential stimuli-triggered in situ self-assembly and disassembly system amenable to design a fluorogenic small-molecule cisplatin prodrug (P-CyPt) for cancer theranostics.

Cisplatin is one of the first-line anti-cancer drugs for the treatment of various malignant tumors in clinic. However, the therapeutic efficacy is compromised by the non-specific biodistribution and insufficient tumor uptake that cause systemic toxicities (e.g., nephrotoxicity, neurotoxicity) and drug resistance^18,19^. While myriads of cisplatin prodrugs and nanocarrier-based delivery systems have been developed to overcome the side-toxicity and drug resistance^20–24^, the in vivo therapeutic efficacy is still not satisfied, probably due to that the delivery of cytotoxic CDDP in tumor tissues is not substantially improved^25^.

Here, by leveraging membrane-bound ALP-triggered in situ self-assembly with intracellular glutathione (GSH)-triggered disassembly, we demonstrate that P-CyPt can potentiate delivery and release of CDDP in tumor cells, thus greatly improving the therapeutic efficacy as compared with CDDP or preformed Pt(IV) nanoparticles. Application of P-CyPt in vivo allows us to successfully visualize and treat orthotopic HepG2 liver tumors in living mice. Moreover, this sequential in situ self-assembly and disassembly process can be monitored in real time via complemental fluorescence (FL) and photoacoustic (PA) bimodality imaging. This strategy is important for cancer theranostics and may act as a general approach able to improve delivery and direct release of therapeutic agents in solid tumors after systemic administration.

## Results

### Design of P-CyPt

**Fig. 1:**
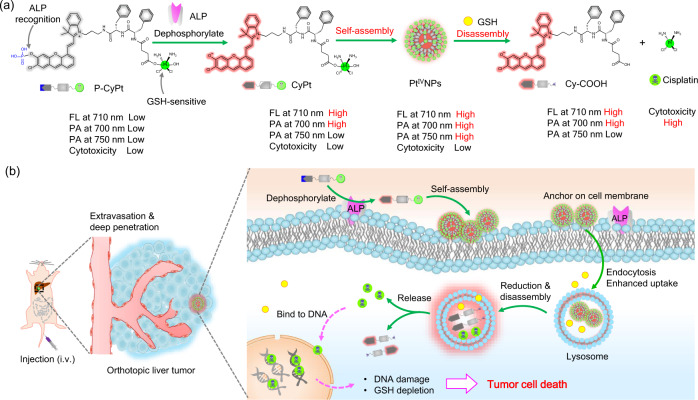
General design and proposed mechanism of P-CyPt for cancer theranostics. **a** Chemical structure of P-CyPt and proposed ALP-triggered dephosphorylation and in situ self-assembly into Pt^IV^NPs, followed by GSH-triggered disassembly to release cisplatin and Cy-COOH. The NIR FL at 710 nm and PA signal at 700 nm were both turned on; the PA signal at 750 nm was turned on during the in situ self-assembly process, which was switched off during the disassembly process. **b** Illustration of the mechanism of P-CyPt for FL/PA bimodal imaging-guided chemotherapy of orthotopic liver tumors in vivo. Following systemic administration, P-CyPt as a hydrophilic small-molecule cisplatin prodrug easily extravasates and diffuses into tumor tissues. In orthotopic liver tumors, rapid dephosphorylation of P-CyPt into fluorescent CyPt is initiated by membrane-bound ALP, which subsequently undergoes in situ self-assembly into Pt^IV^NPs, producing strong NIR FL and dual PA imaging signals (700 and 750 nm). The in situ formed Pt^IV^NPs are capable of anchoring on cell membranes where the ALP locates, thereby prolonging probe retention and enhancing endocytosis into tumor cells. Within tumor cells, the intracellular GSH rapidly reduces Pt^IV^NPs and induces disassembly, which leads to a burst release of cytotoxic cisplatin and fluorescent Cy-COOH. This process markedly raises intracellular cisplatin and concurrently depletes GSH, which effectively kills tumor cells. In normal tissues that are ALP-deficient, P-CyPt cannot be dephosphorylated, which shows low bimodal imaging signals and fast clearance, facilitating to mitigate off-target toxicity.

P-CyPt is designed to (1) offer activatable near-infrared (NIR) FL and PA signals for bimodality imaging through ALP-mediated in situ self-assembly and (2) release CDDP for on-demand therapy via GSH-triggered disassembly (Fig. 1a). P-CyPt comprises (1) a NIR merocyanine fluorophore (mCy)^26,27^ capped with an ALP-recognition phosphate group (PO_3_H)^28^, (2) a GSH-reducible CDDP prodrug (Pt(IV))^29,30^, and (3) a hydrophobic _*D*_-Phe-_*D*_-Phe (FF) dipeptide^31^, which shows low NIR FL, low PA signal and low cytotoxicity. After systemic administration, P-CyPt as a small molecule can extravasate and penetrate deeply into tumor tissues (Fig. 1b). In tumor tissues that are membrane-bound ALP positive, rapid dephosphorylation of P-CyPt by the membrane-bound ALP generates CyPt, switching on NIR FL (*λ*_em_ = 710 nm) and PA signals (*λ* = 700 nm); moreover, CyPt with increased hydrophobicity is susceptible to self-assembly into Pt(IV) nanoparticles (Pt^IV^NPs), which turns on another PA signal (*λ* = 750 nm) resulting from the aggregation-caused bathochromic shift. The on-site formed Pt^IV^NPs can anchor on the cell membranes, which attenuate diffusion, prolong retention, and enhance translocation into cells^32,33^. Within tumor cells, the abundant endogenous GSH can reduce Pt(IV) into Pt(II), producing negatively charged Cy-COOH that promotes NPs disassembly and accelerates release of CDDP intracellularly. This process dramatically raises intracellular CDDP’s concentration while depletes GSH’s level to effectively kill tumor cells^34^. Moreover, the disassembly of Pt^IV^NPs into Cy-COOH further increases NIR FL (called as “ON-ON”) as the aggregation-caused quenching (ACQ) effect is eliminated; however, the PA signal at 750 nm is turned off but the PA signal at 700 nm is kept at “ON” state. The sequential switches in NIR FL and PA bimodal imaging signals can allow P-CyPt to reliably monitor the ALP-triggered in situ self-assembly and GSH-driven disassembly process in vivo, facilitating to guide precise chemotherapy of tumors. By contrast, in normal tissues that are ALP deficient, P-CyPt cannot be dephosphorylated and shows poor cellular uptake, resulting in rapid clearance from normal tissues, beneficial to lower systemic toxicities.

### ALP-triggered in situ self-assembly and GSH-driven disassembly in vitro

**Fig. 2:**
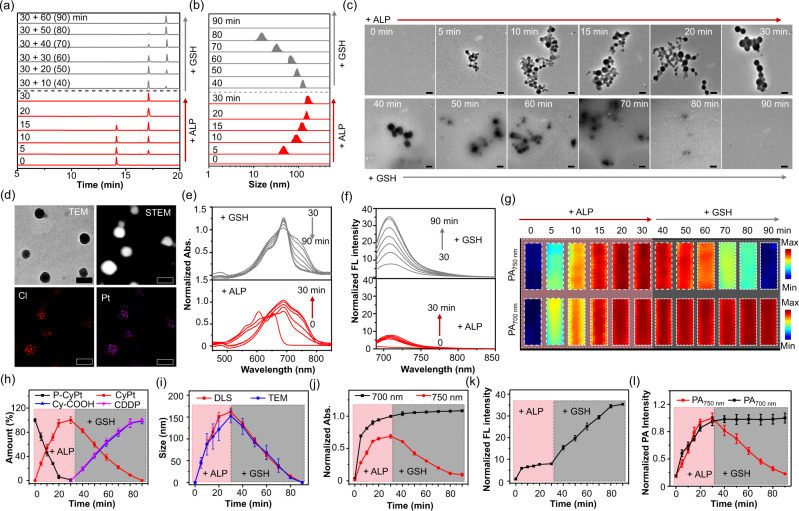
Characterization of ALP-triggered in situ self-assembly and GSH-driven disassembly of P-CyPt in vitro. **a** High-performance liquid chromatography (HPLC) trace, **b** dynamic light scattering (DLS) analysis, **c** transmission electron microscopic (TEM) analysis of P-CyPt (10 μM) incubated with alkaline phosphatase (ALP,100 U/L, 37 °C) in Tris buffer for 0–30 min, followed by addition with glutathione (GSH,10 mM) and incubation for another 0–60 min. Scale bars: 200 nm. **d** TEM image, scanning transmission electron microscopic (STEM) image, and elemental mapping of Pt^IV^NPs formed by incubating P-CyPt (10 μM) with ALP (100 U/L, 37 °C) for 30 min. Color red and magenta indicate elements of chlorine (Cl) and platinum (Pt), respectively. Scale bars: 200 nm. **e** Ultraviolet–visible (UV-vis) absorption (Abs.), **f** fluorescence (FL) spectra and **g** photoacoustic (PA) images (at 700 and 750 nm) of P-CyPt (10 μM) incubated with ALP (100 U/L, 37 °C) in Tris buffer for 0–30 min, followed by addition with GSH (10 mM) and incubation for another 0–60 min. **h** Quantification of P-CyPt (black), CyPt (red), Cy-COOH (blue) and cisplatin (CDDP, magenta) in the solution of P-CyPt (10 μM) following incubation with ALP (100 U/L, 37 °C) for 0–30 min, and then with GSH (10 mM) for another 0–60 min. **i** Plots of the mean sizes analyzed by DLS (red) and TEM (blue) in the solution of P-CyPt (10 μM) following incubation with ALP (100 U/L, 37 °C) for 0–30 min, and then with GSH (10 mM) for another 0–60 min. **j** Normalized Abs. (at 700&750 nm), **k** normalized FL intensity and **l** normalized PA intensities (at 700 and 750 nm) of P-CyPt (10 μM) following incubation with ALP (100 U/L, 37 °C) for 0–30 min, and then with GSH (10 mM) for another 0–60 min. All the data are mean ± standard deviation (s.d.) (*n* = 3 independent experiments for **h**–**l**). One representative experiment out of three (**c**) or two (**d**) is shown. Source data are provided as a Source Data file.

P-CyPt was synthesized according to the procedure illustrated in Supplementary Methods. We first monitored the ALP-triggered dephosphorylation and self-assembly of P-CyPt into Pt^IV^NPs in solution. To ensure efficient enzymatic reaction with P-CyPt in Tris buffer (pH 8.0), 100 U/L ALP was chosen for in vitro studies (Supplementary Fig. 1). On incubation with ALP, P-CyPt was completely converted into CyPt after 30 min (Fig. 2a, h, and Supplementary Fig. 2), and the Log*P* value distinctly increases from 0.68 ± 0.03 to 2.50 ± 0.03. Molecular simulations predicted that P-CyPt as a hydrophilic probe was basically present at a disperse state in aqueous solutions, while CyPt with an increased hydrophobicity was prone to closely pack into aggregates benefited from strong intermolecular interactions (e.g., *π*-*π* stacking, van der Waals interaction and hydrogen bonding) (Supplementary Figs. 3 and 4). Dynamic light scattering (DLS) and transmission electron microscopic (TEM) analysis showed bare NPs for P-CyPt, which progressively assembled into spherical Pt^IV^NPs, with a mean size of ~160 nm after 30 min (Fig. 2b–d). The UV-vis-NIR absorption of P-CyPt bathochromically shifted from 605 and 652 nm to 690 nm plus a shoulder peak at 750 nm (Fig. 2e, j); the initially quenched NIR FL at 710 nm was switched on by ~8-fold (Fig. 2f, k), and the PA signals at 700 and 750 nm both increased by more than sixfold after 30 min (Fig. 2g, l). Importantly, P-CyPt displays fast enzymatic kinetics, high sensitivity, and specificity toward ALP (Supplementary Figs. 5–7).

We next investigated the GSH-triggered reduction and disassembly of Pt^IV^NPs to release CDDP. Pt^IV^NPs hold a good stability in aqueous solutions, attributable to the low critical micellar concentration (CMC ≈ 0.3 μM) of CyPt (Supplementary Fig. 8). On addition of 5 or 10 mM GSH that can mimic different intracellular centration of GSH in tumor cells^35,36^, CyPt in Pt^IV^NPs could be completely reduced into Cy-COOH after 60 min (Fig. 2a, h, Supplementary Figs. 9 and 10). ^195^Pt nuclear magnetic resonance (^195^Pt-NMR) spectrometric and X-ray photoelectron spectroscopic (XPS) analysis confirmed the reduction of Pt(IV) to Pt(II) by GSH (Supplementary Fig. 11). We then quantified the amount of released Pt(II) using inductively coupled plasma optical emission spectrometry (ICP-OES), which showed nearly complete release of CDDP after 60 min (Fig. 2h). DLS analysis showed that the size of Pt^IV^NPs progressively declined, with bare NPs observed after 60 min (Fig. 2b, i), aligning well with TEM images (Fig. 2c, i). Accompanying with the disassembly, the NIR absorption at 700 nm slightly increased while the shoulder peak at 750 nm gradually decreased to the baseline (Fig. 2e, j). Accordingly, the NIR FL at 710 nm further increased by ~4.5-fold (Fig. 2f, k); concurrently, the PA signal at 750 nm progressively decreased by approximately sixfold, but the PA signal at 700 nm remained strong (Fig. 2d, e). These findings demonstrate that GSH efficiently reduces Pt^IV^NPs and triggers disassembly to release Cy-COOH and Pt(II) drug, resulting in (1) a further enhancement in NIR FL at 710 nm and (2) a decline in PA signal at 750 nm (not at 700 nm). The increased 710 nm FL but decreased 750 nm PA signal could be used to complementally monitor the GSH-triggered disassembly process that synchronized CDDP release, and the unchanged 700 nm PA signal could act as an internal standard for calibration.

### P-CyPt empowers FL and PA bimodality imaging of ALP-positive tumor cells

**Fig. 3:**
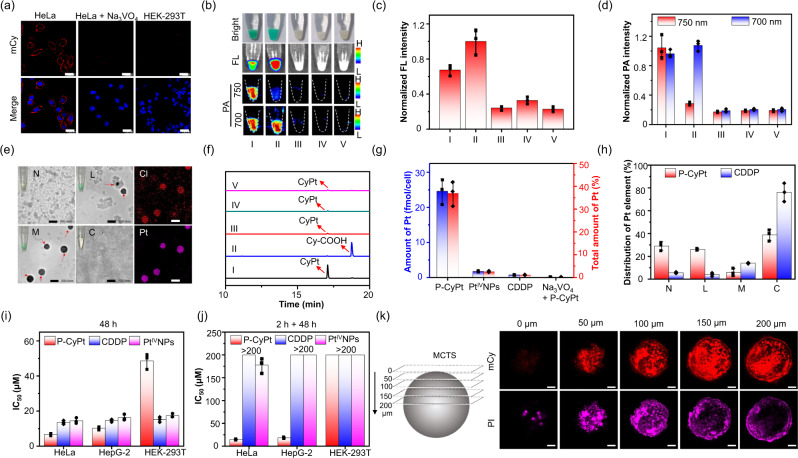
Evaluation of P-CyPt in cells. **a** FL imaging of HeLa cells, Na_3_VO_4_-pretreated HeLa cells, or HEK-293T cells incubated with P-CyPt. Scale bars: 25 μm. **b** Photographs (Bright), FL (*λ*_ex/em_ = 670/(750 ± 50) nm) and PA images (700 and 750 nm), **c** normalized FL, and **d** PA intensities (700 and 750 nm) of indicated cell pellets. I: HeLa cells incubated with P-CyPt (10 μM, 30 min); II: HeLa cells incubated with P-CyPt (10 μM, 30 min), then incubated in blank mediums for 3 h; III: HeLa cells preincubated with Na_3_VO_4_ (10 mM, 20 min), then incubated with P-CyPt (10 μM, 30 min); IV: HeLa cells incubated with preformed Pt^IV^NPs (10 μM, 30 min); V: HEK-293T cells incubated with P-CyPt (10 μM, 30 min). **e** TEM images and photographs (inset) of HeLa cell fractions after incubating with P-CyPt (10 μM, 1 h). N: nucleus; L: lysosomes and mitochondria; M: membrane; C: cytosol. Pseudo red and magenta images are elemental mapping of Cl and Pt in fraction M. Scale bars: 200 nm. **f** HPLC analysis of cell lysates with conditions in **b**. **g** ICP-OES determines Pt uptake (fmol/cell, red) and percentage of Pt drugs (%, blue) in HeLa cells. **h** Distribution of Pt element (% of total intracellular Pt) in different subcellular organelles of HeLa cells after incubating with P-CyPt and CDDP (10 μM). (i, j) The IC_50_ of P-CyPt, Pt^IV^NPs, and CDDP toward HeLa, HepG2, and HEK-293T cells. **i** Cells were incubated with Pt drugs for 48 h; **j** cells were first incubated with Pt drugs for 2 h, then incubated in blank mediums for 48 h. **k** Evaluation of the penetration and toxicity of P-CyPt toward HeLa MCTS. Scale bars: 100 μm. Data denote mean ± s.d. (*n* = 3 independent cell pellets for **c** and **d**; *n* = 3 independent experiments for **g**–**j**). One representative experiment out of two (**a**, **e**, **k**) is shown. Source data are provided as a Source Data file.

We first employed the immunofluorescence staining assay to validate the overexpression of ALP on the membranes of HeLa cells (Supplementary Fig. 12)^37^. On incubation with P-CyPt, ALP-positive HeLa cells displayed distinct mCy’s FL around the cell membranes, which intensified with the concentration and incubation time of P-CyPt (Supplementary Figs. 13 and 14). When extending the incubation time to over 40 min, strong NIR FL punctate also appeared in the lysosomes through clathrin-dependent endocytosis (Supplementary Figs. 15 and 16). By contrast, the mCy’s FL was negligibly observed neither in HeLa cells pretreated with Na_3_VO_4_ (an inhibitor of ALP^38^) nor in ALP-deficient HEK-293T cells (Fig. 3a). The incubation of HeLa cells with preformed fluorescent Pt^IV^NPs produced strong mCy’s FL in the culture medium only, contrary to that of P-CyPt (Supplementary Fig. 17). We then acquired the macrofluorescence and PA images of cell pellets and culture mediums. Consistent with the epifluorescence images, bright NIR FL and PA images (at 700 and 750 nm) appeared in the pellets of HeLa cells incubated with P-CyPt (Group I), which could be largely prohibited by Na_3_VO_4_ (Group III). In contrast, weak NIR FL and PA signals appeared either in HeLa cells incubated with preformed Pt^IV^NPs (Group IV) or in HEK-293T cells incubated with P-CyPt (Group V). These results were recapitulated with the appearance of green color in P-CyPt-treated HeLa cell pellets (Fig. 3b). Meanwhile, only the medium of HeLa cells treated with preformed Pt^IV^NPs (Group IV) displayed strong NIR FL and PA signals, indicating that most of the preformed Pt^IV^NPs were remaining disperse in the culture medium (Supplementary Fig. 18). Note that the appearance of strong 750 nm PA signal in P-CyPt-treated HeLa cell pellets could imply the formation of Pt^IV^NPs (Group I). Then, the P-CyPt-treated cells continued incubation in refreshed blank mediums for another 3 h to allow intracellular translocation of in situ formed Pt^IV^NPs (Supplementary Fig. 19). It was found that the NIR FL in the pellets increased by ~1.5-fold, but the PA signal at 750 nm (not at 700 nm) decreased by ~3.5-fold (Fig. 3c, d, Group II). These results imply the efficient disassembly of Pt^IV^NPs after entering HeLa cells.

We next employed TEM to image the in situ formed Pt^IV^NPs on HeLa cells incubated with P-CyPt (10 μM, 1 h). TEM images of the separated HeLa cell fractions^32,38^, including membranes (M), lysosomes and mitochondria (L), nuclei (N), and cytosol (C) showed the presence of NPs in fractions M and L (Fig. 3e), with size and morphology according well with that of Pt^IV^NPs (Fig. 2d); element mapping analysis revealed the existence of Pt and Cl elements in the observed NPs (Fig. 3e). In contrast, no Pt^IV^NPs appeared neither in fractions N and C of P-CyPt-treated HeLa cells nor in all the fractions of blank HeLa cells (Supplementary Fig. 20). Then, HPLC analysis of cell lysates and culture mediums showed that CyPt was predominantly present in the lysates of HeLa cells incubated with P-CyPt (10 μM, 30 min), while negligible CyPt was observed in the lysates of HEK-293T cells or Na_3_VO_4_-pretreated HeLa cells (Fig. 3f). On incubation of HeLa cells with preformed Pt^IV^NPs, negligible CyPt appeared in the lysates and most CyPt remained in the culture mediums (Supplementary Fig. 18d). Moreover, when P-CyPt-treated HeLa cells were kept incubated in refreshed mediums for additional 3 h, nearly all formed CyPt was reduced into Cy-COOH (Fig. 3f, Group II), correlating to that of increased NIR FL but reciprocally reduced PA signal at 750 nm in HeLa cells (Fig. 3b). These findings validate that (1) the uptake of P-CyPt by HeLa cells was more efficient than preformed Pt^IV^NPs via the membrane-bound ALP-triggered dephosphorylation and in situ self-assembly processes; (2) most intracellular Pt^IV^NPs could be reduced by endogenous GSH that facilitates disassembly and intracellular release of CDDP.

### P-CyPt augments cytotoxicity against tumor cells

We first quantified the amount of Pt(II) in HeLa cells by ICP-OES, which showed a large amount of Pt (24.60 ± 3.5 fmol/cell) in P-CyPt-treated HeLa cells, equal to 36.9 ± 5.3% of P-CyPt added (Fig. 3g and Supplementary Table 1). In contrast, a much lower amount of Pt was present in HeLa cells incubated with CDDP (0.73 ± 0.09 fmol/cell), Pt^IV^NPs (1.73 ± 0.2 fmol/cell) or Na_3_VO_4_ plus P-CyPt (0.07 ± 0.01 fmol/cell). Notably, more intracellular GSH (~37.3%) could be depleted in HeLa cells treated with P-CyPt than that with CDDP (~8.3%) (Supplementary Table 2), which could synergize cytotoxicity via disrupting the intracellular redox homeostasis and breaking double-strand DNA molecules (Supplementary Fig. 21). We then analyzed the subcellular distribution of Pt element in HeLa cells. Figure 3h showed that the intracellular Pt was majorly distributed in the cytosol of CDDP-treated HeLa cells (76.22%), approximately twofold higher than that of P-CyPt-treated cells (38.82%). However, ~29.05% of intracellular Pt was distributed in the nucleus of P-CyPt-treated HeLa cells, which was significantly fivefold higher than that of CDDP-treated HeLa cells (~5.65%). Moreover, the distribution of Pt drug in the mitochondria and lysosomes was also much higher in HeLa cells treated with P-CyPt (~26.11%) than that with CDDP (~4.11%) (Supplementary Table 3). These results reveal that the subcellular distribution of Pt drug in HeLa cells was dramatically different between P-CyPt and CDDP, which could be presumably owing to the different internalization pathway and uptake of different amount of Pt(II) drugs between them (Supplementary Fig. 22). The enhanced distribution in the nucleus and mitochondria, two major organelles for CDDP, could have benefits to improve cytotoxicity against HeLa cells^39–41^.

We next evaluated the cytotoxicity of P-CyPt against HeLa, HepG2, and HEK-293T cells, respectively (Supplementary Table 4). On incubation of P-CyPt for 48 h, the IC_50_ values toward HeLa (6.76 ± 0.68 μM) and HepG2 tumor cells (10.27 ± 0.87 μM) were smaller than CDDP (14.47 ± 1.60 μM for HeLa and 16.30 ± 1.69 μM for HepG2) or Pt^IV^NPs (13.52 ± 1.02 μM for HeLa and 14.64 ± 0.74 μM for HepG2), but the IC_50_ value of P-CyPt toward HEK-293T cells (48.60 ± 4.24 μM) was significantly higher than CDDP (17.41 ± 1.05 μM) or Pt^IV^NPs (15.19 ± 1.44 μM) (Fig. 3i and Supplementary Fig. 23). To avoid extracellular reduction of P-CyPt or Pt^IV^NPs that may release CDDP to cause cytotoxicity toward either ALP-positive or negative cells, we turned to incubate cells with these three Pt-drugs for only 2 h instead of 48 h, and then continued incubation in refreshed mediums for another 48 h (Fig. 3j and Supplementary Fig. 24). The IC_50_ values of CDDP or preformed Pt^IV^NPs against HeLa, HepG2 and HEK-293T cells all increased to >150 μM; but the IC_50_ values of P-CyPt toward HeLa and HepG2 cells remained at 14.56 ± 1.24 and 18.12 ± 1.78 μM, respectively, significantly smaller than that toward HEK-293T cells. The much lower IC_50_ values of P-CyPt than CDDP or preformed Pt^IV^NPs toward HeLa cells matched that of a significantly higher uptake of Pt(II) drug in P-CyPt**-**treated HeLa cells than that in CDDP- or preformed Pt^IV^NPs-treated HeLa cells (Fig. 3g and Supplementary Table 1). The subsequent flow cytometry analysis revealed a significantly larger apoptosis population in HeLa cells incubated with P-CyPt (~64.5%) relative to that with CDDP (~5.20%) or Pt^IV^NPs (7.33%) (Supplementary Fig. 25), supporting that P-CyPt was efficient in improving cytotoxicity against tumor cells via the successive ALP-mediated in situ self-assembly and GSH-driven disassembly process.

To demonstrate the ability to penetrate and kill deep-seated tumor cells, we further evaluated the cytotoxicity of P-CyPt and Pt^IV^NPs against HeLa cells in multicellular tumor spheroids (MCTS) using propidium iodide (PI) staining. Figure 3k showed that P-CyPt as a small molecule could deeply penetrate MCTS, producing bright NIR FL distributed throughout the MCTS (at a depth of ~200 μm). PI staining revealed a large population of cell death in the MCTS, which coincided with the NIR FL. In contrast, Pt^IV^NPs with a large size (~160 nm) showed a much shallower penetration depth in comparison to P-CyPt, and could only kill the superficially located cells (at a depth of only ~50 μm, Supplementary Fig. 26). These results validate the superior penetrability of P-CyPt over pre-formed Pt^IV^NPs, which was more efficient to kill deep-seated tumor cells.

### FL and PA bimodality imaging-guided therapy of s.c. tumors

**Fig. 4:**
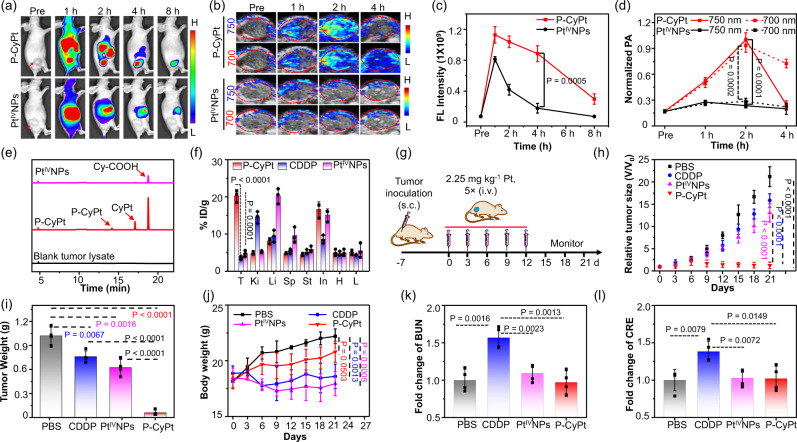
NIR FL/PA bimodal imaging and therapy of s.c. HeLa tumors in living mice with P-CyPt. **a** Longitudinal FL imaging, **b** dual PA imaging, **c** quantified FL intensity and **d** normalized dual PA intensities (at 700 and 750 nm) of HeLa tumors receiving intravenous (i.v.) injection of P-CyPt or Pt^IV^NPs (100 μM, 200 μL) at 0, 1, 2, 4, and 8 h. **e** HPLC traces (660 nm detection) of the lysates of HeLa tumors resected from mice at 4 h post i.v. injection of PBS (black), P-CyPt (red, 100 μM, 200 μL) or Pt^IV^NPs (magenta, 100 μM, 200 μL). **f** ICP-OES analysis of Pt element shows the biodistribution (% ID/g) of P-CyPt (red), CDDP (blue) and Pt^IV^NPs (magenta) in HeLa tumors and main organs (T: tumor, Ki: kidneys, Li: liver including gallbladder, Sp: spleen, St: stomach, In: intestines, H: heart, Lu: lung) of mice at 4 h post i.v. injection of each probe (100 μM, 200 μL). **g** Schematic illustration of the procedure to perform chemotherapy of s.c. HeLa tumor in living mice. **h** Changes in tumor sizes (v/v_0_) of mice following treatment with PBS, CDDP, P-CyPt or Pt^IV^NPs. **i** Average tumor weights of different groups at the end of treatment (21 day). **j** Changes in average body weight of mice following indicated treatment. **k** Comparison of the levels of blood urea nitrogen (BUN) and **l** creatinine (CRE) in the blood of mice at end of indicated treatment (21 day). Data denote mean ± s.d. (*n* = 3 independent animals for **c**, **d** and **f**; *n* = 4 independent animals for **h**–**l**). Statistical differences in **c**, **d**, **f** and **h**–**l** were analyzed by Student’s two-sided *t*-test. Source data are provided as a Source Data file.

After intravenous (i.v.) injection of P-CyPt into subcutaneous (s.c.) HeLa tumor-bearing mice, tumors displayed strong FL at 1 h, which was maintained for over 4 h (Fig. 4a). In contrast, though preformed fluorescent Pt^IV^NPs also produced bright tumor FL at 1 h, which declined quickly thereafter; the FL intensity was significantly ~4.9-fold lower than that in P-CyPt-treated tumors at 4 h (Fig. 4c). PA images showed that the tumor PA intensities at 700 and 750 nm both reached the maximum at 2 h post-injection of P-CyPt, significantly ∼3.4-fold and ∼4.3-fold higher than those injected with Pt^IV^NPs (Fig. 4b, d). Note that the 750 nm PA signals in P-CyPt-treated tumors nearly decreased to the baseline after 4 h, but the NIR FL and 700 nm PA signals remained strong. These imaging results imply that P-CyPt was majorly converted into Pt^IV^NPs in HeLa tumor tissues at 2 h, which then mostly disassembled within tumors after 4 h. Ex vivo FL imaging revealed that P-CyPt-treated tumors hold significantly brighter FL than other main organs at 4 h. The FL intensity in P-CyPt-treated tumor was ~3.2-fold higher than that of Pt^IV^NPs-treated tumors (Supplementary Fig. 27), matching that of FL imaging of resected tumor tissue slices (Supplementary Fig. 28). Furthermore, we demonstrated that P-CyPt was also amenable to detect s.c. HeLa tumors with a size of only 3.8 ± 0.8 mm^3^ in living mice via sensitive NIR FL imaging, suggesting the high potential of P-CyPt to visualize the tumors in vivo in the early stages (Supplementary Fig. 29). HPLC analysis of tumor lysates showed that most P-CyPt was converted to CyPt and Cy-COOH in HeLa tumors at 4 h; the amount of Cy-COOH was significantly ~5-fold and ~23-fold higher than that of CyPt and P-CyPt, respectively (Fig. 4e). ICP-OES analysis of Pt revealed that the ID% g^−1^ in P-CyPt-treated tumors reached ∼20.3% at 4 h, significantly higher than that in Pt^IV^NPs-treated (∼4.7%) or CDDP-treated tumors (~3.6%) (Fig. 4f). Notably, P-CyPt-treated tumors hold the highest ID% g^−1^ among all the resected organs, different to that in Pt^IV^NPs-treated tumor (in liver) or CDDP-treated tumors (in kidneys).

We then evaluated the antitumor activity against s.c. HeLa tumors in mice receiving i.v. injection of CDDP, Pt^IV^NPs or P-CyPt (Fig. 4g). The tumor growth was significantly prohibited in mice treated with P-CyPt than that treated with PBS, CDDP or Pt^IV^NPs (Supplementary Fig. 30). On day 21, no apparent tumor growth was observed in P-CyPt-treated mice, however, the tumor volume significantly grew ~21.2-, ~15.8- and 13.0-fold in PBS-, CDDP- and Pt^IV^NPs-treated mice, respectively (Fig. 4h). Accordingly, the average weight of P-CyPt-treated tumors was significantly smaller than the other three groups (Fig. 4i), verifying from the photographs of dissected tumors and immunofluorescence staining of tumor tissue slices (Supplementary Figs. 31 and 32). During the treatment, the body weights were similar between P-CyPt- and PBS-treated mice (Fig. 4j). However, the body weights obviously decreased in CDDP- or Pt^IV^NPs-treated mice (Fig. 4j). The subsequent measurement of renal injury-related biomarkers, including blood urea nitrogen (BUN) and creatinine (CRE), showed that the BUN and CRE levels in CDDP-treated mice significantly increased by ~1.6- and ~1.4-fold, which were not obviously upregulated in P-CyPt-treated mice (Fig. 4k, l). Hematoxylin-eosin (H&E) staining of major organs showed no apparent cell death occurred in major organs of P-CyPt-treated mice, while hepatotoxicity and nephrotoxicity appeared in Pt^IV^NPs- and CDDP-treated mice, respectively (Supplementary Fig. 33). These findings demonstrate that (1) Pt^IV^NPs and CDDP could cause obvious side toxicity; (2) P-CyPt was efficient in reducing low side toxicity to mice, but eliciting strong therapeutic efficacy against s.c HeLa tumors.

### P-CyPt imaging and chemotherapy in orthotopic liver tumors

**Fig. 5:**
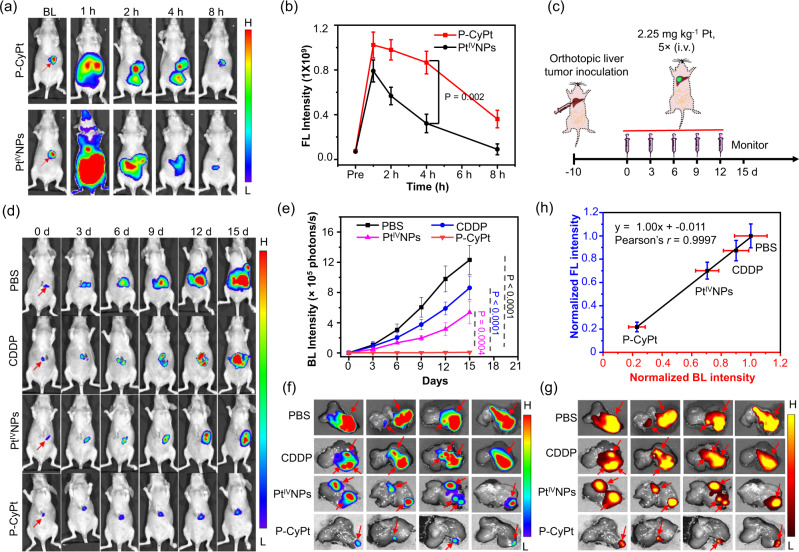
NIR FL imaging and treatment of orthotopic HepG2/Luc liver tumors in mice with P-CyPt. **a** Representative bioluminescence (BL) and FL images, and **b** quantified FL intensity of luciferase-transfected HepG2(HepG2/Luc) liver tumors in living mice receiving i.v. injection of P-CyPt or Pt^IV^NPs (100 μM, 200 μL) at 0, 1, 2, 4, and 8 h. **c** Schematic illustration of the procedure to perform chemotherapy of orthotopic HepG2/Luc liver tumors in mice. **d** Representative BL images and **e** BL intensity of liver at 0, 3, 6, 9, 12, and 15 d following indicated treatments. **f** Ex vivo BL and **g** NIR FL images of each resected liver at the end of indicated treatment (15 days). Red arrows indicate the tumor nodules in the liver tissues. **h** Plot of the normalized tumor FL intensity versus the normalized BL intensity in logarithm reveals a strong correlation (Person’s *r* = 0.9997) between them. Data denote mean ± s.d. (*n* = 3 independent animals for **b**; *n* = 4 independent animals for **g** and **h**). Statistical differences in **b**, **g** and **h** were analyzed by Student’s two-sided *t*-test. Source data are provided as a Source Data file.

We further applied P-CyPt for imaging-guided treatment of orthotopic HepG2/Luc liver tumors. Strong NIR fluorescence appeared in the liver of mice implanted with a HepG2/Luc tumor at 1 h post-injection of P-CyPt, which was maintained for over 4 h and matched well with the bioluminescence (BL) imaging region (Fig. 5a). By contrast, mice injected with preformed Pt^IV^NPs displayed strong NIR FL throughout the whole liver and gastrointestinal tract at 1 h, which was difficult to pinpoint the tumor locations. Thereafter, the liver FL fast declined, and the FL intensity in the orthotopic liver tumor was significantly ~2.7-fold lower than that in the P-CyPt-treated mice at 4 h (Fig. 5b). These results suggest that P-CyPt could be efficiently activated and produce strong NIR FL to accurately locate the orthotopic liver tumors, which was more efficient than preformed Pt^IV^NPs.

Guided by the imaging results, therapy of orthotopic HepG2/Luc liver tumors with P-CyPt, CDDP, Pt^IV^NPs or PBS was conducted (Fig. 5c and Supplementary Fig. 34). BL imaging showed that the tumor BL in P-CyPt-treated mice did not obviously increase over the course of treatment, while the BL in PBS-, CDDP- or Pt^IV^NPs-treated tumors all markedly increased (Fig. 5d). On day 15, the average BL intensity in PBS-, CDDP- and Pt^IV^NPs-treated tumors was significantly ~13.0-, ~10.7- and ~5.7-fold higher than that in P-CyPt-treated tumors, respectively (Fig. 5e). Ex vivo BL imaging affirmed that the tumor size in each P-CyPt-treated mouse was significantly smaller than that in each PBS-, CDDP- or Pt^IV^NPs-treated mouse (Fig. 5f). No metastatic tumor foci appeared in the livers of P-CyPt-treated mice, contrary to that in PBS-, CDDP- or Pt^IV^NPs-treated mice. After BL imaging, ex vivo FL imaging by spraying P-CyPt on these livers showed bright mCy’s FL that clearly delineated the tumor locations in livers (Fig. 5g). Note that a strong correlation between the tumor FL intensity and BL intensity was achieved in these four groups of livers, showcasing the potential use of P-CyPt for fluorescence-guided surgery of residual liver tumors after chemotherapy^42,43^ (Fig. 5h).

### Evaluation of pharmacokinetics, metabolism, clearance, and biosafety of P-CyPt

**Fig. 6:**
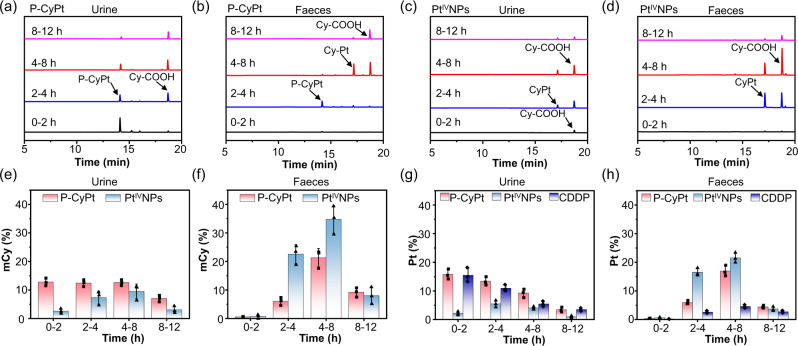
Evaluation of the metabolism, clearance, and biosafety of P-CyPt in vivo. **a**–**d** HPLC traces (660 nm detection) of urine (**a**, **c**) and faeces (**b**, **d**) collected from mice receiving i.v. injection of P-CyPt or Pt^IV^NPs. Healthy nude mice were i.v. injected with P-CyPt or Pt^IV^NPs (2.25 mg kg^−1^ Pt), and the urine and faeces were collected in 0–2, 2-4, 4–8, and 8–12 h. **e**, **f** Quantification of the amount of mCy-containing compounds (%) in the urine (**e**) and faeces (**f**) of mice during 0–2, 2–4, 4–8, and 8–12 h following i.v. injection of P-CyPt or Pt^IV^NPs. **g**, **h** Quantification of the amount of Pt element in the urine (**g**) and faeces (**h**) of mice during 0–2, 2–4, 4–8, and 8–12 h following i.v. injection of P-CyPt, Pt^IV^NPs, or CDDP (2.25 mg kg^−1^ Pt). The amount of mCy-containing compounds and Pt elements were quantified by HPLC and ICP-OES analysis, respectively. Data denote mean ± s.d. (*n* = 3 independent animals for **e**–**h**).

We first examined the pharmacokinetics of P-CyPt, Pt^IV^NPs, and CDDP following i.v. injection into mice. It was found that P-CyPt as a hydrophilic small-molecule probe held a similar blood circulation half-life (*t*_1/2_ = 0.54 ± 0.06 h) to CDDP (0.73 ± 0.04 h), which was shorter than Pt^IV^NPs (1.65 ± 0.23 h) (Supplementary Fig. 35). Next, HPLC analysis of urine and faeces of P-CyPt-treated mice showed that P-CyPt was majorly observed in the urine (~11.3%), not in the faeces within 0–2 h, while both P-CyPt and Cy-COOH were obviously present in the urine within 2–4 h and 4–8 h (Fig. 6a and Supplementary Table 5). In the faeces, some P-CyPt (~4.5%) appeared within 2–4 h, but CyPt (~9.7%) and Cy-COOH (~11.1%) predominantly appeared within 4–8 h (Fig. 6b and Supplementary Table 6). In the following 8–12 h, Cy-COOH dominated in both urine and faeces. By contrast, mice injected with Pt^IV^NPs showed that CyPt and Cy-COOH were mainly present in the faeces within 2–4 h and 4–8 h (Fig. 6c, d). Quantification of mCy-containing compounds showed that ~44.8% and ~37.0% of injected P-CyPt were respectively excreted via renal and hepatobiliary system over 12 h; however, ~65.9% of Pt^IV^NPs were excreted via the hepatobiliary system, much larger than that via the renal system (~22.4%) (Fig. 6e, f and Supplementary Tables 7, 8). ICP-OES analysis revealed that the Pt-containing compounds were mainly cleared to the urine in P-CyPt-treated mice within 0–2 h, and then cleared via both renal and hepatobiliary pathways (Fig. 6g, h), matching that of HPLC analysis. As a comparison, the Pt was excreted majorly via the renal system in CDDP-treated mice, and via the hepatobiliary system in Pt^IV^NPs-treated mice. Note that ~69.4% of Pt could be accumulatively cleared from the P-CyPt-treated mice during 12 h, much higher than that in CDDP-treated (~45.3%) or Pt^IV^NPs-treated mice (~55.0%) (Supplementary Tables 9 and 10). These results demonstrate that P-CyPt was more efficient in clearing Pt from normal organs compared with free CDDP or preformed Pt^IV^NPs, which could be important in mitigating systemic toxicities of CDDP^18,44^ (Supplementary Fig. 36).

## Discussion

Selective delivery and control release of drugs in tumor tissues are of prime importance in improving therapeutic efficacy while reducing side-toxicity to patients in clinic^45^. Though many intelligent nanocarriers have been designed to potentiate drug delivery into tumors, the dense extracellular matrix and high interstitial fluid pressure in solid tumors have presented significant barriers for nanomedicines^5,46–48^, which generally resulted in <1% of systemically administrated nanodrugs that could accumulate in tumor tissues^25^. To augment tumor accumulation of administrated drugs for improving anticancer efficacy, here, we have presented a stimuli-triggered sequential in situ self-assembly and disassembly strategy to temporally control delivery and release of anticancer drugs in tumors following systemic administration. Different from previously reported nanomedicines, this strategy markedly enhances tumor accumulation via ALP-triggered in situ self-assembly of a small-molecule prodrug (e.g., P-CyPt), and achieves a burst release of parent drug molecules (e.g., CDDP) via succeeding GSH-driven disassembly of in situ formed NPs, consequently eliciting a strong therapeutic efficacy in mice with s.c. HeLa tumors and orthotopic HepG2 liver tumors. Moreover, this strategy also provides activated NIR FL and PA bimodal imaging signals to enable high sensitivity and spatial-resolution visualization of the tumors as well as real-time monitoring of drug delivery and release in vivo for guiding chemotherapy^35^.

We have utilized this strategy to design P-CyPt, which holds many advantages to manipulate CDDP delivery for improving treatment efficacy. P-CyPt is a water soluble small-molecule prodrug able to overcome the barriers of solid tumors, ensuring faster extravasation and deeper penetrability into tumor tissues as compared with conventional nanocarrier-based drug delivery systems (e.g., Pt^IV^NPs, Fig. 3k). ALP-triggered dephosphorylation and in situ self-assembly of P-CyPt into Pt^IV^NPs helps them to adhere on cell membranes (where the ALP locates) and enhance cellular uptake (via clathrin-dependent endocytosis), permitting higher accumulation in the tumor tissues than that of free CDDP or preformed Pt^IV^NPs. The intracellular GSH-mediated reduction and disassembly of in situ formed Pt^IV^NPs affords a burst release of CDDP intracellularly, which augments CDDP’s concentration (24.60 ± 3.5 fmol/cell, Fig. 3g), enhances delivery into nucleus and mitochondria (Fig. 3h), and concurrently depletes GSH’s levels in tumor cells (Supplementary Table 2), promising to synergize cytotoxicity and overcome drug resistance. P-CyPt is highly specific for tumors as it is activated by a combination of membrane-bound ALP and endogenous GSH, both of which are upregulated in many tumor cells (e.g., HeLa, HepG2)^49,50^. It is noteworthy that P-CyPt leverages the enzymatic catalysis of ALP that triggers continuous dephosphorylation and in situ self-assembly of P-CyPt, allowing to trap many activated molecules in tumor tissues (Fig. 4f). Finally, P-CyPt harnesses both renal and hepatobiliary systems to enhance excretion from body (Fig. 6e–h), essential to lower systemic toxicities of CDDP following multiple dosing regimens^51^.

In addition to efficiently treat mice with s.c. HeLa and orthotopic HepG2 liver tumors, P-CyPt was also capable of lighting up tumor cells and serially monitoring the accumulation and release of CDDP in tumors via NIR FL and PA bimodality imaging. The tumor FL reached the maximum at 1 h, and persisted for 4 h after i.v. injection of P-CyPt, (Fig. 4a), implying the rapid activation and accumulation of P-CyPt in the tumors. Meanwhile, we observed that the tumors displayed strong dual PA imaging signals (at 700 and 750 nm) at 2 h post-injection of P-CyPt; however, the PA signal at 700 nm remained strong (similar to that of tumor FL) but the PA signal at 750 nm in tumors declined to the baseline at 4 h (Fig. 4b). These dual PA imaging results indicated the formation of Pt^IV^NPs in the tumor tissues at 2 h, which then mostly disassembled into Cy-COOH that was resided in the tumors at 4 h, matching that of HPLC analysis (Fig. 4e). P-CyPt with dual PA imaging performance could provide us a powerful tool to noninvasively monitor the in situ self-assembly and disassembly process, which mirrored the delivery and release of CDDP in the tumors. Moreover, P-CyPt was also found capable of producing sensitive NIR FL images to precisely pinpoint the tumor foci by simply spraying it on the livers, which reported efficient inhibition of orthotopic liver tumor growth and metastasis in P-CyPt-treated mice (Fig. 5f). These sensitive and localized NIR FL signals may provide valuable information to monitor therapeutic efficacy and guide liver tumor surgery in clinic. This, in conjunction with noninvasive dual PA imaging and largely improved treatment outcomes, suggests that P-CyPt is highly efficient in improving cancer theranostics by leveraging the stimuli-triggered sequential in situ self-assembly and disassembly.

In conclusion, we report a strategy by leveraging extracellular ALP-triggered in situ self-assembly and intracellular GSH-driven disassembly, and demonstrate its utility for the design of a fluorogenic small-molecule cisplatin prodrug (P-CyPt) for cancer theranostics. We have performed a series of experiments to interrogate this sequential in situ self-assembly and disassembly process in vitro and in vivo. The ALP-triggered in situ self-assembly and GSH-triggered intracellular disassembly of P-CyPt allows for temporal control of cisplatin delivery and release in tumors, substantially improving antitumor efficacy while mitigating off-target toxicity in mice with orthotopic HepG2 liver tumors. P-CyPt was also highly feasible for the detection of tumor foci and noninvasive monitoring of drug release via synergetic combination of NIR FL and dual PA imaging. This strategy may also be adopted to the construction of other stimuli-responsive small-molecule theranostic probes capable of improving imaging and treatment of cancer and other malignant diseases^11,52^.

## Methods

### Synthesis and characterization of probes

Detailed procedures for the synthesis of P-CyPt, CyPt, and Cy-COOH were described in the Supplementary Methods (Supplementary Figs. 37–39). After purification by semi-prepared HPLC (Supplementary Tables 11–13), their chemical structures were characterized by NMR and mass spectroscopy (Supplementary Figs. 40–65).

### Evaluation of the self-assembly and disassembly processes in solutions

To evaluate the ALP-triggered dephosphorylation and in situ self-assembly process, a solution of P-CyPt (10 μM) in Tris buffer (1 mL) was incubated with ALP (100 U/L) at 37 °C. HPLC, DLS, and TEM analyses were performed at 0, 5, 10, 15, 20, and 30 min. To evaluate the GSH-triggered reduction and disassembly process, P-CyPt (10 μM) in Tris buffer (1 mL) was first incubated with ALP (100 U/L) at 37 °C for 30 min to allow the formation of Pt^IV^NPs. The solution was then treated with 10 mM GSH at 37 °C, which was monitored by HPLC, DLS, and TEM at 10, 20, 30, 40, 50, and 60 min.

### Monitoring of the NIR FL and PA imaging signals in solutions

To monitor the NIR FL and PA imaging signals of P-CyPt in response to ALP, P-CyPt (10 μM) in Tris buffer (1 mL) was incubated with ALP (100 U/L) at 37 °C for 0, 5, 10, 15, 20, and 30 min. To monitor the NIR FL and PA imaging signals of Pt^IV^NPs in response to GSH, P-CyPt (10 μM) in Tris buffer (1 mL) was first incubated with ALP (100 U/L) at 37 °C for 30 min and then incubated with 10 mM GSH at 37 °C for another 10, 20, 30, 40, 50, and 60 min. The NIR FL spectra of these solutions at each time point were acquired on a HORIBA Jobin Yvon Fluoromax-4 fluorescence spectrometer, with excitation at 680 nm. For PA imaging, the incubation solutions at each time point were loaded into a fine bore polythene tube (0.86 mm OD, 1.27 mm OD). The tubes were then sealed and immersed in water. The PA images at both 700 and 750 nm were acquired on the Vevo 2100 LAZR system (FUJIFILM VisualSonics).

### Cell culture

Human cervical cancer HeLa cells, human liver hepatocellular carcinoma HepG2 cells and human embryonic kidney HEK293T cells were obtained from Stem Cell Bank, Chinese Academy of Sciences (Shanghai, China). All cells were cultured in DMEM (Dulbecco’s Modified Eagle Medium) medium, and routinely tested for mycoplasma contamination. The mediums were supplemented with 10% (v/v) fetal bovine serum (FBS), 100 units /mL penicillin, and 100 units/mL streptomycin. All cells were cultured at 37 °C in a humidified atmosphere (5% CO_2_).

### NIR FL and PA bimodality imaging of cell pellets

To examine the ability for NIR FL and PA bimodality imaging of ALP activity in cells, HeLa or HEK29T cells were seeded in 10-cm dishes at a density of 4 × 10^6^ cells/well and allowed to grow overnight. Then, P-CyPt (10 μM) or Pt^IV^NPs (10 μM) in FBS free DMEM (4 mL) was added into wells and incubated at 37 °C for 30 min. To inhibit the ALP activity, cells were pretreated with ALP inhibitor Na_3_VO_4_ (10 mM) for 20 min, and then incubated with P-CyPt (10 μM) for another 30 min. To examine NIR FL and PA bimodality imaging of intracellular GSH-triggered disassembly process, P-CyPt (10 μM) in FBS free DMEM (4 mL) was added into HeLa cells, and incubated at 37 °C for 30 min. Then, the medium was removed and the cells were incubated in refreshed DMEM medium (10% FBS) for another 3 h. Then, the mediums of above mentioned cells were removed, and the cells were washed with PBS (1 mL) once. Trypsin (1 mL) was added to detach the cells, and the cell pellets were then collected after centrifugation at 161 × *g* for 4 min. The NIR FL images (*λ*_ex/em_ = 670/750 ± 50) of the cell pellets were acquired on the IVIS Lumina XR III system (PerkinElmer), and the PA images of the cell pellets at both 700 and 750 nm were acquired on the Vevo 2100 LAZR system (FUJIFILM VisualSonics).

### Evaluation of cytotoxicity

HeLa cells were seeded on flat-bottomed 96-well plates (5000 cells per well) and incubated at 37 °C overnight. Varying concentrations of P-CyPt, Pt^IV^NPs or CDDP (0, 1, 2, 5, 10, 20, 30, 50, 100, and 200 μM) in DMEM medium (100 μL) were added. Cells were either incubated for 48 h, or firstly incubated with these three compounds for 2 h, washed with PBS and then incubated with refreshed blank DMEM medium for another 48 h. Then, 3-(4,5)-dimethylthiahiazo (-z-y1)−3,5-di-phenytetrazoliumromide (MTT, 50 μL, 1 mg /mL in PBS) was added into each well. The cells were kept at 37 °C for 4 h, and the solution in each well was carefully removed, followed by addition of dimethyl sulfoxide (DMSO, 150 μL). The absorbance (OD) at 490 nm in each well was acquired on the microplate reader (Tcan). The absorbance of non-treated blank cells (OD control) was used as the control. The percentage of cell viability was calculated by dividing OD to the OD control.

### Animals and tumor models

The animal experiments were approved by the Institutional Animal Care and Use Committee (IACUC) of Nanjing University (Approval No: IACUC-2107004), and carried out under the guidelines of the IACUC of Nanjing University. BALB/c female nude mice at 5–6 weeks old and 6–8 weeks old were purchased from the Model Animal Research Center (MARC) of Nanjing University (Nanjing, China), and were grouped and housed under a 12 h light-dark cycle, 50–70% humidity, and at 18–22 °C ambient temperature, with free access to water and food. To establish subcutaneous (s.c.) HeLa tumors, 2 × 10^6^ HeLa cells suspended in 100 μL of 33 v/v% mixture of matrigel and DMEM were injected s.c. into the selected positions of nude mice (5–6 weeks’ old). The tumors were allowed to grow for around 10 days to reach the size of around 100 mm^3^, which were used for FL and PA imaging. To evaluate the therapeutic efficacy, the tumors were allowed to grow for around 7 days to reach the size of around 70~80 mm^3^. To establish the orthotropic hepatocellular carcinoma (HCC) tumors, nude mice under anesthesia (by pentobarbital sodium) were performed with a midline incision on the anterior abdominal wall, and 2 × 10^6^ luciferase-transfected HepG2 (Luc/HepG2) cells suspended in 100 μL Matrigel and DMEM (33 v/v%) were directly injected into the left lobe of the liver. The growth of the orthotopic HepG2 tumors in mice was monitored by bioluminescence imaging (BLI). After 10 days, the orthotropic liver tumors were successfully established.

### NIR FL and PA bimodality imaging of tumors in vivo

For NIR FL imaging in vivo, mice with s.c. HeLa tumors or orthotopic Luc/HepG2 liver tumors were i.v. injected with P-CyPt or Pt^IV^NPs (100 μM, in 200 μL saline). Whole body FL images of mice prior to (pre) and at 1 h, 2 h, 4 h, 8 h post injection of P-CyPt or Pt^IV^NPs were acquired on the IVIS Lumina XR III imaging system, using a 660 nm excitation filter and a 750 ± 50 nm emission filter. Each experiment was conducted in three mice. The FL intensities were quantified by the region of interest (ROI) measurement using Living Image Software (4.5.2, PerkinElmer, MA, U.S.A). For PA imaging, mice with s.c. HeLa tumors were i.v. injected with P-CyPt or Pt^IV^NPs (500 μM, in 200 μL saline). The PA images of the s.c. HeLa tumors in mice prior to (pre), and at 1, 2, 4 h post injection were captured on the Vevo 2100 LAZR system (FUJIFILM VisualSonics), with excitation at 700 and 750 nm. The PA intensities were quantified by the equipped software.

### Chemotherapy of s.c. HeLa tumors in vivo

Sixteen mice with s.c. HeLa tumor at a size of 70–80 mm^3^ were randomly divided into four groups, which were received i.v. injection of PBS (200 μL, Group 1), CDDP (Group 2), Pt^IV^NPs (Group 3), and P-CyPt (Group 4), respectively. The dose of Pt drug for each injection was 2.25 mg kg^−1^ Pt), and the treatment was performed on day 0, 3, 6, 9, and 12, with a total of 5 injections. The tumor volumes and body weights of mice were measured every three days, and lasted for 21 days. On day 21, all the mice were sacrificed, and the tumors were excised and photographed. Each experiment was conducted in four mice.

### Chemotherapy of orthotopic liver tumors

Sixteen mice with orthotopic Luc/HepG2 tumors were randomly divided into four groups: Group 1 (PBS only), Group 2 (CDDP), Group 3 (Pt^IV^NPs), and Group 4 (P-CyPt). PBS (200 μL) or each Pt drug (2.25 mg kg^−1^ Pt) was i.v. injected into mice on day 0, 3, 6, 9, and 12, with a total of 5 injections. To monitor the therapeutic effect, mice were intraperitoneally (i.p.) injected with _*D*_-luciferin (150 mg/kg), and after 10 min, the whole body BL images of mice (face-up) were acquired on the IVIS Lumina XR III system using BL imaging mode (Open). The BL imaging was repeated every 3 days and lasted for 15 days. The BL intensities were quantified by the ROI measurement in the liver region using the Living Image Software (4.5.2, PerkinElmer, MA, U.S.A). During the treatment, the body weights of mice were also measured every three days. On day 15, all mice were sacrificed, the whole liver were excised and photographed. Then, P-CyPt (10 μM) and _*D*_-luciferin (5 mM) were sprayed on the surface of each liver to performed ex vivo BL and NIR FL imaging. The BL images were acquired at 10 min using the BL imaging mode (Open), and the NIR FL images were acquired at 30 min, using a 660 nm excitation filter and a 750 ± 50 nm emission filter. The average BL and FL intensities were quantified by the ROIs measurement in the tumor region of liver using the Living Image Software (4.5.2, PerkinElmer, MA, U.S.A). Each experiment was conducted in four mice.

### Statistical analysis

Statistical comparisons between two groups were performed by two-sided Student’s *t*-test, and analyzed on Prism 6 (GraphPad Software, Inc., CA, USA). Results are present as mean ± s.d. and *p* < 0.05 was considered statistically significant.

### Reporting summary

Further information on research design is available in the Nature Portfolio Reporting Summary linked to this article.

## Supplementary information

Supplementary Information

Reporting Summary

## Data Availability

The data that support the findings of this study are available within the main text and its Supplementary Information file. Source data is provided as Source Data file. Data is also available from the corresponding author upon request. Source data are provided with this paper.

## Source data

Source Data
