# Supplementary material for: Controlled sequential in situ self-assembly and disassembly of a fluorogenic cisplatin prodrug for cancer theranostics
Source: Nat Commun. 2023 Feb 13;14:800. doi: 10.1038/s41467-023-36469-1 (PMC9925730; doi:10.1038/s41467-023-36469-1)
Supplement: Supplementary file 1 — Supplementary Information [file 41467_2023_36469_MOESM1_ESM.pdf]

Supplementary Information

Controlled sequential in situ self-assembly and disassembly of a fluorogenic cisplatin prodrug for cancer theranostics

Xidan Wen[1], Rui Zhang[2], Yuxuan Hu[1], Luyan Wu[1], He Bai[1], Dongfan Song[3], Yanfeng Wang[4], Ruibing An[1], Jianhui Weng[1], Shuren Zhang[3], Rong Wang[3], Ling Qiu[5], Jianguo Lin[5], Guandao Gao[4], Hong Liu[2,*], Zijian Guo[3,*] and Deju Ye[1,*]

[1] State Key Laboratory of Analytical Chemistry for Life Science, Chemistry and Biomedicine Innovation Center (ChemBIC), School of Chemistry and Chemical Engineering, Nanjing University, 163 Xianlin Road, Nanjing, 210023, China.

[2] State key Laboratory of Drug Research, Shanghai Institute of Materia Medica, Chinese Academy of Sciences, 555 Zu Chong Zhi Road, Shanghai, 201203, China.

[3] State Key Laboratory of Coordination Chemistry, Chemistry and Biomedicine Innovation Center (ChemBIC), School of Chemistry and Chemical Engineering, Nanjing University, 163 Xianlin Road, Nanjing, 210023, China.

[4] State Key Laboratory of Pollution Control and Resource Reuse, School of Environment, Nanjing University, 163 Xianlin Road, Nanjing, 210023, China.

[5] NHC Key Laboratory of Nuclear Medicine, Jiangsu Key Laboratory of Molecular Nuclear Medicine, Jiangsu Institute of Nuclear Medicine, Wuxi 214063, China.

E-mail: hliu@simm.ac.cn; zguo@nju.edu.cn; dejuye@nju.edu.cn

Table of Content

		Page
1.	Supplementary Figures 1-36	3-26
2.	Supplementary Tables 1-13	27-31
3.	Supplementary Materials and Instruments	32-33
4.	Supplementary Methods	33-46
5.	Supplementary Figure 37-39	33-36
6.	Supplementary Figure 40-65	47-64

1. Supplementary Figures 1-36

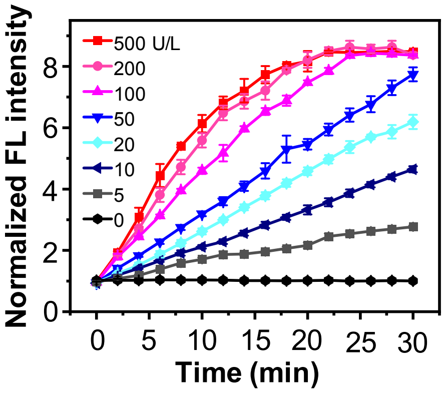

Supplementary Figure 1. Monitoring the NIR fluorescence of P-CyPt in response to ALP. Normalized NIR fluorescence intensity ($\lambda_{ex/em}$ = 680/710 nm) of P-CyPt (10 μM) upon incubation with varying concentrations (0-500 U/L) of ALP in Tris buffer for 0-30 min, revealing that the NIR FL reached a plateau upon incubation with 100 U/L ALP. Data are mean ± s.d. (n = 3 independent experiments).

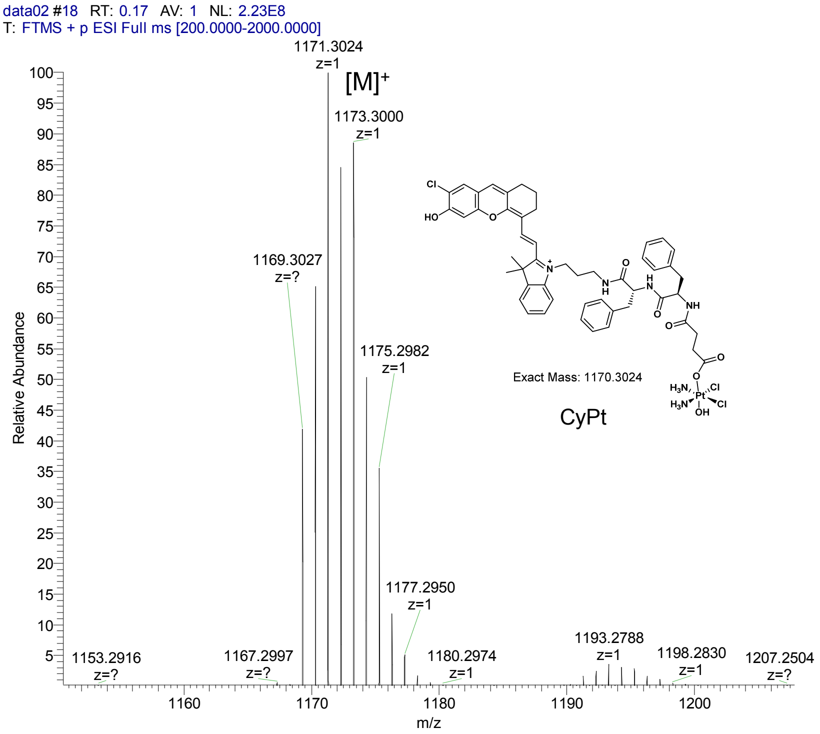

Supplementary Figure 2. Characterization of the dephosphorylated product. High resolution ESI-MS spectra of **P-CyPt** following incubation with ALP. The molecular weight of CyPt (calcd. For $C_{50}H_{58}Cl_3N_6O_8Pt^+$[$M^+$]:1169.3003(97.4%), 1170.3024(100.0%),1170.3037(52.7%),1171.2974(93.4%),1171.3026(74.6%),1171.3058(54.1%),1172.2995(95.9%),1172.3007,(50.5%),1172.3060(40.3%),1173.2944(29.9%),1173.3028(51.9%),1173.3055(21.2%),1174.2965(30.6%),1174.3030(38.7%),1173.2997(71.5%),1175.2967(22.9%),1175.3026 (20.3%)), which was found at 1169.3027, 1170.3044, 1171.3024, 1172.3016, 1173.3000, 1174.2998 and 1175.2982.

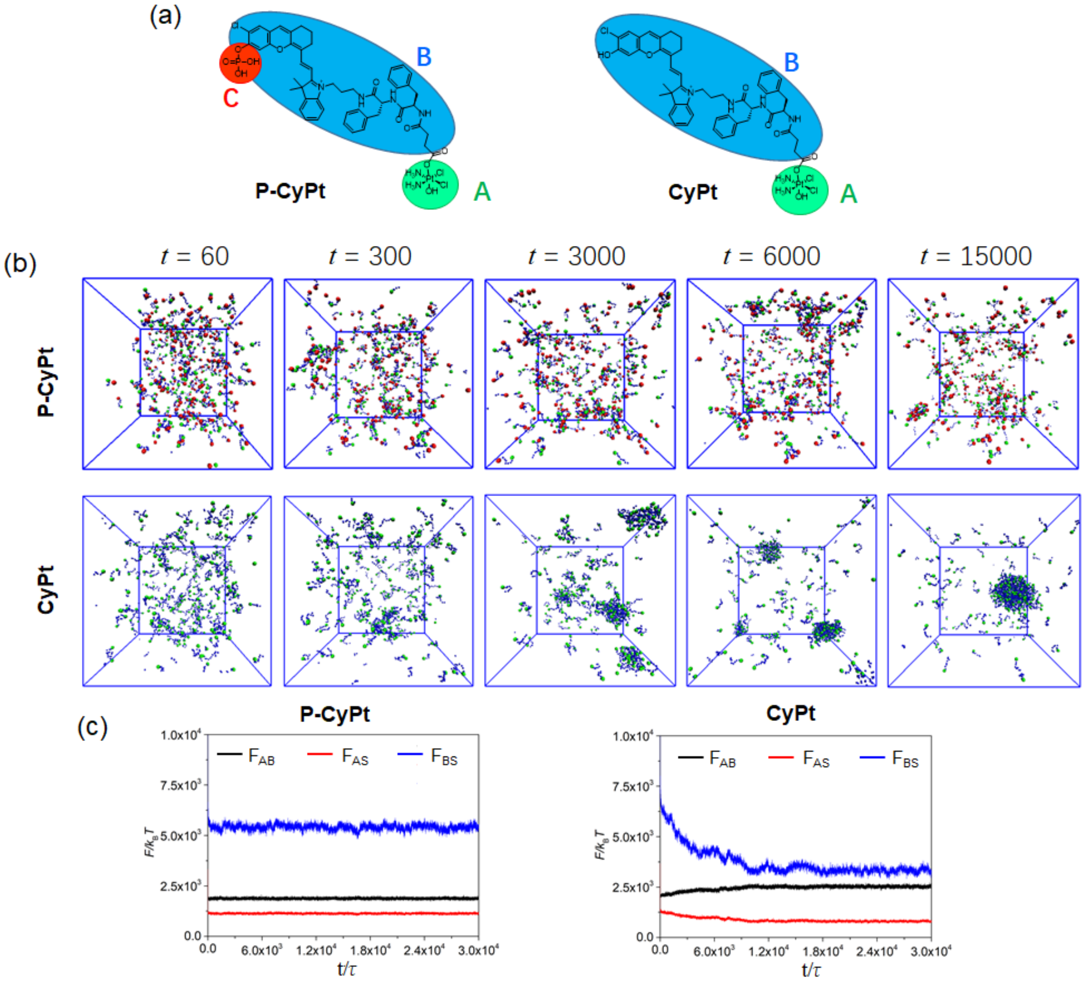

Supplementary Figure 3. Dissipative particle dynamics (DPD) simulation of CyPt or P-CyPt. (a) The generic coarse-grained model of P-CyPt and CyPt. A, B and C represent Pt(IV) component (green), mCy group (blue) and PO_3H group (red), respectively. (b) The snapshots of the simulated aggregation process of P-CyPt and CyPt when t = 60, 300, 3000, 6000 and 15000 (τ), respectively. The green, blue, and red colors represent A, B, and C; the solvent beads (S, water here) are not shown for clarity. (c) The changes of interfacial energies (*F*) between different components of P-CyPt and CyPt during simulation from 0 – 3.0 × 10^4 (τ). F_{AB}, F_{AS} and F_{BS} indicate the interfacial energy between A and B, A and S, B and S, respectively. These results demonstrated that CyPt could easily assemble into nanoaggregates, while P-CyPt was kept at a disperse state in water.

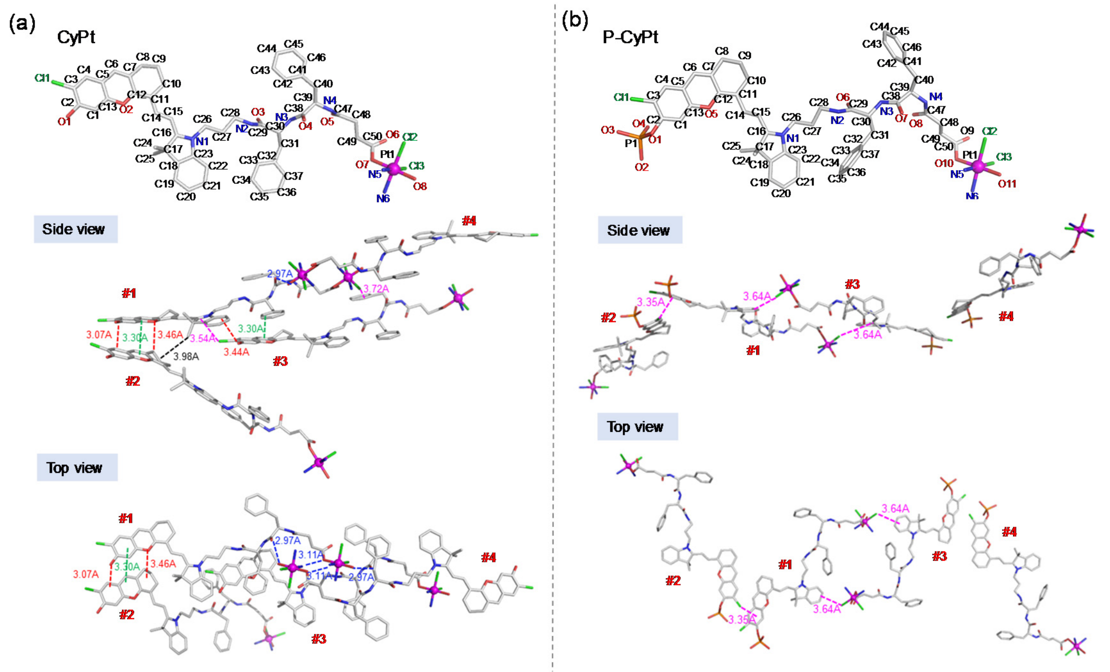

Supplementary Figure 4. Evaluation of the molecular interactions between CyPt (a) **and P-CyPt** (b) **in theoretically simulated structures**. The structures (shown in stick mode) of simulated CyPt and P-CyPt, in which the numbers of atoms C (grey), Cl (green), O (red) , N (blue) and Pt (magenta) were labeled H atoms were omitted for clarity. The side view (right) and top view of the optimum molecular packing mode of CyPt and P-CyPt showed four representative CyPt molecules in the $P2_1/c$ space group. There are strong intermolecular interactions existing among the closely packed CyPt moleculesincluding: (1) two O···π interactions (red dashed lines, side view), π-π stacking (green dashed lines, side view) and van der Waals interaction (black dashed lines, side view) between two adjacent mCy groups in CyPt #1 and #2; (2) Cl···π interaction (magenta dashed lines, side view), and O···π interaction (red dashed lines, side view) between the mCy group in CyPt #3 and the indole group in CyPt #1; (3) π-π stacking (green dashed lines, side view) between the Phe side chain in CyPt #1 and the mCy group in CyPt #3; (4) Cl···π interaction (magenta dashed lines, side view) between the Cl_3 in CyPt #1 and the Phe side chain in CyPt #3; (5) four hydrogen bonding interactions (blue dashed lines, top view) between the two Pt(IV) prodrug groups in CyPt #1 and #4. These strong intermolecular interactions help CyPt to easily assemble into spherical nanoparticles. By contrast, much fewer interactions between P-CyPt molecules, probably due to that the hydrophilic phosphate group prevents π-π stacking between the mCy scaffolds in two adjacent P-CyPt molecules. These molecular interactions mainly include (1) one Cl···π interaction (side view) between the Cl in P-CyPt #2 and mCy group in P-CyPt #1; (2) two Cl···π interactions (side view) between the mCy group and Pt(IV) prodrug group in P-CyPt #1 and #3, respectively. There is no hydrogen bonding interaction between P-CyPt #1 and #3, and no any interaction exists between P-CyPt #1 and #4. Thus, P-CyPt was difficult to assemble into nanoparticles due to the substantially decreased intermolecular interactions as compared to CyPt.

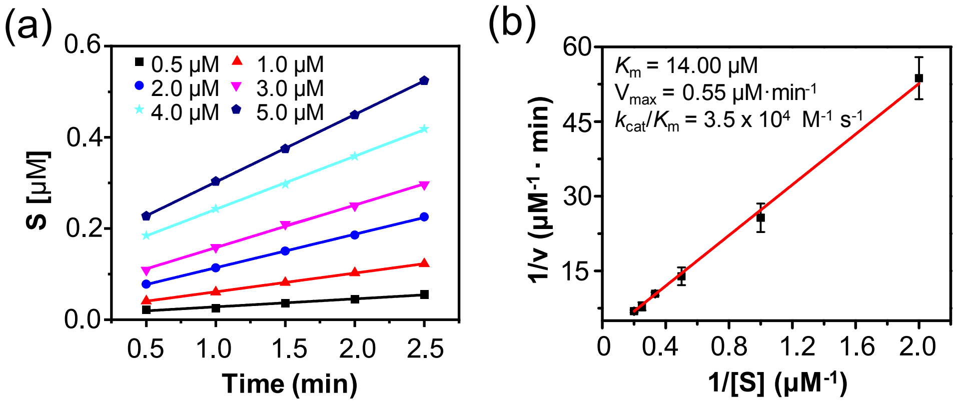

Supplementary Figure 5. Evaluation of the enzymatic kinetics of P-CyPt towards ALP. (a) Plots of the concentrations of CyPt versus incubation time when incubating different concentrations of P-CyPt with ALP (20 U/L). (b) Lineweaver-Burk plots of 1/V vs. 1/[S] for P-CyPt. Values denote mean ± standard deviation (s.d.) (n = 3 independent experiments).

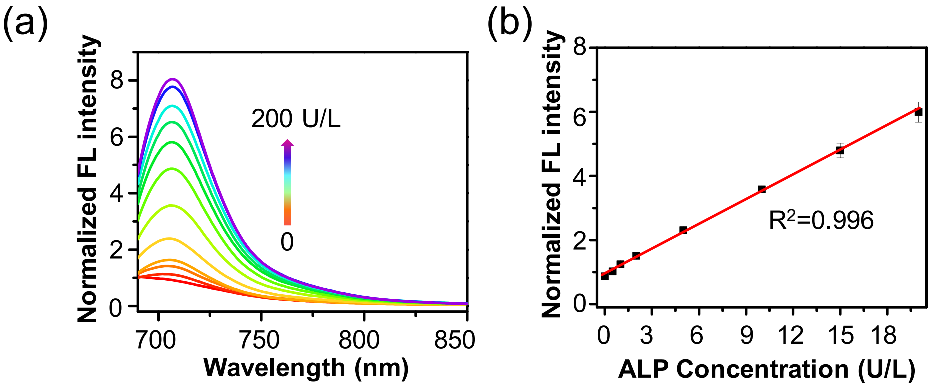

Supplementary Figure 6. Evaluation of the sensitivity of P-CyPt toward ALP. (a) Fluorescence spectra of **P-CyPt** (10 μM) upon incubation with varying concentrations (0-200 U/L) of ALP for 30 min. (b) Linear fitting curve of the fluorescence intensity (λ_{em} = 710 nm) versus the concentration of ALP from 0-20 U/L. Data denote mean ± s.d. (n = 3 independent experiments). The detection limit was found to be ~0.098 U/L, using a 3σ/k method.

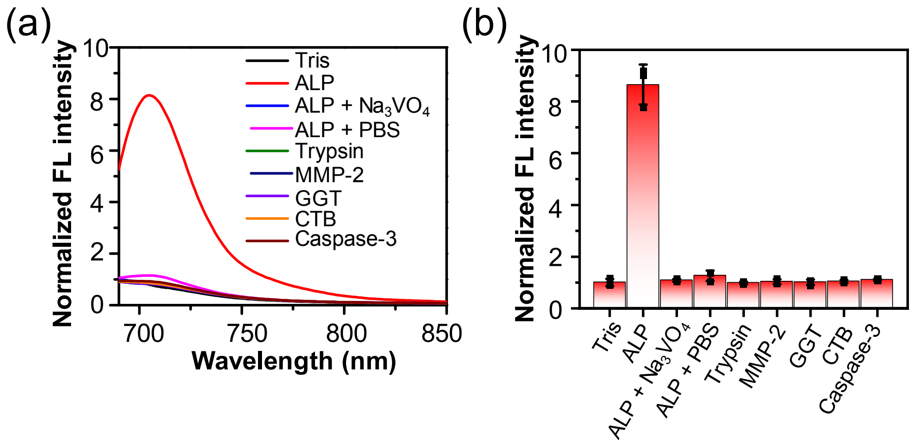

Supplementary Figure 7. Evaluation of the specificity of P-CyPt toward ALP. (a) Fluorescence spectra and (b) normalized FL intensity of P-CyPt (10 μM) incubated with Trypsin (2.5 mg/mL), MMP-2 (10 nmol/L), γ-glutamyl transferase (GGT, 100 U/L), Cathepsin B (CTB,100 U/L), Caspase-3 (0.2 μg/mL), ALP (100 U/L), ALP plus PO_4^{3-} (using PBS buffer) or ALP together with its inhibitor Na_3VO_4 (100 μM) in Tris buffer (pH = 8.0). Data denote mean ± s.d. (n = 3 independent experiments).

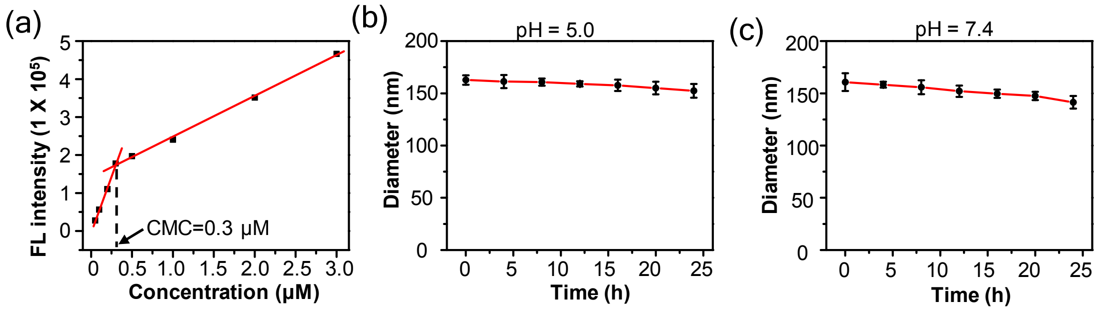

Supplementary Figure 8. Evaluation of the CMC and stability of Pt^{IV}NPs under different pH conditions. (a) Plots of the fluorescence intensity (λ_{em} = 710 nm) versus the concentration of CyPt in Tris buffer determine the CMC of CyPt, which was found to be ~0.3 μM according to the intersection of the two linear fit lines. (b, c) Monitoring of the hydrodynamic size of Pt^{IV}NPs upon incubation under (b) pH = 5.0, and (c) pH = 7.4 (10 μM) for 0-24 h. Date denote mean ± s.d. (n = 3 independent experiments).

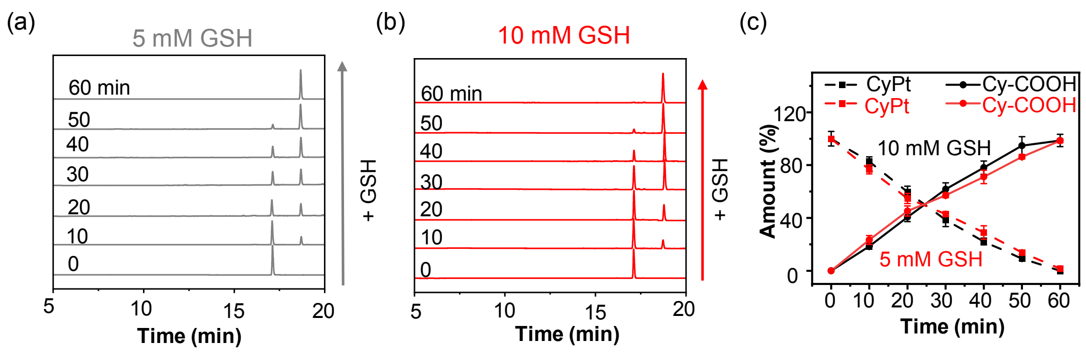

Supplementary Figure 9. HPLC traces of the time-dependent conversion of PtIVNPs (CyPt) into Cy-COOH under incubation with 5 mM (a) or 10 mM GSH (b) in Tris buffer. (c) The conversion curves of Pt^{IV}NPs (CyPt) into Cy-COOH under incubation with 5 mM (red) or 10 mM GSH (black) in Tris buffer. P-CyPt (10 μM) was incubated with ALP (100 U/L, 37 ºC) in Tris buffer for 30 min to allow complete dephosphorylation and in situ self-assembly into Pt^{IV}NPs. Then, GSH at a concentration of 5 mM or 10 mM was added and incubated for another 0, 10, 20, 30, 40, 50, and 60 min. The amounts of CyPt and Cy-COOH at each time point were quantified by HPLC. Date are mean ± s.d. (n = 3 independent experiments).

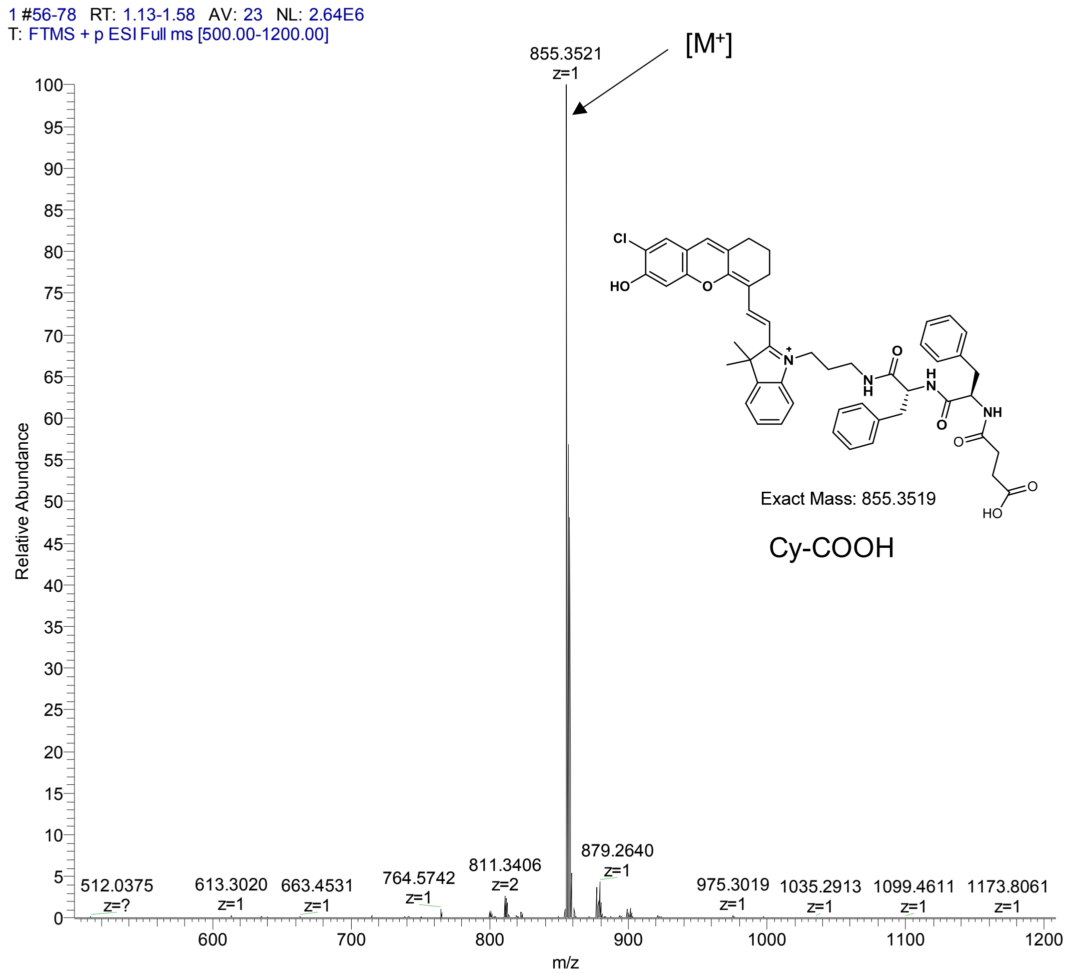

Supplementary Figure 10. ESI-MS analysis of the reduced product Cy-COOH. P-CyPt (10 μM) was treated with ALP (100 U/L) at 37 ºC for 30 min, then added with 10 mM GSH, and incubated for another 60 min. The solution was applied for ESI-MS analysis, with the molecular weight found to be 855.3521 [M^+], which is identical to that of Cy-COOH (calcd. For$C_{50}H_{52}ClN_4O_7^+$ [M^+]: 855.3519).

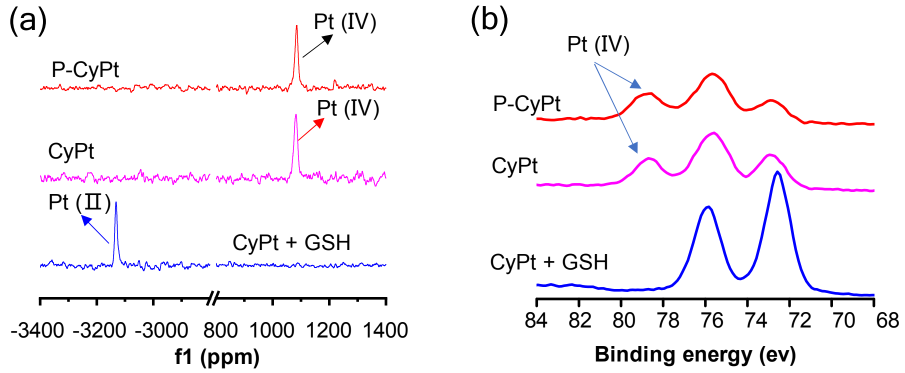

Supplementary Figure 11. Characterization of the reduction of Pt (IV) into Pt (II) by GSH. (a) ^{195}Pt NMR spectra of P-CyPt (30 mM in DMSO-*d*6), CyPt (30 mM in DMSO-*d*6) and CyPt (30 mM) following incubation with GSH (240 mM) in DMSO-*d*6/D_2O (80/20%) solution at 37 ºC for 12 h. (b) XPS spectra of P-CyPt (2 mg), CyPt (2 mg) and CyPt (2 mg) following incubation with GSH (100 mM) at 37 ºC for 60 min.

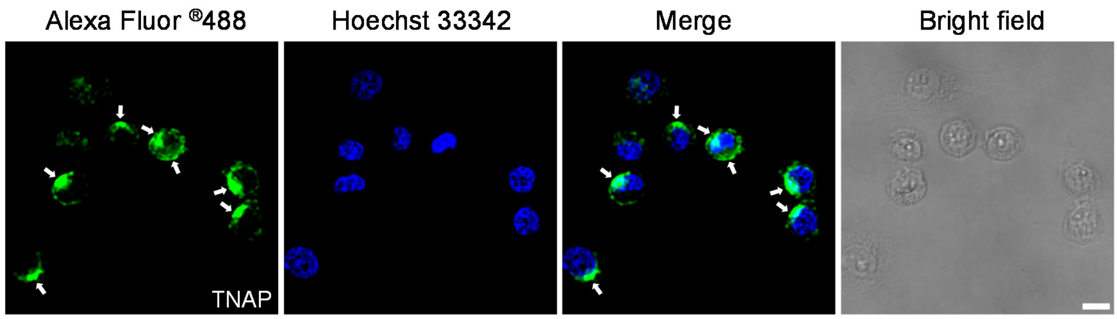

Supplementary Figure 12. Immunofluorescence staining of ALP expressed on the membranes of HeLa cells. HeLa cells were first stained with the primary antibody of ALP (tissue nonspecific ALP (TNAP)), and then stained with the Alexa Fluor®488-labeled secondary antibody (goat anti-rabbit IgG (H+L)). Nuclei were then stained with Hoechst 33342. The confocal fluorescence images were then acquired at Alexa Fluor®488 (green) and Hoechst 33342 (blue) channels, respectively. White arrows indicate the expression of membrane-bound TNAP on HeLa cells. Scale bar: 25 µm. One representative experiment out of three is shown.

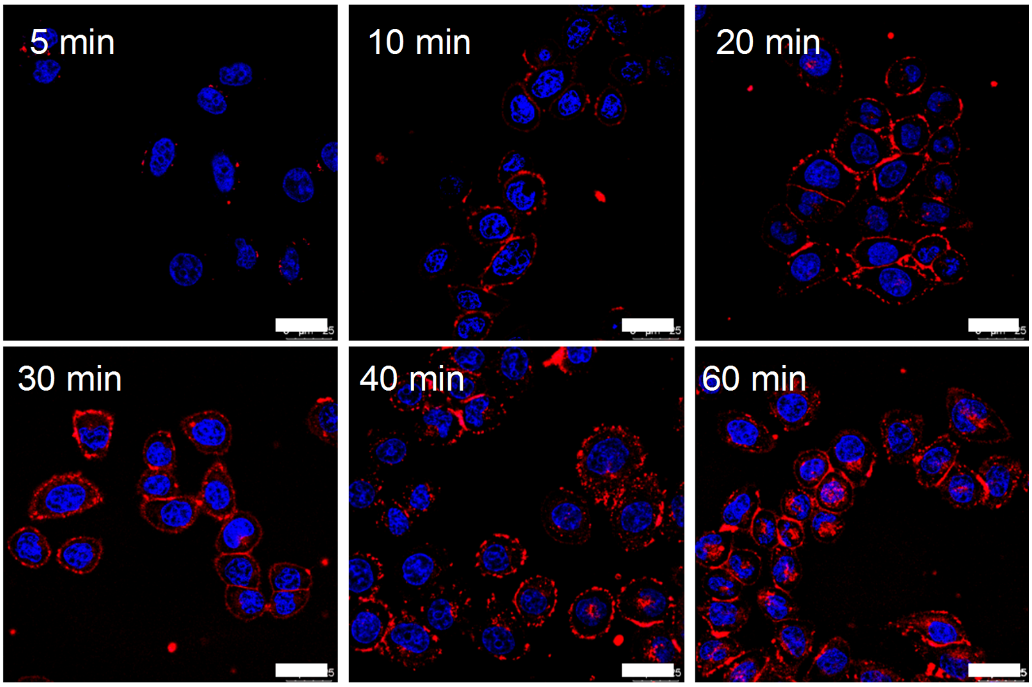

Supplementary Figure 13. NIR FL imaging of HeLa cells upon incubation with P-CyPt (10 µM) for 5-60 min. HeLa cells were incubated with P-CyPt (10 µM) for 5-60 min, and co-stained with Hoechst 33342 (blue). After being washed, the FL images were acquired under NIR channel ($\lambda_{ex/em}$ = 670/750 ± 50 nm) and Hoechst channel ($\lambda_{ex/em}$ = 405/450 ± 20 nm), respectively. NIR FL (red) progressively appeared around cell membrane, and reached the maximum after 30 min. Some punctuate FL appeared intracellularly after 40 min. Scale bars: 25 µm. One representative experiment out of two is shown.

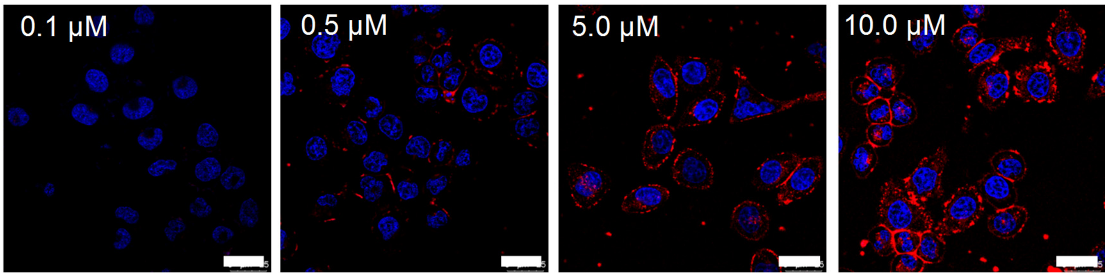

Supplementary Figure 14. NIR FL imaging of HeLa cells following incubation with varying concentration of P-CyPt for 30 min. HeLa cells were incubated with P-CyPt (0.1, 0.5, 5.0, 10.0 µM) for 30 min, and co-stained with Hoechst 33342 (blue). After being washed, the FL images were acquired at NIR channel ($\lambda_{ex/em}$ = 670/750 ± 50 nm) and Hoechst channel ($\lambda_{ex/em}$ = 405/450 ± 20 nm), respectively. NIR FL (red) increased with the concentration of P-CyPt. Scale bars: 25 µm. One representative experiment out of two is shown.

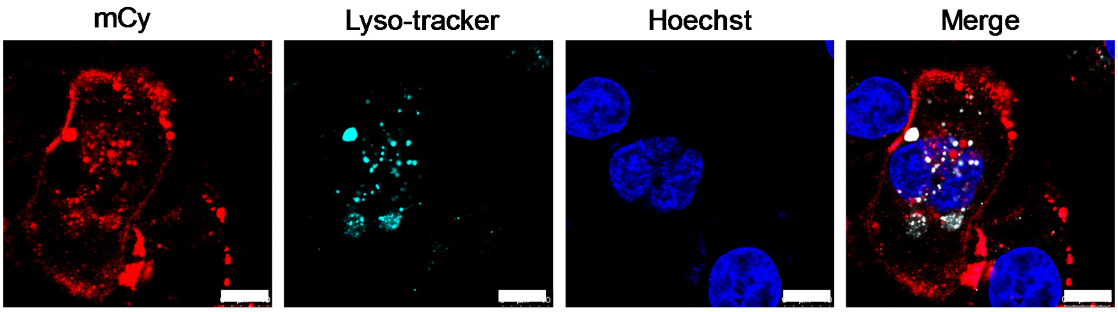

Supplementary Figure 15. Co-localization study of P-CyPt in HeLa cells. HeLa cells were incubated with P-CyPt (10 μM, red) for 1 h, and then stained with the Lyso-tracker (cyan) and Hoechst 33342 (blue). The imaging results showed that the punctate intracellular NIR fluorescence (red) colocalized well with the fluorescence of lyso-tracker, indicating the main distribution of dephosphorylated fluorescent product in the lysosomes in addition to the cell membranes. Scale bars: 10 μm. One representative experiment out of two is shown.

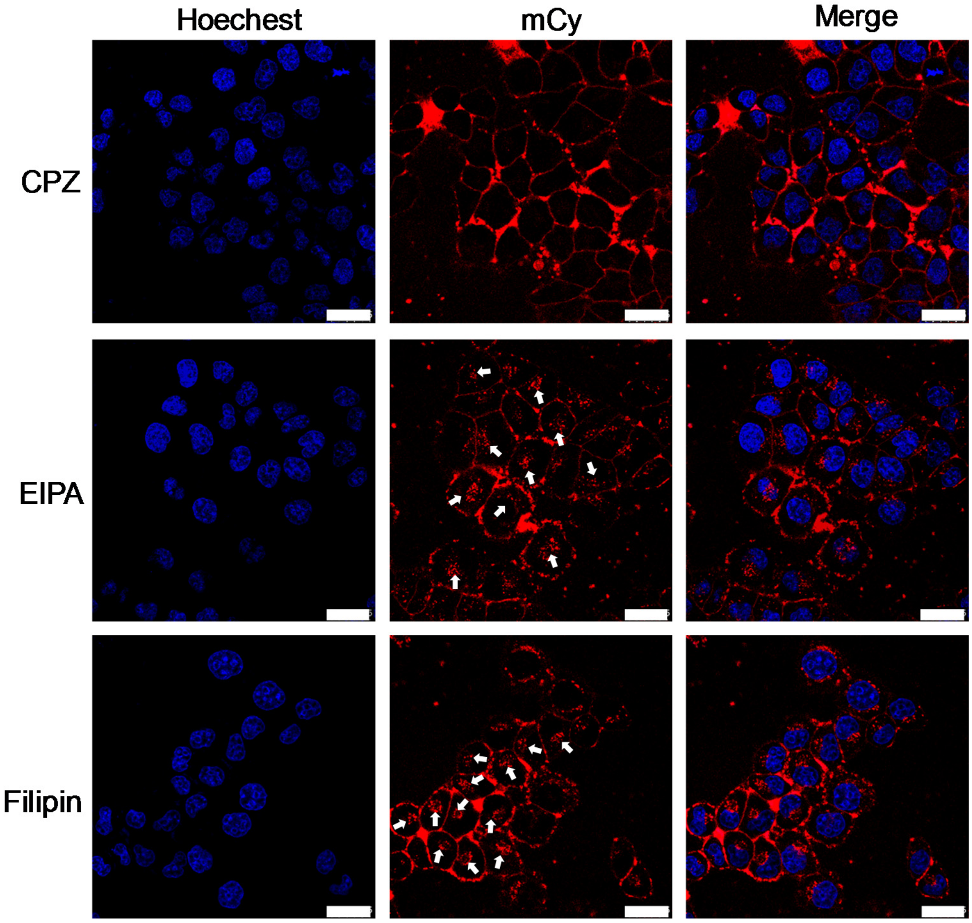

Supplementary Figure 16. Examination of endocytosis pathway in HeLa cells. NIR fluorescence imaging of HeLa cells upon pretreatment with different endocytosis inhibitor, following by incubation with P-CyPt (10 μM) for 60 min. To inhibit endocytosis, HeLa cells were first incubated with chlorpromazine (CPZ, 50 μM),

ethylisopropyl amiloride (EIPA, 100 μM), or filipin III (5 μg/mL) for 30 min, and then incubated with P-CyPt (10 μM) for 60 min. White arrows indicate the appearance of fluorescent punctuates in the interior of HeLa cells. HeLa cells pretreated with CPZ showed reduced intracellular fluorescence, which was not largely inhibited by EIPA or filipin III. These imaging results indicate that the in situ formed Pt^{IV}NPs can enter HeLa cells mainly via the clathrin-dependent endocytosis, not via micropinocytosis (EIPA) or caveolae-mediated endocytosis (filipin III). Scale bars: 25 μm. One representative experiment out of two is shown.

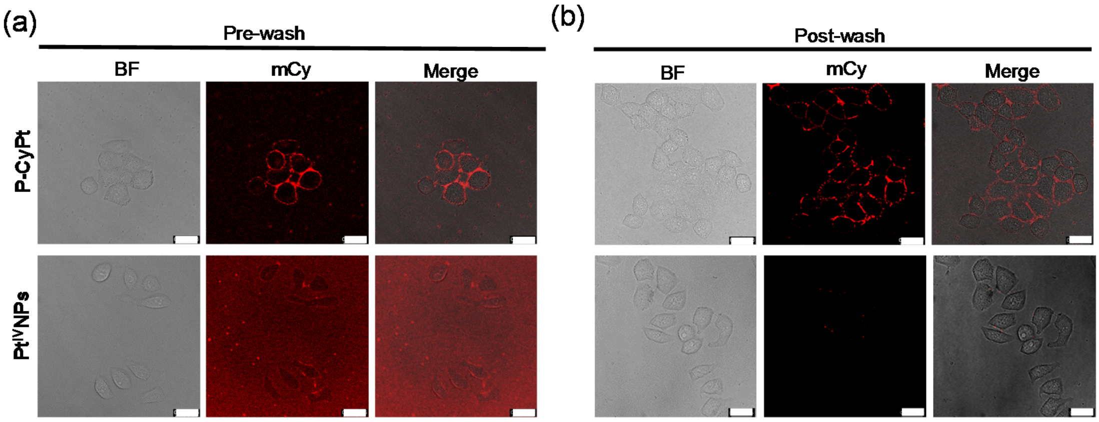

Supplementary Figure 17. Evaluation of the different ability to anchor on cell membranes between in situ formed Pt^{IV}NPs and preformed Pt^{IV}NPs. Confocal fluorescence imaging of HeLa cells following incubation with P-CyPt (10 μM) or preformed Pt^{IV}NPs (10 μM) for 30 min. (a) The images were acquired before wash (Pre-wash). (b) The images were acquired after removal of medium and washed gently with PBS buffer (Post-wash). Scale bars: 25 μm. These results suggest that the ALP-mediated in situ self-assembled Pt^{IV}NPs are prone to adhere on the membranes of HeLa cells, while preformed Pt^{IV}NPs could not adhere on the cell membranes. One representative experiment out of two (a,b) is shown.

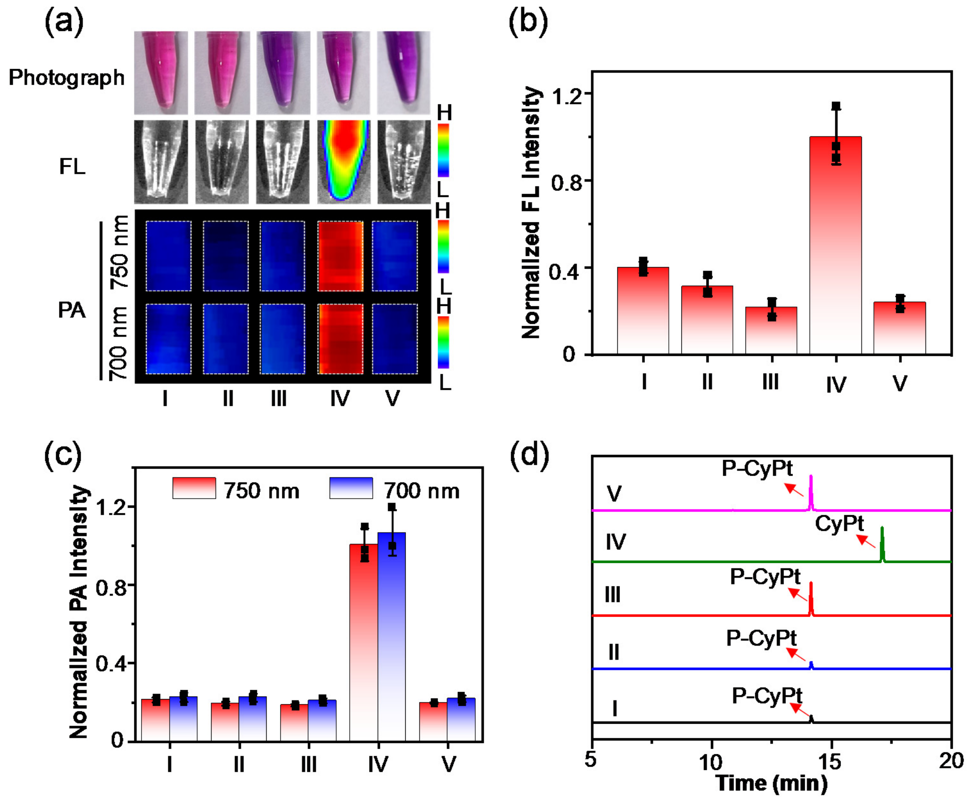

Supplementary Figure 18. NIR FL/PA bimodality imaging of different culture mediums collected from indicated cells. (a) Photograph, FL images, and dual PA images (700&750 nm), (b) normalized NIR FL intensity, (c) normalized dual PA intensities, and (d) HPLC traces (660 nm detection) of different culture mediums. I: HeLa cells incubated with P-CyPt (10 μM) for 30 min; II: HeLa cells incubated with P-CyPt (10 μM) for 30 min, then washed with PBS three times, added blank medium and incubated for another 3 h; III: HeLa cells pretreated with Na_3VO_4 (10 mM) for 20 min, followed by incubating with P-CyPt (10 μM) for another 30 min; IV: HeLa cells incubated with preformed Pt^{IV}NPs (10 μM) for 30 min; V: HEK-293T cells were incubated with P-CyPt (10 μM) for 30 min. Data denote mean ± s.d. (n = 3 independent cell pellets).

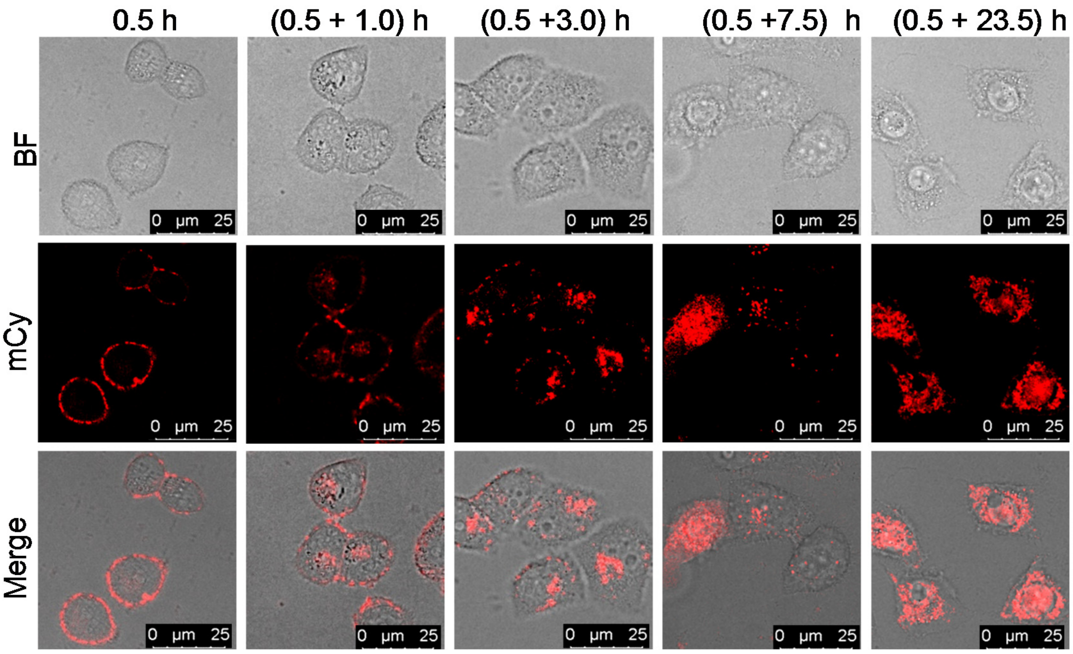

Supplementary Figure 19. Evaluation of time-dependent intracellular translocation of in situ formed Pt^{IV}NPs in HeLa cells. Confocal fluorescence images of HeLa cells incubated with P-CyPt (10 μM) for 30 min, and then washed with PBS for three times, followed by incubation with blank DMEM medium for 1, 3, 7.5 and 23.5 h. Scale bars: 25 μm. The resutls show that NIR FL was first distributed around cell membrane, which was mostly translocated into cells after 3 h. The intracellular NIR fluorescence became much brighter after elongation to 23.5 h, presumably owing to the GSH-triggered disassembly of Pt^{IV}NPs intracellularly. Moreover, bright field (BF) images also showed that a distinct change in cell morphology after 23.5 h. One representative experiment out of two is shown.

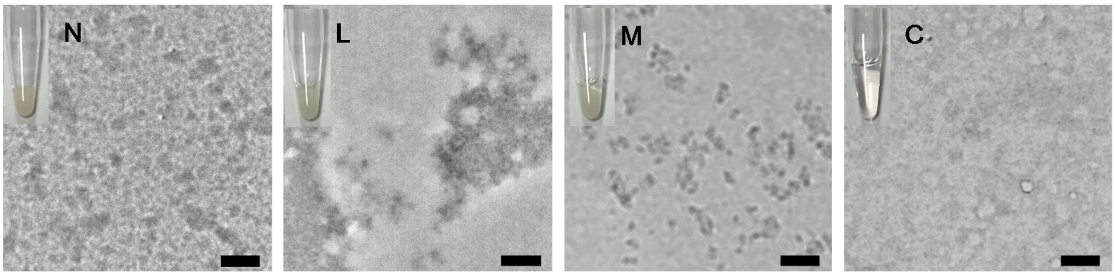

Supplementary Figure 20. TEM images of different fractions of blank HeLa cell lysates. HeLa cells were lyses, and separated via centrifugation under different speed. N: Nuclei; L: lysosomes, peroxisomes mitochondria; M: plasma membrane, microsomal fraction; C: soluble portion of cytoplasm. Inset: Photographs of each cell fraction after being re-suspended in 200 μL of D.I. water. Scale bars: 200 nm. One representative experiment out of two is shown.

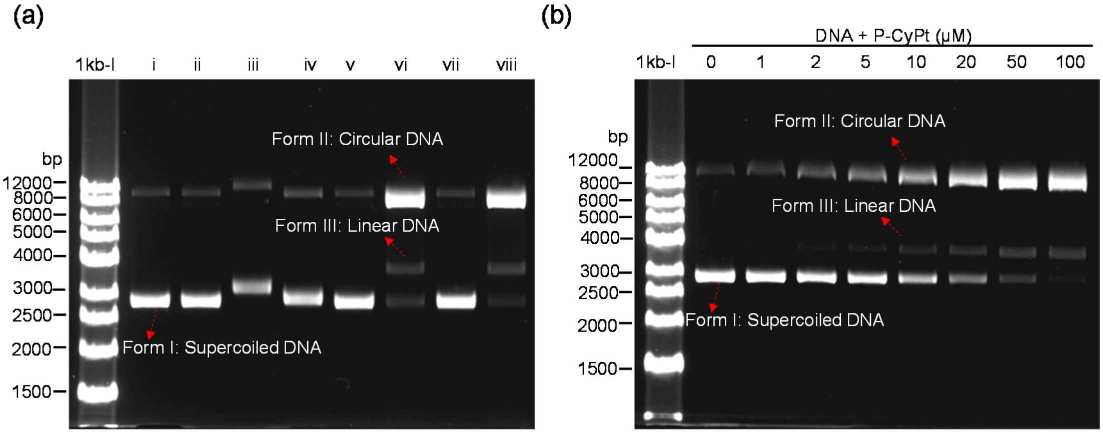

Supplementary Figure 21. Evaluation of the ability to break DNA double strand by different species of Pt compounds. (a) Agarose gel electrophoresis of supercoiled pBR322 plasmid DNA (10 ng/μL) after incubating with indicated conditions. 1kb-I represents the linear DNA marker ladder (500-12000 bp); Lane i: DNA; Lane ii: DNA + GSH (2 mM); Lane iii: DNA + CDDP (100 μM); Lane iv: DNA + CDDP (100 μM) + GSH (2 mM); Lane v: DNA + P-CyPt (100 μM); Lane vi: DNA + P-CyPt (100 μM) + GSH (2 mM), Lane vii: DNA +P-CyPt (100 μM) + ALP (500 U/L), Lane viii: DNA +P-CyPt (100 μM) + ALP (500 U/L) +GSH (2 mM); (b) Agarose gel electrophoresis of supercoiled pBR322 plasmid DNA (20 ng/μL) after incubating with varying concentrations of P-CyPt (0, 1, 2, 5, 10, 20, 50 and 100 μM) respectively) plus ALP (500 U/L), followed by incubation with GSH (2 mM) in Tris buffer at 37 ºC for 16 h. The results in (a) demonstrate that: 1) CDDP itself cannot obviously relax or break the supercoiled DNA (Form I), but can bind to the DNA double strand and retard DNA movement in the gel (iii); 2) the addition of GSH into CDDP can scavenge CDDP and prevent its binding with the DNA double strand (iv); 3) P-CyPt itself cannot bind to and break the DNA double strand (v), but the addition of GSH to reduce Pt(IV) in P-CyPt can significantly relax the supercoiled DNA (Form II: open circular DNA), and partially break the DNA double strand (Form III: linear DNA) (vi); 4) Pt^{IV}NPs that are formed by ALP-triggered dephosphorylation and in situ self-assembly of P-CyPt cannot bind to and break the DNA double strand neither (vii); 5) the addition of GSH to reduce Pt(IV) in the as-formed Pt^{IV}NPs can relax the supercoiled DNA (Form II) and partially break the DNA double strand (Form III) as well (viii). These data suggest that GSH-triggered reduction and release of Pt(II) can efficiently relax the supercoiled DNA and break the DNA double strand, which is highly dependent on the concentration of P-CyPt (b). One representative experiment out of two is shown.

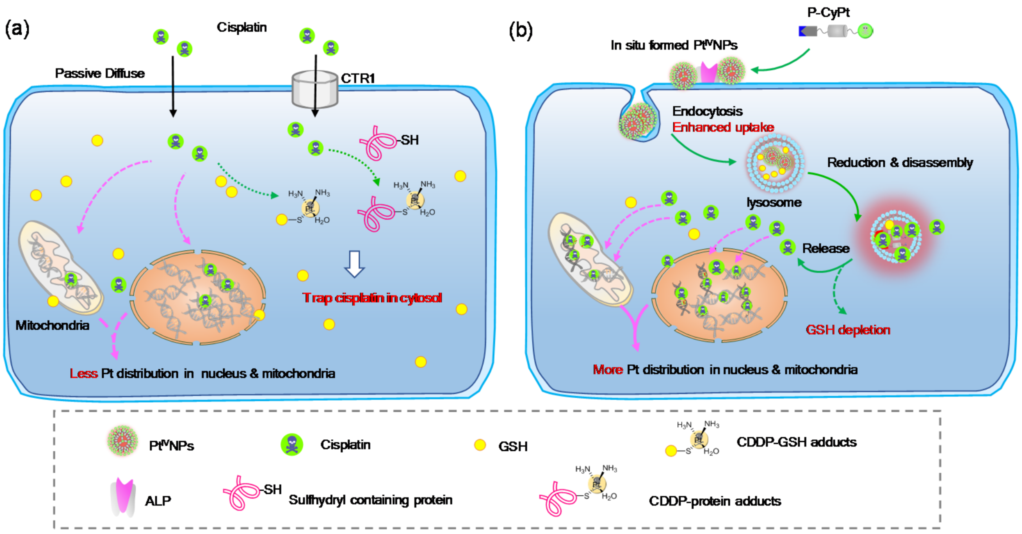

Supplementary Figure 22. Schematic illustration of the mechanism affecting the different uptake and intracellular accumulation of Pt drugs between free cisplatin (a) and P-CyPt (b). As shown in (a), cisplatin is highly polar and enters cells relatively slowly compared to other classes of small-molecule drugs. Though the uptake route of cisplatin into cells is not completely understood, it was reported that cisplatin could enter cells mainly via passive diffusion, and some plasma-membrane transporters (e.g., copper transporter CTR1) mediated active uptake may also be involved (*Nat. Rev. Cancer* **2007**, *7*, 573; *Coord. Chem. Rev.* **2009**, *253*, 2070). Once it enters cells, it is mainly distributed in the cytosol, where it becomes activated via displacing the chloride atoms by water molecules. This hydrolyzed product is a potent electrophile that can react with intracellular nucleophiles, including the reactive sulfhydryl groups in reducing biothiols (e.g., GSH) and proteins in the cytosol. Thus, the cisplatin is mainly captured in the cytosol, and only a few of cisplatin (and its hydrolyzed product) can enter nucleus and react with the nitrogen donor atoms in DNA (*Eur. J. Pharmacol.* **2014**, *740*, 364). Different to cisplatin, cellular uptake of the in situ formed Pt^{IV}NPs is mainly via the clathrin-dependent endocytosis process (b). Once they enter cells, Pt^{IV}NPs first reach the lysosomes, and are then reduced by abundant endogenous GSH, leading to disassembly and burst release of cisplatin in the lysosomes. Some of the released cisplatin can escape into the cytosol. This process could dramatically increase the intracellular concentration of cisplatin, which might outcompete the nucleophiles (e.g., GSH, free sulfhydryl-containing proteins) in the cytosol. Meanwhile, as the intracellular GSH is also depleted accompanying by the disassembly and succeeding release of cisplatin, cisplatin that is trapped by GSH could be reduced in the cytosol. These two effects can allow more cisplatin to diffuse into other organelles (e.g., nucleus and mitochondria). Thus, the distributions of Pt in nucleus, lysosomes and mitochondria

are found to be higher in cells incubated with P-CyPt than cisplatin.

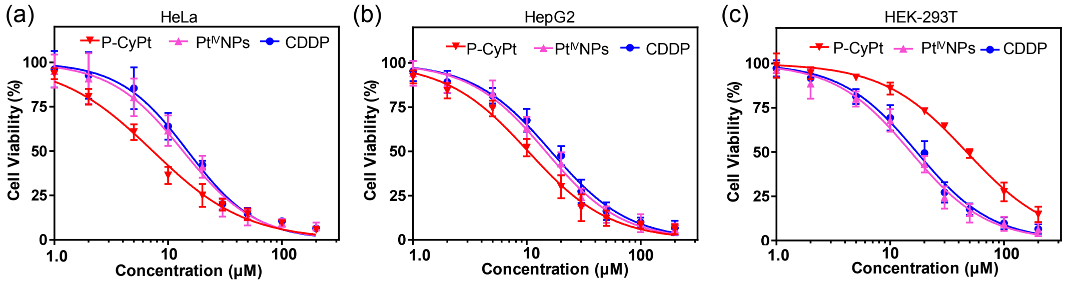

Supplementary Figure 23. Evaluation of cytotoxicity of different Pt drugs. Cell viability of (a) HeLa, (b) HepG2 and (c) HEK-293T cells after being incubated with varying concentrations of P-CyPt, preformed Pt^{IV}NPs or CDDP for 48 h. Date denote mean ± s.d. (n = 3 biologically independent experiments).

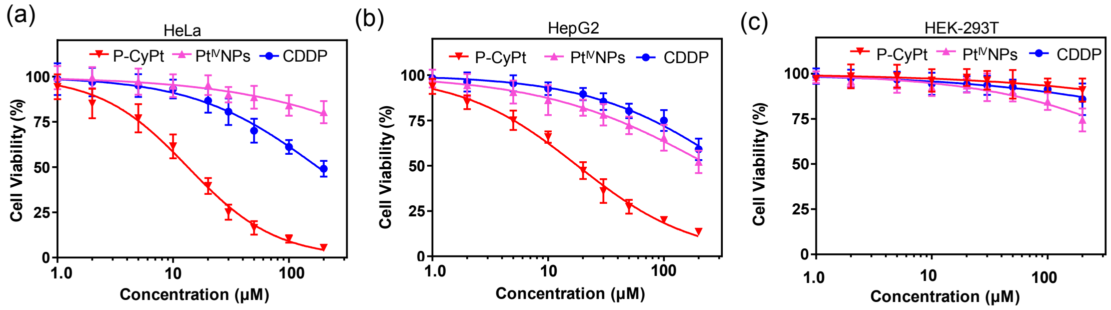

Supplementary Figure 24. Evaluation of cytotoxicity of different Pt drugs. Cell viability of (a) HeLa, (b) HepG2 and (c) HEK293T cells after being incubated with varying concentrations of **P-CyPt,** preformed **Pt^{IV}NPs** or CDDP for 2 h, then washed with cold PBS for three times, and then continue incubation in blank mediums for another 48 h. Date denote mean ± s.d. (n = 3 biologically independent experiments).

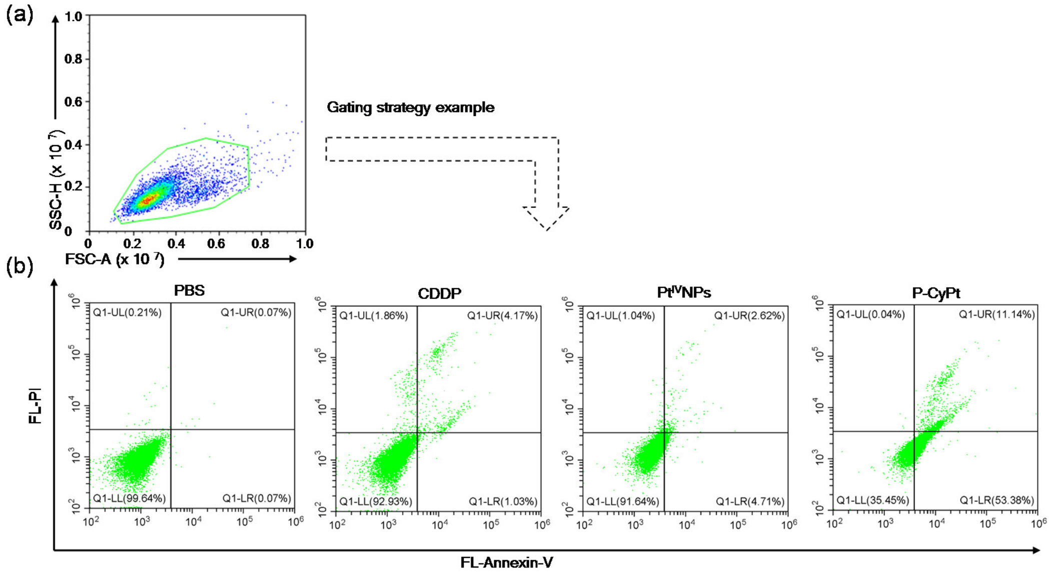

Supplementary Figure 25. Flow cytometric analysis of HeLa cell death. (a) Gating strategy applied for flow cytometric analysis. (b) Representative flow cytometric analysis of HeLa cells by Annexin V-FITC/PI staining. HeLa cells were treated with PBS, CDDP, Pt^{IV}NPs or P-CyPt (20 μM) for 2 h, washed with cold PBS for three times, and then continued incubation in refresh blank DMEM medium for another 48 h. The cells were then stained with the Annexin V-FITC/PI kit, and analyzed by flow cytometry. One representative experiment out of two is shown.

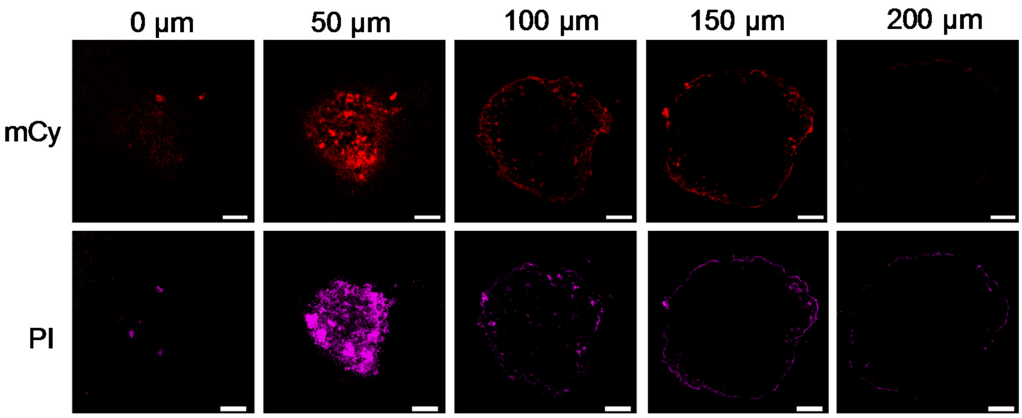

Supplementary Figure 26. Evaluation of the penetration and cytotoxicity of Pt^{IV}NPs toward HeLa MCTS. MCTS were incubated with Pt^{IV}NPs (10 μM) for 1 h, then washed with PBS buffer for three times, followed by incubation for another 24 h. The MCTs were then co-stained with PI. The FL images at mCy ($\lambda_{ex/em}$ = 670/710 nm, red) and PI channel ($\lambda_{ex/em}$ = 550/640 nm, magenta) were acquired using a confocal laser scanning microscopy. Scale bars: 100 μm. One representative experiment out of two is shown. These imaging results demonstrate that preformed Pt^{IV}NPs hold a reduced ability to penetrate and induce cell death in HeLa MCTS compared to that of the small-molecule P-CyPt.

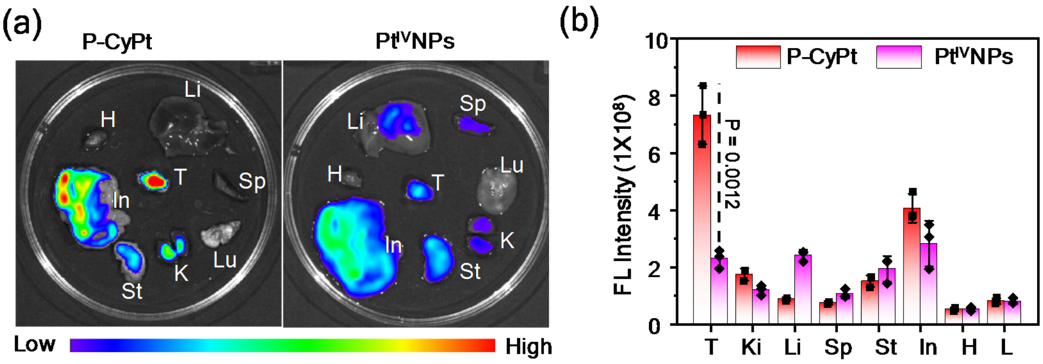

Supplementary Figure 27. Ex vivo FL imaging of tumors and main organs. (a) Representative ex vivo FL images ($\lambda_{ex/em}$ = 660/750 ± 50 nm) and (b) average FL intensities of tumors and main organs resected from P-CyPt- or Pt^{IV}NPs-treated HeLa tumor. HeLa tumor-bearing mice were i.v. injected with P-CyPt or Pt^{IV}NPs (100 μM, 200 μL). After 4 h, the mice were sacrificed. Tumors (T) and main organs, including liver (L), heart (H), spleen (Sp), lung (Lu), kidney (K), stomach (St), and intestine (In) were resected, and the FL images were acquired on the IVIS spectrum imager. Values denote mean ± s.d. (n = 3 independent animals). Statistical difference was analyzed by Student's two-sided t-test. Source data are provided as a Source Data file.

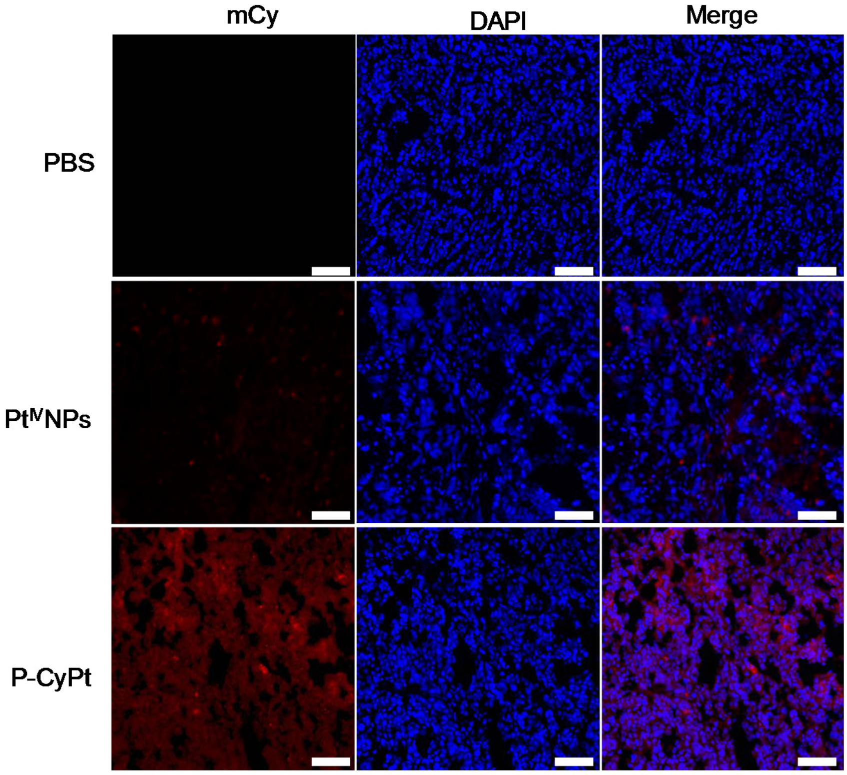

Supplementary Figure 28. FL imaging of resected tumor tissue slices. FL images of tumor tissue slices (10 μm thickness) resected from s.c. HeLa tumor-bearing mice at 4 h after i.v. injection of PBS (200 μL), Pt^{IV}NPs or P-CyPt (100 μM, 200 μL). Cell nuclei were co-stained with DAPI (blue). Scale bars: 100 μm. One representative experiment out of two is shown.

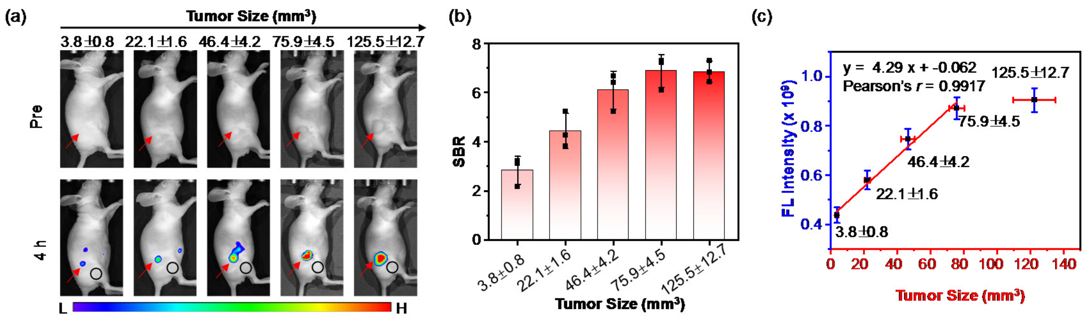

Supplementary Figure 29. Fluorescence imaging of varying sizes of s.c. HeLa tumors in mice. (a) Representative fluorescence images of mice with s.c. HeLa tumors at a mean size of 3.8 ± 0.8, 22.1 ± 1.6, 46.4 ± 4.2, 75.9 ± 4.5 and 125.5 ± 12.7 mm^3, respectively. P-CyPt (100 µM, 200 µL) was i.v. injected into mice, and the whole-body fluorescence images were acquired at 0 and 4 h. Red arrows indicate the locations of tumors, and the black circle indicates the position chosen for the background in each mouse. (b) Calculated SBR in mice of different tumor sizes. (c) Plotting of the tumor FL intensity versus the tumor size reveals that the mean tumor fluorescence intensity linearly correlated with the mean tumor size ranging from 3.8 ± 0.8 mm^3 to 75.9 ± 4.5 mm^3 (Person's r = 0.9917). Data denote mean ± s.d. (n = 3 independent animals). These results suggest that P-CyPt can successfully detect small HeLa tumor in the early stages.

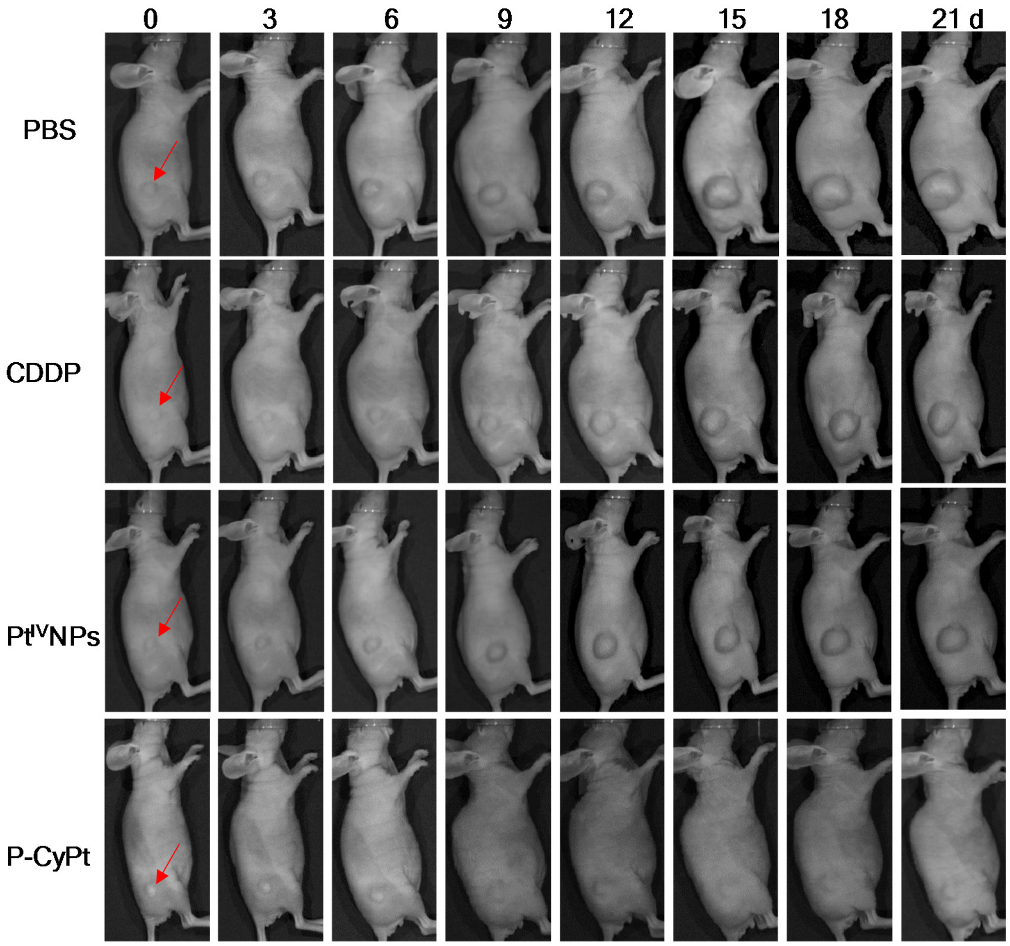

Supplementary Figure 30. Representative photographs of s.c. HeLa tumor implanted mice following indicated treatment. The s.c. HeLa tumor-bearing mice were i.v. injected with PBS, CDDP, P-CyPt or Pt^{IV}NPs (2.25 mg kg^{-1} Pt, 200 μL) at day 0, 3, 6, 9 and 12, and monitored for over 21 days. The images were acquired using the IVIS Lumina XR III imaging system every three days. Red arrows indicate the tumors location in each mouse.

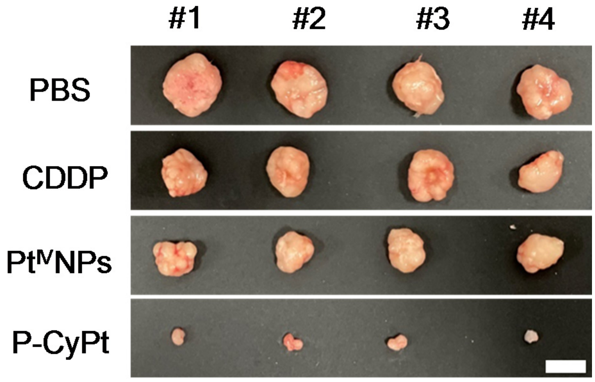

Supplementary Figure 31. Photographs of HeLa tumors resected from each mouse after indicated treatment. HeLa tumor-bearing mice were treated with PBS, CDDP, P-CyPt or Pt^{IV}NPs (2.25 mg kg^{-1} Pt, 200 μL) at day 0, 3, 6, 9 and 12. On day 21, all the mice were sacrificed, and the tumors were resected. Scale bar: 1 cm.

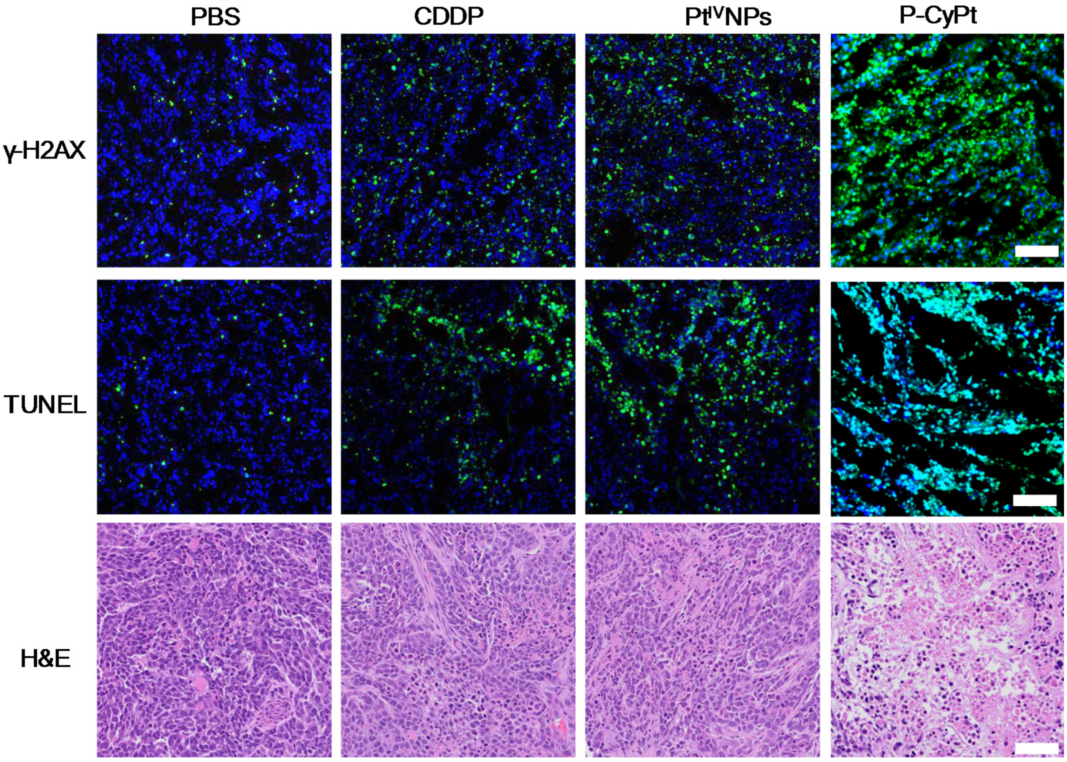

Supplementary Figure 32. Examination of tumor cell death in s.c. HeLa tumor-bearing mice following indicated treatment. Phosphorylated histone H2AX (γH2AX, up), Terminal deoxynucleotidyl transferase dUTP nick end labeling (TUNEL, middle) and Hematoxylin-eosin (H&E, bottom) staining of tumor tissue slices resected from mice at 2 days following i.v. injection of PBS (200 μL), CDDP, P-CyPt or Pt^{IV}NPs (2.25 mg kg^{-1} Pt, 200 μL). Cell nuclei were stained with DAPI (blue). The FL images of γH2AX (Green) and TUNEL (Green) staining were both acquired under a FITC filter ($\lambda_{ex/em}$ = 495/540 ± 20 nm). Scale bars: 100 μm. One representative experiment out of two is shown.

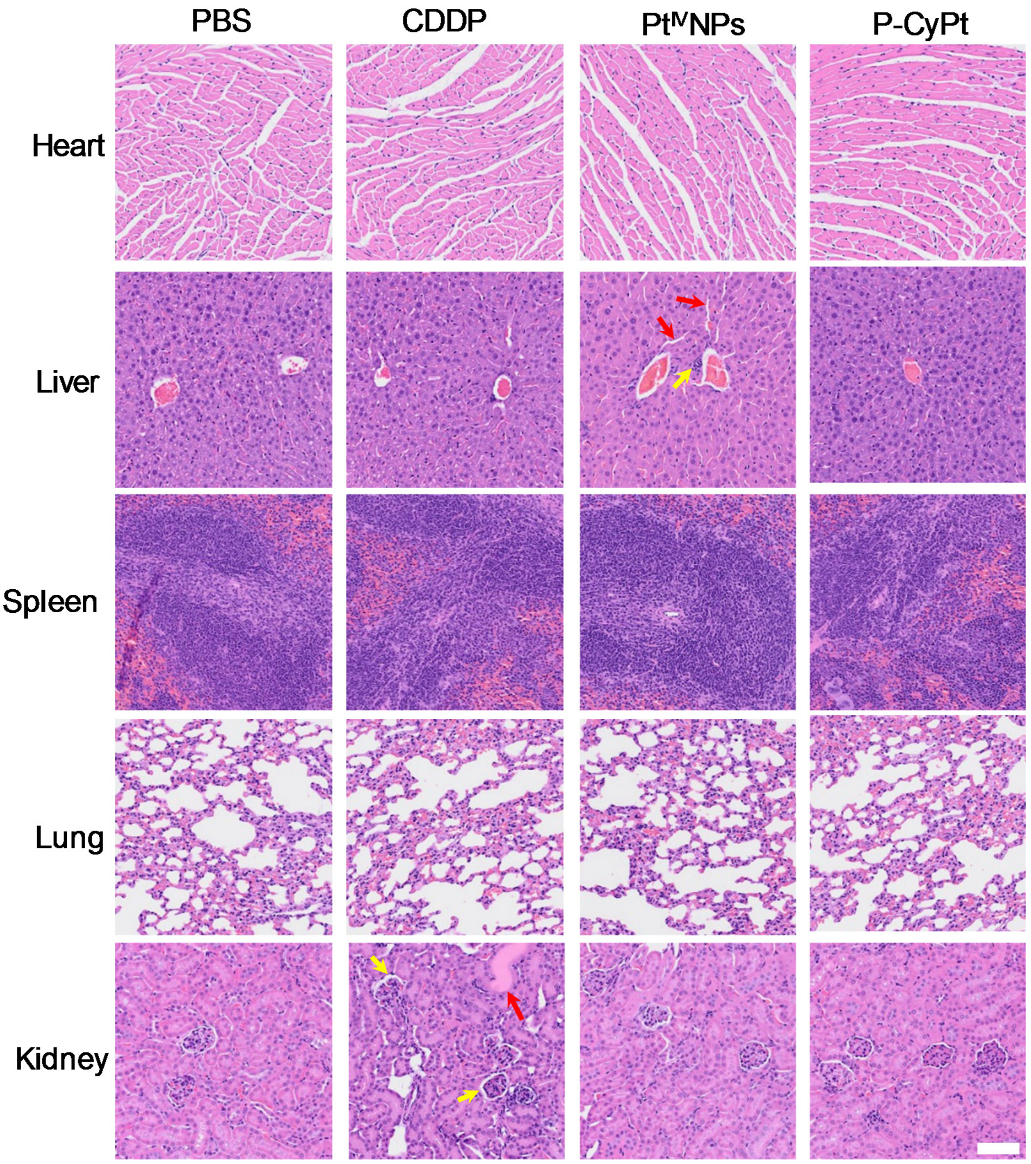

Supplementary Figure 33. Examination of the potential in vivo side-toxicity of different Pt drugs. Representative H&E images of major organ tissue slices resected from mice at day 21 post indicated treatments. Mice with s.c. HeLa tumors were i.v. injected with PBS (200 µL), CDDP (2.25 mg kg^{-1} Pt, 200 µL), Pt^{IV}NPs (2.25 mg kg^{-1} Pt, 200 µL) or P-CyPt (2.25 mg kg^{-1} Pt, 200 µL) at day 0, 3, 6, ,9 and 12. Then, all mice were kept feeding for another 9 days. On day 21, the mice were sacrificed. The main organs, including heart, liver, kidneys, spleen and lung were resected, cut into 10-µm slices, and applied for H&E staining. Red arrows indicate the damage regions occurred in the liver of Pt^{IV}NPs-treated mice and in the kidneys of CDDP-treated mice, respectively. In CDDP-treated group, we observed cast formation (red arrow) and widened Bowman's spaces (yellow arrows), implying the occurrence of nephrotoxicity in mice after treatment with CDDP. In Pt^{IV}NPs-treated group mice, we observed decreased nucleus, dilation sinusoids (red arrows) and inflammatory cell infiltration (yellow arrow) in the liver tissue, suggesting the occurrence of hepatotoxicity in mice

following continuous treatment with Pt^{IV}NPs. In contrast, all organs looked normal between PBS- and P-CyPt-treated mice. These data demonstrate that P-CyPt holds low side-toxicity compared with CDDP or Pt^{IV}NPs. Scale bar: 100 μm. The occurrence of hepatotoxicity in Pt^{IV}NPs-treated mice could be presumably owing to (1) the large size of Pt^{IV}NPs (~160 nm) that caused a high liver uptake and (2) the fast release of cytotoxic cisplatin from the liver-resided Pt^{IV}NPs by hepatic GSH that led to a high concentration of cisplatin in the liver. One representative experiment out of two is shown.

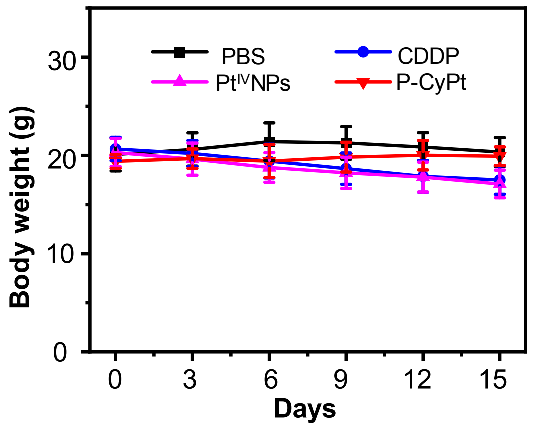

Supplementary Figure 34. Monitoring of the body weight of mice with orthotopic HepG2/Luc liver tumors following indicated treatments. The orthotopic HepG2/Luc liver tumor-bearing mice were i.v. injected with PBS (200 μL), CDDP, Pt^{IV}NPs or P-CyPt (2.25 mg kg^{-1} Pt, 200μL) on day 0, 3, 6, 9 and 12. Values denote mean ± s.d. (n = 4 independent animals).

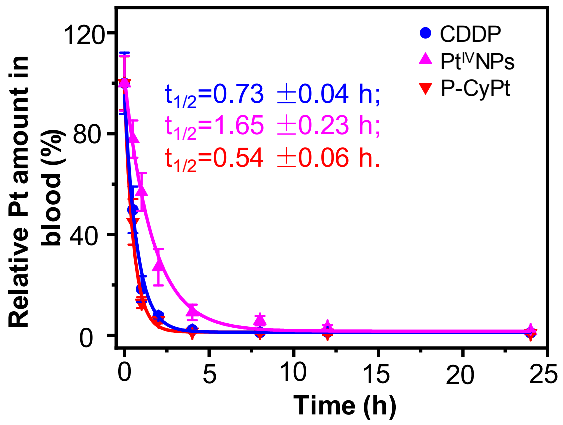

Supplementary Figure 35. Examination of the blood circulation half-lives of P-CyPt, Pt^{IV}NPs, and CDDP. Plots of the relative amount of Pt (%) in the blood after i.v. injection of P-CyPt, Pt^{IV}NPs, and CDDP (at a dose of 2.25 mg kg^{-1} Pt) into healthy mice. Data are mean ± s.d. (n = 3 independent animals). The results revealed that P-CyPt as a hydrophilic small-molecule probe holds a similar blood circulation half-life ($t_{1/2}$ = 0.54 ± 0.06 h) to CDDP (0.73 ± 0.04 h), which was shorter than Pt^{IV}NPs (1.65 ± 0.23 h).

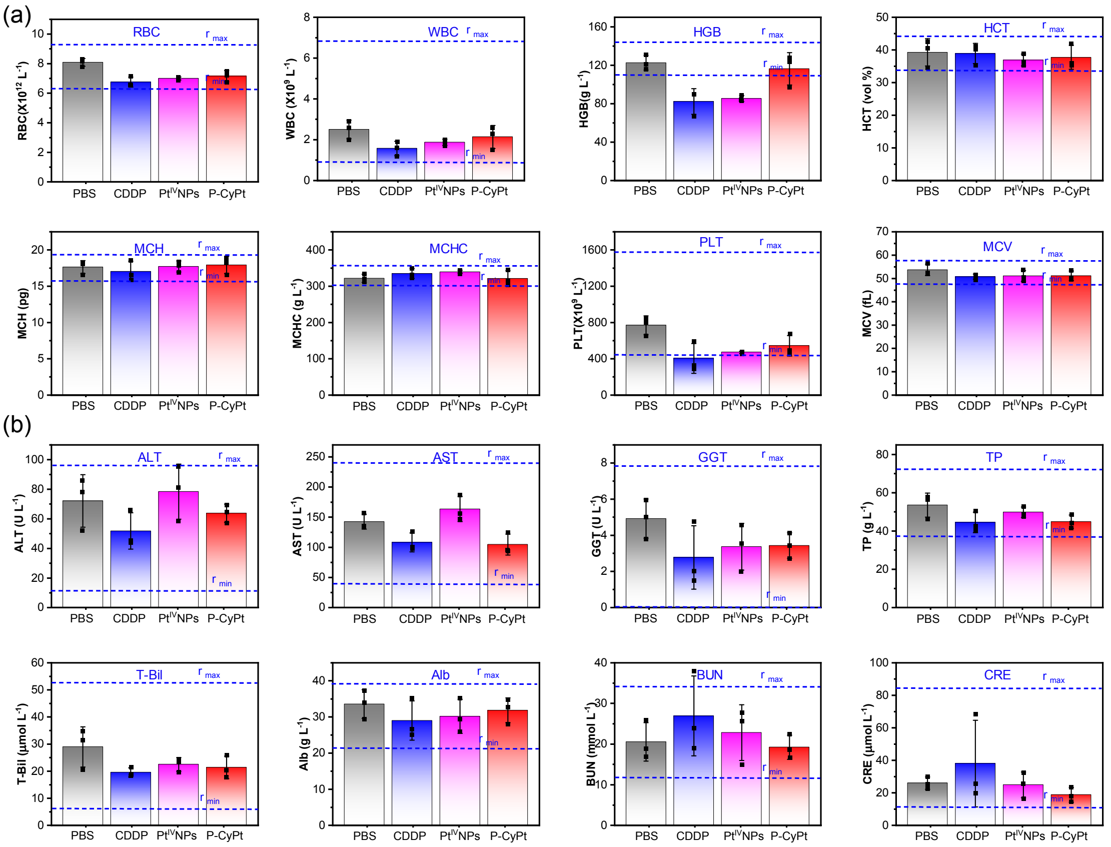

Supplementary Figure 36. Examination of the biosafety of different Pt drugs in healthy mice. (a) Blood count and (b) Biochemistry analysis of nude mice at 1-day post i.v. injection of PBS (200 μL), CDDP, P-CyPt or Pt^{IV}NPs (2.25 mg kg^{-1} Pt). RBC: red blood cells; WBC: white blood cells; HGB: hemoglobin; HCT: hematocrit; MCH: mean corpuscular hemoglobin; MCHC: mean corpuscular hemoglobin concentration; PLT: platelet count; MCV: mean corpuscular volume; ALT: alanine aminotransferase; AST: glutamic oxaloacetic transaminase; GGT: γ-glutamyl transpeptidase; TBil: total bilirubin; Alb: albumin; TP: total protein; BUN: blood urea nitrogen; CRE: creatinine. Blue short dotted lines indicate the range of normal reference values (r_{min} → r_{max}) for BALB/c mice. These data show that all the values were within the normal reference for P-CyPt, suggesting high biosafety. Values denote mean ± s.d. (n = 3 independent animals).

2. Supplementary Tables 1-13

Supplementary Table 1. ICP-OES analysis of Pt in HeLa cells. [a]

Pt drugs	Pt element per cell (fmol/cell) [b]	Percentage of Pt element taken up by cells (%) [c]
P-CyPt	24.60 ± 3.5	36.9 ± 5.3
Pt^{IV}NPs	1.73 ± 0.2	2.6 ± 0.3
CDDP	0.73 ± 0.09	1.1 ± 0.1
P-CyPt + Na_3VO_4	0.07 ± 0.01	0.1 ± 0.01

[a] HeLa cells were untreated or pre-treated with Na_3VO_4 (10 mM) for 20 min and then incubated with indicated Pt drug (10 μM) for 1 h. Then, the cells were trypsinized, collected, and the cell numbers were counted and digested with trace metal concentrated HNO_3 (65~70%) and HCl (36~38%). The amount of Pt element was determined by ICP-OES.

[b] Pt per cell represents the average amount of Pt element in one HeLa cell, which was calculated by dividing the total amount of Pt element in cells by the cell number.

[c] Percentage of Pt taken up by cells was calculated by dividing the total amount of Pt elements in cells by the amount of Pt drugs added. Values denote mean ± s.d. (n = 3 independent experiments).

Supplementary Table 2. Comparison of the intracellular GSH in HeLa cells before and after incubation with different Pt drugs. [a]

Pt drugs	[GSH] before treatment (fmol/cell)	[GSH] after treatment (fmol/cell)	[GSH] depletion [b]
CDDP		11.81 ± 1.7	8.3%
Pt^{IV}NPs	12.88 ± 1.5	11.71 ± 1.6	9.1%
P-CyPt		8.07 ± 0.8	37.3%

[a] HeLA cells were untreated or treated with different Pt drugs for 2 h. The cells were washed with PBS for three times, and replaced with fresh medium and incubated for another 24 h. After that, the cells were trypsinized, centrifuged and counted. The cell pellets were dispersed, lysed via sonication, and ultracentrifuged to obtain supernatant. The GSH content (n_{Pt}) in the supernatant was measured by a reported detection method. The content of GSH per cell ([GSH]) before and after treatment (n_B and n_A) was calculated according to the formula: n_{Pt}/N_{Pt}.

[b] GSH depletion: The GSH depletion (Δ) was calculated according to the formula: $\%\Delta = (n_B - n_A)/n_B$. Values denote mean ± s.d. (n = 3 independent experiments).

Supplementary Table 3. Comparison of the distribution of Pt element in different subcellular organelles between P-CyPt and CDDP. [a]

Subcellular organelles	Amount of Pt distributed in a single cell's organelle (fmol) [b]		Percentage of Pt distributed in a single cell's organelle (%) [c]	
	P-CyPt	CDDP	P-CyPt	CDDP
Nucleus	7.05 ± 0.90	0.0044 ± 0.00045	29.05%	~5.65%
Lysosome& Mitochondria	6.34 ± 0.81	0.0032 ± 0.00033	26.11%	~4.11%
Membrane	1.46 ± 0.19	0.011 ± 0.0011	6.02%	~14.02%
Cytoplasm	9.42 ± 1.20	0.059 ± 0.0061	38.82%	~76.22%

[a] HeLa cells were incubated with **P-CyPt** or CDDP (10 μM) for 1 h. After that, the cells were washed with PBS for three times, replaced with blank medium and cultured for another 24 h. The cells were trypsinized, collected, and the cell numbers were counted. The cell pellets were dispersed in PBS buffer, lysed via ultrasound sonication, and centrifuged at different speed to separate membrane, nucleus, lysosome together with mitochondria, and cytoplasm. The concentration of Pt element in each organelle fraction was then measured by ICP-OES. Values denote mean ± s.d. (n = 3 independent experiments).

[b] The amount of Pt in a single HeLa cell's subcellular organelle was calculated by dividing the total amount of Pt element in different subcellular organelles to the cell number.

[c] Percentage of Pt distributed in a single HeLa cell's subcellular organelles was calculated by dividing the amount of Pt in a single cell's subcellular organelles by the amount of Pt in a single cell (sum of organelles).

Supplementary Table 4. The IC_{50} (μM) value of different Pt drugs toward HeLa, HepG2 and HEK-293T cells.[a]

Compounds	Condition	IC_{50} (μM)		
		HeLa	HepG2	HEK-293T
P-CyPt	48 h [b]	6.76 ± 0.68	10.27 ± 0.87	48.60 ± 4.24
	48 h + 2 h [c]	14.56 ± 1.24	18.12 ± 1.78	>200
Pt^{IV}NPs	48 h [b]	13.52 ± 1.02	14.64 ± 0.74	15.19 ± 1.44
	48 h + 2 h [c]	>200	>200	>200
CDDP	48 h [b]	14.47 ± 1.60	16.3 ± 1.69	17.41 ± 1.05
	48 h + 2 h [c]	177.78 ± 16.57	>200	>200

[a] The IC_{50} values were measured by the MTT assay. Values denote mean ± s.d. (n = 3 independent experiments).
[b] Cells were incubated with varying concentrations of CDDP, P-CyPt or Pt^{IV}NPs for 48 h.
[c] Cells were incubated with varying concentrations of CDDP, P-CyPt or Pt^{IV}NPs for 2 h, and the mediums were removed, washed with PBS. Blank culture mediums were added, and the cells were kept incubation for another 48 h.

Supplementary Table 5. Percentages of P-CyPt and its metabolites that are excreted to the urine.[a]

Time	P-CyPt (%)	CyPt (%)	Cy-COOH (%)
0-2 h	11.29 ± 1.23	1.03 ± 0.11	0.44 ± 0.048
2-4 h	5.12 ± 0.51	0.56 ± 0.055	6.76 ± 0.67
4-8 h	4.33 ± 0.055	0.27 ± 0.23	7.95 ± 0.67
8-12 h	1.41 ± 0.23	0	5.60 ± 0.92

[a] **P-CyPt** (2.25 mg kg^{-1} Pt) was i.v. injected into mice, and the urine was collected at 0-2 h, 2-4 h, 4-8 h and 8-12 h, respectively. P-CyPt and its metabolites in the urine were quantified by analytical HPLC (detection at 660 nm). Percentages (%) of P-CyPt, CyPt and Cy-COOH were calculated by dividing the amount (mol) of each compound in the urine to the amount (mol) of P-CyPt injected. Values denote mean ± s.d. (n = 3 independent experiments).

Supplementary Table 6. Percentages of P-CyPt and its metabolites that are excreted to the faeces.[a]

Time	P-CyPt (%)	CyPt (%)	Cy-COOH (%)
0-2 h	0.45 ± 0.15	0.036 ± 0.012	0.043 ± 0.015
2-4 h	4.54± 1.05	0.94 ± 0.22	0.55 ± 0.13
4-8 h	0.53 ± 0.080	9.67 ± 1.46	11.09 ± 1.68
8-12 h	0.059 ± 0.011	0.76 ± 0.14	8.38 ± 1.52

[a] **P-CyPt** (2.25 mg kg^{-1} Pt) was i.v. injected into mice, and the faeces were collected at 0-2 h, 2-4 h, 4-8 h and 8-12 h, respectively. P-CyPt and its metabolites in the faeces were quantified by analytical HPLC (detection at 660 nm). Percentages (%) of P-CyPt, CyPt and Cy-COOH were calculated by dividing the amount (mol) of each compound in the faeces to the amount (mol) of P-CyPt injected. Values denote mean ± s.d. (n = 3 independent experiments).

Supplementary Table 7. Percentages of CyPt and its metabolites that are excreted to the urine of mice following i.v. injection of Pt^{IV}NPs.[a]

Time	CyPt (%)	Cy-COOH (%)
0-2 h	0	2.56 ± 0.76
2-4 h	2.06 ± 0.64	5.27 ± 1.64
4-8 h	2.92 ± 0.80	6.54 ± 1.80
8-12 h (2.25 mg kg^{-1} Pt)	1.16 ± 0.43	1.84 ± 0.68

[a] Preformed Pt^{IV}NPs (2.25 mg kg^{-1} Pt) were i.v. injected into mice, and the urine was collected at 0-2 h, 2-4 h, 4-8 h and 8-12 h, respectively. CyPt and its metabolites and its metabolites in the urine were quantified by analytical HPLC (detection at 660 nm). Percentages (%) of CyPt and Cy-COOH were calculated by dividing the amount (mol) of each compound in the urine to the amount (mol) of Pt^{IV}NPs injected. Values denote mean ± s.d. (n = 3 independent experiments).

Supplementary Table 8. Percentages of CyPt and its metabolites that are excreted to the faeces of mice following i.v. injection of Pt^{IV}NPs.[a]

Time	CyPt (%)	Cy-COOH (%)
0-2 h	0.38 ± 0.32	0.30 ± 0.25
2-4 h	11.44 ± 1.67	11.09 ± 1.62
4-8 h	10.98 ± 1.53	23.75 ± 3.31
8-12 h	1.99 ± 0.71	6.00 ± 2.13

[a] Preformed Pt^{IV}NPs (2.25 mg kg^{-1} Pt) were i.v. injected into mice, and the faeces were collected at 0-2 h, 2-4 h, 4-8 h and 8-12 h, respectively. CyPt and its metabolites and its metabolites in the faeces were quantified by analytical HPLC (detection at 660 nm). Percentages (%) of CyPt and Cy-COOH were calculated by dividing the amount (mol) of each compound in the faeces to the amount (mol) of Pt^{IV}NPs injected. Values denote mean ± s.d. (n = 3 independent experiments).

Supplementary Table 9. Percentage of Pt element in the urine.[a]

Time	Percentage of Pt element excreted to the urine (%)		
	P-CyPt (%)	Pt^{IV}NPs (%)	CDDP (%)
0-2 h	15.78 ± 1.76	2.12 ± 0.65	15.46 ± 2.49
2-4 h	13.38 ± 1.45	5.47 ± 1.19	10.94 ± 1.14
4-8 h	9.24 ± 1.49	4.06 ± 0.51	5.45 ± 0.94
8-12 h	3.41 ± 0.84	0.96 ± 0.36	3.48 ± 0.58

[a] Mice were i.v. injected with P-CyPt, Pt^{IV}NPs or CDDP (at a dose of 2.25 mg kg^{-1} Pt), and the urine was collected at 0-2 h, 2-4 h, 4-8 h and 8-12 h, respectively. The amount of Pt element in the urine was measured by ICP-OES. The percentage of Pt element at each sample was calculated by dividing the amount of Pt element to the total amount of Pt drugs injected. Values denote mean ± s.d. (n = 3 independent experiments).

Supplementary Table 10. Percentage of Pt element in the faeces.[a]

Time	Percentage of Pt element excreted to the faeces (%)		
	P-CyPt	CyPt	CDDP
0-2 h	0.38 ± 0.090	0.63 ± 0.21	0.19 ± 0.046
2-4 h	5.92 ± 0.64	16.51 ± 1.34	2.60 ± 0.45
4-8 h	16.94 ± 1.87	21.52 ± 1.73	4.55 ± 0.60
8-12 h	4.40 ± 0.52	3.69 ± 0.77	2.70 ± 0.44

[a] Mice were i.v. injected with P-CyPt, Pt^{IV}NPs or CDDP (at a dose of 2.25 mg kg^{-1} Pt), and the faeces were collected at 0-2 h, 2-4 h, 4-8 h and 8-12 h, respectively. The amount of Pt element in the faeces was measured by ICP-OES. The percentage of Pt element at each sample was calculated by dividing the amount of Pt element to the total amount of Pt drugs injected. Values denote mean ± s.d. (n = 3 independent experiments).

Supplementary Table 11. HPLC condition for the purification of compounds **3**, Cy-Pt and Cy-COOH. [a]

Time (min)	Flow (mL/min)	H_2O (1‰ TFA)	CH_3CN (1‰ TFA)
0	12	65	35
2	12	65	35
20	12	5	95
25	12	0	100
30	12	65	65

[a] Semi-preparative HPLC was operated using a reversed-phase C18 column (Phenomenex, 00G-4252-P0-AX, 21.2 mm × 250 mm).

Supplementary Table 12. HPLC condition for the purification of compounds **4**, **5** and P-CyPt.[a]

Time (min)	Flow (mL/min)	H_2O (1‰ TFA)	CH_3CN (1‰ TFA)
0	12	75	25
2	12	75	25
20	12	25	75
25	12	0	100
30	12	75	25

[a] Semi-preparative HPLC was operated using the reversed-phase C18 column (Phenomenex, 00G-4252-P0-AX, 21.2 mm × 250 mm).

Supplementary Table 13. HPLC condition for the analysis of P-CyPt and other compounds.[a]

Time (min)	Flow (mL/min)	H_2O (1‰ TFA)	CH_3CN (1‰ TFA)
0	1.0	95	5
2	1.0	95	5
20	1.0	5	95
25	1.0	0	100
30	1.0	95	5

[a] Analytical HPLC was operated on a reversed-phase C18 column (Phenomenex, 00G-4252-E0, 4.6 mm × 250 mm).

3. Supplementary Materials and Instruments

Materials. Materials were purchased from qualified reagent supplies with analytical reagent grade and used without further purification. Alkaline phosphatase from bovine intestinal mucosa, sodium orthovanadate (Na_3VO_4), *D*-Luciferin, *N*-ethylmaleimide, cisplatin, *N*-ethylmaleimide, γ-glutamyl transferase (GGT), succinic anhydride, commercial GSH detection kit and Cathepsin B were purchased from Sigma-Aldrich. *N*-Fmoc-*L*-phenylalanine was purchased from GL Biochem (Shanghai) Ltd. Lyso-Tracker@Green DND-26, MMP-2, Caspase-3/7 and Trypsin were obtained from Thermo Fisher Scientific Inc. 3-(4,5-Dimethylthiazol-2-yl)-2,5-diphenyltetrazolium bromide (MTT) kit, propidium iodide (PI), Hoechst 33342 and Annexin V-FITC/PI apoptosis staining kit were obtained from KeyGen Biotech. Co. Ltd. (Nanjing, China). Supercoiled pBR322 plasmid DNA (pBR322 Dna Ea, SD0041) was purchased from the Thermo Scientific, 5x loading buffer (5x loading buffer, GR0205-500) and 1kb-I DNA ladder (1kb-I DNA ladder, GsDL10001) were purchased from the General Biotech.

Instruments and Software. Flash chromatography was performed on 40-63 μm silica gel. ^{1}H-, ^{13}C- and ^{195}Pt-NMR spectra were obtained on Bruker DRX-400 spectrometer at 298 K, and analyzed using MestReNova software (version 14.0). Data for ^{1}H-NMR spectra were reported as follows: chemical shifts are reported as δ in units of parts per million (ppm) relative to chloroform-d (δ 7.26, s); multiplicities are reported as follows: s (singlet), d (doublet), t (triplet), q (quartet), dd (doublet of doublets), m (multiplet), or br (broadened); coupling constants are reported as a J value in Hertz (Hz); the number of protons (n) for a given resonance is indicated as nH, and based on the spectral integration values. X-ray powder diffraction (XRD) patterns were obtained on a Bruker D8 advance spectrometer (Bruker AXS Inc., America). Inductively coupled plasma optical emission spectrometer (ICP-OES) analysis were acquired with Avio500 (PE, USA). Flow cytometry analysis was performed on the Coulter FC-500 flow cytometer. Analytical and semi-preparative high-performance liquid chromatography (HPLC) were carried out on Thermo Scientific Dionex Ultimate 3000 with CH_3CN/H_2O (1‰ CF_3COOH) as eluents. Matrix-Assisted Laser Desorption/Ionization Time of Flight Mass Spectrometry (MALDI-TOF-MS) analysis was conducted on the AB SCIEX 4800 Plus MALDI TOF/TOFTM mass spectrometer. The UV-Vis spectra were measured with an Ocean Optics Maya 2000 Pro spectrometer. The fluorescence spectra were measured with a HORIBA Jobin Yvon Fluoromax-4 fluorescence spectrometer with a 1 cm quartz cuvette. Dynamic light scattering (DLS) analysis were conducted on a 90 Plus/ BI-MAS equipment (Brook haven, USA). Transmission Electron Microscope (TEM) images and elements mapping analysis were acquired with a JEM-2800 Transmission Electron Microscope (JEOL, Ltd., Japan). Fluorescence images of cells and tissue slices were acquired with a Leica TCS SP8 confocal laser scanning microscope or an Olympus IX73 fluorescent inverted microscope. MTT assay was performed on a microplate reader (Tcan). Agarose gel electrophoresis visualized by Gel Doc^{TM} XR+ (Bio-Rad). In vivo fluorescence and bioluminescence images were recorded with an IVIS Lumina XR III system, and fluorescence and bioluminescence

intensity was quantified by the region-of-interest measurement using Living image software (PerkinElmer). Photoacoustic (PA) images were acquired with Vevo 2100 LAZR (FUJIFILM VisualSonics, Canada). Blood Count data were analyzed by BC2800Vet.

4. Supplementary Methods

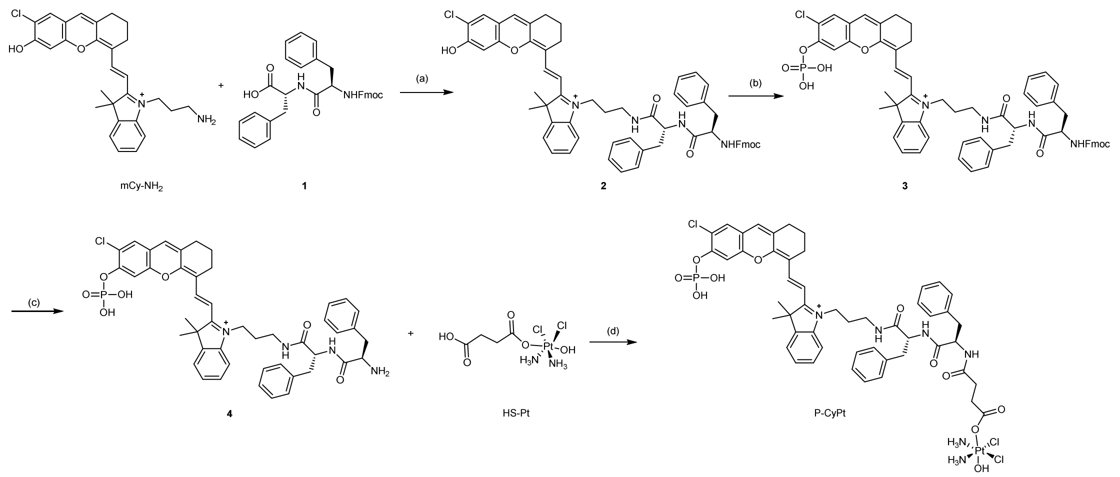

Supplementary Figure 37. Synthesis of P-CyPt. (a) HBTU, DIPEA, THF, r.t., 2 h, 87%; (b) (i) Diethyl chlorophosphate, DIPEA, r.t., 2 h; (ii) TMS-Br, r.t., 12 h, 51% for two steps; (c) 5% Piperidine, 0 ℃, 8 min, 90%; (d) HS-Pt, DMF, HATU, TEA, -20 ℃, 12 h, 45%.

Synthesis of compound **1**. Compound **1** was synthesized by standard solid-phase peptide synthesis (SPPS) using 2-chlorotrityl chloride resin and Fmoc-protected D-phenylalanine. Finally, compound **1** was cleaved from the resin using 1% trifluoroacetic acid/dichloromethane (TFA/DCM) solution. After removing the solvent, cold diethyl ether (Et_2O) was added, and the resulting precipitates were centrifuged to afford compound **1** (100.0 mg), which was dried and directly used for the next step. Yield: 93.6%. ^1H NMR (400 MHz, DMSO-d_6) δ 8.29 (d, J = 7.9 Hz, 1H), 7.87 (d, J = 7.5 Hz, 2H), 7.61 (q, J = 9.8, 9.1 Hz, 3H), 7.41 (td, J = 7.5, 3.2 Hz, 2H), 7.35 – 7.28 (m, 4H), 7.25 (s, 6H), 7.17 (t, J = 7.2 Hz, 2H), 4.54 – 4.45 (m, 1H), 4.29 (td, J = 14.5, 5.7 Hz, 1H), 4.19 – 3.96 (m, 4H), 3.10 (dd, J = 14.0, 4.5 Hz, 1H), 3.04 – 2.86 (m, 3H), 2.74 (d, J = 5.0 Hz, 1H). ^{13}C NMR (101 MHz, DMSO-d_6) δ 172.73, 171.59, 155.65, 143.74, 140.61, 138.09, 137.32, 129.20, 129.12, 128.15, 127.97, 127.58, 127.02, 126.42, 126.18, 125.31, 125.23, 120.03, 65.61, 55.91, 53.43, 46.50, 37.36, 36.64. MS: calcd. For $C_{33}H_{33}N_2O_5$ $[M + Na]^+$:557.2115; MALDI-MS found: m/z 557.0651.

Synthesis of compound **2**. A solution of mCy-NH_2 (68.0 mg, 0.14 mmol), compound **1** (74.8 mg, 0.14 mmol), O-Benzotriazol-1-yl-tetramethyluronium (HBTU) (68 mg, 0.18 mmol), and N,N-Diisopropylethylamine (DIPEA) (60 μL, 0.35 mmol) in dry THF (10 ml) was stirred at room temperature (r.t.) for 2 h. After the reaction, trifluoroacetic acid (THF) was removed under vacuum. The residue was purified by silica gel flash chromatography using CH_2Cl_2/CH_3OH (50:1 to 10:1) as an eluent to obtain compound

2 as a blue solid (136.8 mg, yield 87%). ^{1}H NMR (400 MHz, DMSO-d_6) δ 8.50 (d, J = 13.8 Hz, 1H), 8.20 (d, J = 19.6 Hz,1H), 7.82 (d, J = 7.6 Hz, 2H), 7.75 (d, J = 7.0 Hz, 1H), 7.65 (d, J = 7.8 Hz, 2H), 7.58 (d, J = 3.0 Hz, 1H), 7.56 (d, J = 3.6 Hz, 1H), 7.53 (d, J = 7.5 Hz, 1H), 7.46 (t, J = 7.5 Hz, 1H), 7.40 – 7.35 (m, 3H), 7.29 – 7.18 (m, 11H), 7.14 – 7.11 (m, 2H), 7.09 (s, 1H), 6.52 (d, J = 14.9 Hz, 1H), 4.49 (d, J = 7.3 Hz, 1H), 4.32 (t, J = 7.8 Hz, 1H), 4.27 (d, J = 6.2 Hz, 1H), 4.11 (dd, J = 12.5, 6.4 Hz, 3H), 3.24 (d, J = 6.5 Hz, 2H), 3.01(d, J = 7.4 Hz, 1H), 2.89 (d, J = 17.4 Hz, 2H), 2.73 (t, J = 11.5 Hz, 1H), 2.64 (d, J = 6.6 Hz, 4H), 1.94 – 1.83 (m, 2H), 1.80 – 1.65 (m, 8H). ^{13}C NMR (101 MHz, DMSO-d_6) δ 177.71, 171.85, 171.36, 160.66, 156.17, 152.70, 145.49, 144.17, 142.40, 141.71, 141.07, 138.43, 138.02, 132.83, 129.61, 129.41, 128.55, 128.44, 128.04, 127.53, 126.79, 126.64, 125.67, 123.20, 120.49, 118.47, 115.34, 114.63, 113.58, 104.78, 103.56, 66.13, 56.64, 54.82, 50.79, 46.98, 43.17, 38.13, 37.82, 36.43, 28.82, 27.86, 23.91, 20.31. MS: calcd. For $C_{61}H_{58}ClN_4O_6^+$ [M]$^+$: 977.4039; MALDI-MS found: m/z 977.2094.

Synthesis of compound **3**. Compound **2** (117.3 mg, 0.12 mmol) and DIPEA (210 µL, 1.2 mmol) were stirred in 5 mL dry DCM for 10 min under nitrogen protection. Diethyl chlorophosphate (35.0 µL, 0.24 mmol) in 2 mL DCM was then added dropwise, and the solution was then kept stirring at r.t. for another 2 h. The reaction mixture was then added with 30 mL DCM and washed with saturated Na_2CO_3 aqueous solution, HCl (1 M) and saturated NaCl aqueous solution for three times, and dried with anhydrous Na_2SO_4. The solvent was evaporated and the residue was dissolved in 5 mL DCM under nitrogen protection. TMS-Br (316 µL, 2.4 mmol) was added rapidly into the solution and the reaction mixture was stirred at r.t. for 24 h. The solution was then added dropwise into 100 mL methanol at -20 ºC to quench the reaction. DIPEA (420 µL, 2.4 mmol) in 10 mL cold methanol was added dropwise into the mixture to adjust the pH to neutral. The solvent was then evaporated, and the residue was purified by semi-preparative HPLC, using a gradient outlined in Table S11. Compound **3** was obtained as a blue solid after lyophilization (64.7 mg). Yield: 51 %. ^{1}H NMR (400 MHz, DMSO-d_6) δ 8.43 (d, J = 14.6 Hz, 1H), 8.26 (d, J = 7.2 Hz, 1H), 7.82 (d, J = 7.4 Hz, 2H), 7.76 (d, J = 7.0 Hz, 1H), 7.70 (d, J = 8.0 Hz, 1H), 7.65 (s, 1H), 7.59 (d, J = 7.7 Hz, 1H), 7.57 (s, 1H), 7.55 (s, 1H), 7.46 (t, J = 7.5 Hz, 1H), 7.37 (t, J = 7.4 Hz, 3H), 7.22 (d, J = 10.4 Hz, 11H), 7.15 – 7.10 (t, J = 6.5 Hz, 3H), 6.58 (d, J = 14.8 Hz, 1H), 4.49 (dd, J = 7.2 Hz, 7.6 Hz, 1H), 4.42 – 4.30 (m, 2H), 4.28 – 4.18 (m, 1H), 4.09 (d, J = 6.3 Hz, 3H), 3.24 (d, J = 6.7 Hz, 2H), 3.08 – 2.98 (m, 1H), 2.91 (t, J = 11.9 Hz, 2H), 2.77 – 2.68 (m, 1H), 2.63 (s, br, 4H), 1.87 (t, J = 7.3 Hz, 2H), 1.71 (m, 8H). ^{13}C NMR (101 MHz, DMSO-d_6) δ 178.20, 171.38, 170.94, 158.97, 155.67, 151.14, 150.24, 145.35, 143.66, 143.53, 142.26, 141.08, 140.56, 137.94, 137.55, 130.26,129.11, 128.93, 128.04, 127.93, 127.55, 126.99, 126.28, 126.14, 125.17, 122.82, 120.96, 120.87, 120.00, 117.75, 114.38, 113.47, 107.97, 105.62, 65.63, 56.18, 54.39, 50.69, 46.46, 43.05, 37.64, 37.32, 35.95, 28.53, 27.53, 27.17, 23.47, 19.65. MS: calcd. For $C_{61}H_{59}ClN_4O_9P^+$ [M]$^+$: 1057.3703; MALDI- MS found: m/z 1057.2734.

Synthesis of compound **4**. A mixture of piperidine/DMF (1:19, 8 mL) was stirred for 5 min under ice bath, then compound **3** (64.7 mg, 0.06 mmol) was added into the mixture

solution and stirred at 0 ºC for 10 min. When the reaction time was up, the prepared ice HCl (1M, 4.37 mL) was immediately added into the reaction solution at ice bath to adjust the pH to neutral. The solvent was then evaporated, and the residue was purified by semi-preparative HPLC, using a gradient outlined in Table S12. Compound **4** was obtained as a blue solid by lyophilization (45.1 mg). Yield: 90 %. ^{1}H NMR (400 MHz, DMSO-d_6) δ 8.94 (d, J = 8.0 Hz, 1H), 8.50 (d, J = 15.0 Hz, 1H), 8.41 (t, J = 5.6 Hz, 1H), 8.12 (s, br, 3H), 7.80 (d, J = 7.4 Hz, 1H), 7.71 (d, J = 7.9 Hz, 2H), 7.56 (t, J = 9.7 Hz, 2H), 7.49 (t, J = 7.4 Hz, 1H), 7.31 (s, 1H), 7.24 (d, J = 4.4 Hz, 4H), 7.20 (d, J = 6.1 Hz, 4H), 7.15 (q, J = 4.5, 4.0 Hz, 2H), 6.63 (d, J = 15.1 Hz, 1H), 4.50 (q, J = 8.1 Hz, 1H), 4.38 (m, 1H), 4.02 (s, 1H), 3.34 – 3.15 (m, 2H), 3.07 (dd, J = 14.1, 5.1 Hz, 1H), 2.99 (dd, J = 13.7, 6.1 Hz, 1H), 2.93 – 2.85 (m, 2H), 2.68 (d, J = 6.5 Hz, 4H), 1.89 (q, J = 7.4, 6.5 Hz, 2H), 1.80 (t, J = 5.6 Hz, 2H), 1.76 (s, 6H). ^{13}C NMR (101 MHz, DMSO-d_6) δ 178.88, 170.85, 168.28, 159.51, 158.77, 158.45, 151.65, 150.63, 145.93, 142.78, 141.68, 137.79, 135.11, 130.74, 129.99, 129.62, 129.44, 128.83, 128.66, 128.11, 127.44, 126.92, 123.39, 121.49, 121.41, 118.73, 118.36, 115.77, 114.89, 114.01, 108.55, 106.30, 55.10, 53.56, 51.28, 43.62, 38.28, 37.34, 36.48, 29.07, 28.05, 27.63, 24.08, 20.21. MS: calcd. For $C_{46}H_{49}ClN_4O_7P^+$ [M]$^+$:835.3022; MALDI-MS found: m/z 835.2222.

Synthesis of compound P-CyPt. Under N_2, compound **4** (25 mg, 0.03mmol), HATU (34.1mg, 0.09 mmol) and TEA (12.5 µL, 0.09 mmol) were stirred in 5 mL dry DMF at -20 ºC for 10 min, to which HS-Pt (24.9 mg, 0.06 mmol) in 2 mL DMF was added dropwise. The reaction solution was then kept stirring at -20 ºC for another 12 h. The solvent was then evaporated, and the residue was purified by semi-preparative HPLC, using a gradient outlined in Table S12. P-CyPt was obtained as a blue solid after lyophilization (16.9 mg). Yield: 45 %. The purity was confirmed by analytical HPLC on a C18 column (Phenomenex, 00G-4252-E0, 4.6 mm × 250 mm) using a gradient outlined in Table S13; HPLC retention time, t_R = 15.0 min (purity, 99 %). ^{1}H NMR (400 MHz, DMSO-d_6) δ 8.33 (d, J = 13.9 Hz, 1H), 8.16 (s, 1H), 8.09 (d, J = 7.1 Hz, 1H), 8.04 (d, J = 7.2 Hz, 1H), 7.67 (d, J = 7.1 Hz, 1H), 7.61 (d, J = 4.7 Hz, 2H), 7.54 (s, 1H), 7.46 (t, J = 7.5 Hz, 1H), 7.37 (t, J = 7.3 Hz, 1H), 7.23 (s, 1H), 7.16 (d, J = 5.5 Hz, 4H), 7.09 (d, J = 7.0 Hz, 3H), 7.04 (d, J = 7.2 Hz, 3H), 6.47 (d, J = 14.6 Hz, 1H), 5.96 (s, br, 6H), 4.40 – 4.24 (m, 4H), 3.19 – 3.16 (m, 2H), 2.95 (dd, J = 13.5, 4.8 Hz, 1H), 2.86 – 2.78 (m, 2H), 2.61 (d, J = 13.6 Hz, 5H), 2.31 (s, 1H), 2.16 (dd, J = 21.2, 8.9 Hz, 4H), 1.83 (s, 2H), 1.71 (s, 2H), 1.64 (s, 6H). ^{13}C NMR (101 MHz, DMSO-d_6) δ 180.45, 178.48, 172.40, 171.61, 171.42, 159.92, 152.27, 151.82, 145.72, 142.59, 141.68, 138.29, 138.17, 131.50, 129.63, 129.50, 129.39, 128.86, 128.55, 128.38, 127.76, 126.79, 126.56, 123.29, 121.37, 117.26, 114.74, 113.83, 108.08, 105.56, 54.90, 54.77, 51.19, 43.62, 40.57, 39.32, 37.91, 37.55, 36.58, 32.47, 32.17, 29.01, 27.97, 27.70, 24.88, 24.11, 20.23. MS: calcd. For $C_{50}H_{59}Cl_3N_6O_{11}PPt^+$ [M]$^+$: 1249.2667 (97.4%), 1250.2688 (100.0%), 1250.2700 (52.7%),1251.2637 (93.4%), 1251.2689 (74.6%), 1251.2721 (54.1%),1252.2658 (95.9%), 1252.2671 (50.5%), 1252.2723 (40.3%), 1253.2660 (71.5%), 1253.2608 (29.9%), 1253.2692 (51.8%), 1253.2719 (21.2%), 1254.2693 (38.7%), 1254.2629 (30.6%), 1255.2630 (22.9%), 1255.2689 (20.3%); ESI-MS found: m/z1249.2645, 1250.2686, 1251.2689, 1252.2689, 1253.2682, 1254.2675, 1255.2673.

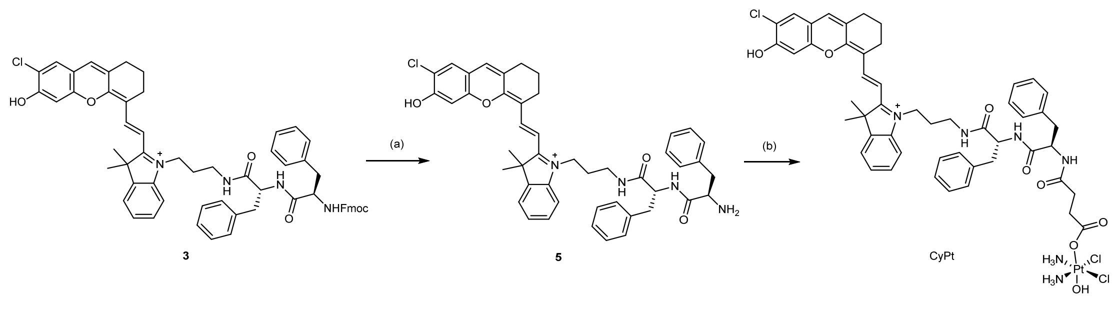

Supplementary Figure 38. Synthesis of CyPt. (a) 5% Piperidine, 0 ºC, 10 min, 92%; (b) HS-Pt, DMF, HATU, TEA, 0 ºC, 12 h, 52 %.

Synthesis of compound **5**. A mixture of piperidine/DMF (1:19, 8 mL) was stirred under ice bath for 5 min. Then, compound **3** (58.7 mg, 0.06 mmol) was added into the mixture solution and stirred at 0 ºC for 10 min. Cold aqueous HCl solution (1M, 4.37 mL) was immediately added into the reaction solution to adjust the pH to neutral. After being purification by semi-preparative HPLC using a gradient outlined in Table S12, compound **5** was obtained as a blue solid by lyophilization (41.7 mg). Yield: 92 %. ^{1}H NMR (400 MHz, DMSO-d_6) δ 8.88 (d, J = 7.9 Hz, 1H), 8.57 (d, J = 14.8 Hz, 1H), 8.33 (t, J = 5.7 Hz, 1H), 8.12 (s, 2H), 7.77 (d, J = 7.4 Hz, 1H), 7.71 – 7.62 (m, 2H), 7.56 (t, J = 7.7 Hz, 1H), 7.48 (t, J = 7.4 Hz, 1H), 7.42 (s, 1H), 7.27 – 7.24 (m, 4H), 7.22 - 7.20 (m, 4H), 7.18 – 7.14 (m, 3H), 6.55 (d, J = 14.9 Hz, 1H), 4.54 – 4.49 (m, 1H), 4.40 – 4.26 (m, 2H), 4.04 (s, 1H), 3.31 – 3.25 (m, 1H), 3.23 - 3.16 (m, 1H), 3.08 (dd, J = 14.1, 5.2 Hz, 1H), 3.01 – 2.83 (m, 4H), 2.71 – 2.65 (m, 4H), 1.92 – 1.79 (m, 4H), 1.76 (s, 6H). ^{13}C NMR (101 MHz, DMSO-d_6) δ 177.81, 170.75, 168.26, 162.81, 160.77, 157.15, 152.75, 145.53, 142.43, 141.76, 137.73, 135.10, 132.96, 130.00, 129.61, 129.40, 128.84, 128.66, 128.51, 127.58, 127.44, 126.93, 123.23, 118.52, 118.43, 115.49, 115.34, 114.61, 113.58, 104.83, 103.60, 54.97, 53.54, 50.83, 43.14, 38.32, 37.36, 36.43, 31.23, 28.84, 27.89, 23.97, 22.66, 20.34. MS: calcd. For $C_{46}H_{48}ClN_4O_4^+$ $[M]^+$: 755.3359; MALDI-MS found: m/z 755.2030.

Synthesis of probe CyPt. Compound **5** (22.6 mg, 0.03mmol), HATU (34.1 mg, 0.09 mmol) and TEA (12.5 µL, 0.09 mmol) were stirred in 5 mL dry DMF at -20 ºC for 10 min under nitrogen protection. Then, HS-Pt (24.9 mg, 0.06 mmol) dissolved in 2 mL DMF were added dropwise, and the solution was kept stirring at -20 ºC for 12 h. The solvent was then evaporated, and the residue was purified by semi-preparative HPLC, using a gradient outlined in Table S11. CyPt was obtained as a blue solid by lyophilization (18.3 mg). Yield: 52 %. The purity was confirmed by analytical HPLC on a C18 column (Phenomenex, 00G-4252-E0, 4.6 mm × 250 mm) using a gradient outlined in Table S13; HPLC retention time, t_R = 17.1 min (purity, >99 %). ^{1}H NMR (400 MHz, DMSO-d_6) δ 8.56 (d, J = 14.8 Hz, 1H), 8.11 – 8.06 (m, 3H), 7.76 (d, J = 7.1 Hz, 1H), 7.71 – 7.63 (m, 2H), 7.55 (t, J = 7.4 Hz, 1H), 7.47 (t, J = 7.4 Hz, 1H), 7.42 (s, 1H), 7.32 – 7.27 (m, 1H), 7.26 – 7.20 (m, 4H), 7.18 (d, J = 7.2 Hz, 3H), 7.13 (d, J = 6.8 Hz, 3H), 7.09 – 7.04 (m, 1H), 6.55 (d, J = 14.9 Hz, 1H), 6.14 (s, br, 6H), 4.46 – 4.40

(m, 2H), 4.37 – 4.28 (m, 2H), 3.28 – 3.17 (m, 2H), 3.01 (dd, J = 13.7, 5.5 Hz, 1H), 2.94 – 2.83 (m, 2H), 2.73 - 2.67 (m, 5H), 2.31 (d, J = 6.1 Hz, 2H), 2.27 – 2.20 (m, 2H), 1.91 – 1.84 (m, 2H), 1.80 (dd, J = 11.3, 6.0 Hz, 2H), 1.75 (s, 6H). ^{13}C NMR (101 MHz, DMSO-d_6) δ 180.15, 177.81, 172.13, 171.58, 171.29, 160.67, 152.73, 145.53, 142.43, 141.72, 138.28, 138.14, 132.81, 129.60, 129.53, 129.42, 128.57, 128.38, 127.55, 126.81, 126.57, 123.20, 118.43, 115.37, 114.63, 113.64, 104.91, 103.62, 54.78, 54.52, 50.84, 43.20, 40.59, 39.33, 37.94, 37.60, 36.48, 31.99, 28.86, 27.90, 27.78, 23.98, 20.35. MS: calcd. For $C_{50}H_{58}Cl_3N_6O_8Pt^+$ [M] $^+$: 1169.3003(97.4%),1170.3024(100.0%), 1170.3037(52.7%),1171.2974(93.4%),1171.3026(74.6%),1171.3058(54.1%),1172.29 95(95.9%),1172.3007,(50.5%),1172.3060(40.3%),1173.2944(29.9%),1173.3028(51.9 %),1173.3055(21.2%),1174.2965(30.6%),1174.3030(38.7%),1173.2997(71.5%),1175 .2967(22.9%),1175.3026(20.3%)),ESI-MS found: m/z 1169.3027,1170.3044, 1171.3024,1172.3016,1173.3000,1174.2998,1175.2982.

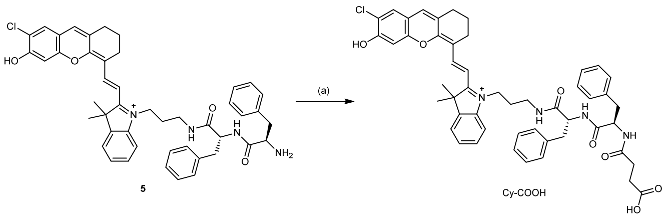

Supplementary Figure 39. Synthesis of Cy-COOH. (a) Succinic anhydride, DMSO, r.t., 12 h, 88%.

Synthesis of Cy-COOH. Compound **5** (22.6 mg, 0.03 mmol) and succinic anhydride (3.6 mg, 0.036 mmol) were dissolved in 5 mL dry DMSO and kept stirring at r.t. for 12 h. Then, the solvent was evaporated, and the residue was purified by semi-preparative HPLC, using a gradient outlined in Table S11. **Cy-COOH** was obtained as a blue solid by lyophilization (18.3 mg). Yield: 88 %. The purity was confirmed by analytical HPLC on a C18 column (Phenomenex, 00G-4252-E0, 4.6 mm × 250 mm) using a gradient outlined in Table S13; HPLC retention time, t_R = 18.7 min (purity, 99 %). ^{1}H NMR (400 MHz, DMSO-d_6) δ 8.55 (d, J = 14.2 Hz, 1H), 8.18 (d, J = 7.1 Hz, 1H), 8.08 (d, J = 7.3 Hz, 1H), 7.97 (s, 1H), 7.75 (d, J = 6.9 Hz, 1H), 7.67 (d, J = 8.1 Hz, 2H), 7.54 (t, J = 7.7 Hz, 1H), 7.47 (t, J = 7.4 Hz, 1H), 7.39 (s, 1H), 7.24 (d, J = 6.8 Hz, 4H), 7.20 – 7.15 (m, 3H), 7.14 – 7.08 (m, 4H), 6.55 (d, J = 14.9 Hz, 1H), 4.40 (dd, J = 10.9, 5.4 Hz, 2H), 4.31 (dd, J = 15.6, 7.6 Hz, 2H), 3.22 (m, 2H), 3.10 – 2.98 (m, 1H), 2.88 (dt, J = 12.6, 6.1 Hz, 2H), 2.72 – 2.65 (m, 5H), 2.29 (m, 4H), 1.93 – 1.83 (m, 2H), 1.78 (d, J = 6.2 Hz, 2H), 1.75 (s, 6H). ^{13}C NMR (101 MHz, DMSO-d_6) δ 177.29, 173.88, 171.51, 171.05, 170.77, 160.15, 156.58, 152.22, 145.05, 141.93, 141.23, 137.78, 137.69, 132.30, 129.06, 128.91, 128.08, 127.89, 127.04, 126.28, 126.09, 122.70, 117.93, 114.82, 114.14, 113.13, 104.35, 103.08, 54.40, 50.33, 42.69, 37.19, 37.05, 36.00, 29.87, 29.04, 28.36, 27.39, 27.28, 23.46, 19.84. MS: calcd. For $C_{50}H_{52}ClN_4O_7^+$ [M]$^+$: 855.3519; ESI-MS found: m/z 855.3521.

The dissipative particle dynamics simulation. The dissipative particle dynamics (DPD) method was introduced by Hoogerbrugge and Koelman in 1992 (*Europhys. Lett.* **1992**, *19*, 155) and then improved by Español and Warren in 1995 (*Europhys. Lett.* **1995**, *30*, 191). It is a widely used mesoscopic simulation approach (*J. Am. Chem. Soc.* **2014**, *136*, 2602; *J. Am. Chem. Soc.* **2013**, *135*, 7974; *Macromolecules*, **2017**, *50*, 5556; *Soft Matter*, **2020**, *16*, 3088; *J. Phys. Chem. B* **2013**, *117*, 9106). Briefly, in DPD simulations, an individual particle represents a cluster of atoms or molecules. The use of soft potential and a reduced number of interaction sites makes it a valuable approach to describe the phase morphology and dynamics of numerous soft matter systems at mesoscale while retaining the correct hydrodynamics. The time evolution of the simulation system is governed by integrating Newton's equation of motion (*J. Chem. Phys.* **1997**, *107*, 4423):

$$\frac{d\vec{r_i}}{dt}=\vec{v}_i,\ \frac{d\vec{v_i}}{dt}=\frac{\vec{f}_i}{m_i} \tag{1}$$

where $\vec{r}_i, \vec{v}_i, m_i,$ and $\vec{f}_i$ denote the position, velocity, mass of the *i* particle, and the force acting on it, respectively. The total force $\vec{f}_i$ acting on particle *i* is composed of conservative force F^C, random force F^R, and dissipative force F^D, each of them is pairwise interactions. The total force $\vec{f}_i$ is given by

$$\vec{f}_i = \sum_{j\neq i}\left(\vec{F}_{ij}^C + \vec{F}_{ij}^D + \vec{F}_{ij}^R\right) \tag{2}$$

The sum of force acts over all particles within a certain cutoff radius r_C, beyond which the force is neglected. Here r_C is the only length-scale in the system, which is considered as the unit of length, $r_C = 1$. The conservation force of two particles is soft-repulsive interaction acting along the line of the centers of two particles:

$$\vec{F}_{ij}^C = a_{ij}\omega(r_{ij})\hat{r}_{ij} \tag{3}$$

where a_{ij} is the interaction parameter between particles *i* and *j*. The *r*-dependent weight function $\omega(r_{ij})$ provides the range of interaction for DPD particles with a commonly used choice: $\omega(r_{ij}) = 1 - r_{ij}/r_C$ for $r_{ij} \leq r_C$ and $\omega(r_{ij}) = 0$ for $r_{ij} > r_C$. $\vec{r}_{ij} = \vec{r}_i - \vec{r}_j$, $r_{ij} = |\vec{r}_{ij}|$, and $\hat{r} = \vec{r}_{ij}/r_{ij}$. The dissipative force which is proportional to the relative velocity, $\vec{v}_{ij} = \vec{v}_i - \vec{v}_j$, is defined as

$$\vec{F}_{ij}^D = -\gamma\omega^D(r_{ij})(\hat{r}_{ij} \bullet \vec{v}_{ij})\hat{r}_{ij} \tag{4}$$

where γ is the friction coefficient controlling the magnitude of the dissipative force. The random force acting as a heat source to equilibrate the thermal motion of unresolved scales is given by

$$\vec{F}_{ij}^R = \sigma\omega^R(r_{ij})\theta_{ij}\hat{r}_{ij} \tag{5}$$

Where σ is the noise amplitude governing the intensity of the random force and $\theta_{ij}(t)$ is a randomly fluctuating variable with Gaussian statistics: $\langle\theta_{ij}(t)\rangle = 0$ and

$$\langle\theta_{ij}(t)\theta_{kl}(t')\rangle = (\delta_{ik}\delta_{jl} + \delta_{il}\delta_{jk})\delta(t - t').$$

Español and Warren[1] showed that the two weight functions $\omega^D(r_{ij})$ and $\omega^R(r_{ij})$ can be chosen arbitrarily and this choice fixes the other weight function, with the relation as shown in the following equation:

$$\omega^D(r_{ij}) = [\omega^R(r_{ij})]^2 \tag{6}$$

As a simple choice, we take $\omega^R(r_{ij}) = \omega(r_{ij})$, i.e. $\omega^R(r_{ij})$ is the same function as in the conservative force. There is also a relation between the two amplitudes and k_BT :

$$\sigma^2 = 2\gamma k_B T. \tag{7}$$

The combined effect of the dissipative and random force amounts to that of a thermostat.

The generic coarse-grained model (Supplementary Fig. 3a) is constructed based on P-CyPt and CyPt. There are three different types of DPD particles, A, B and C for Pt group, Cy group and P group, respectively. For simplicity, all beads have the same size of r_c and the same mass of $m = 1$. Within P-CyPt and CyPt, we use a finitely extensible nonlinear elastic (FENE) potential between the consecutive particles (*Phys. Rev. Lett.* 2000, 85, 1128-1131): $V_{FENE}(r_{ij}) = -1/2kR_0^2\ln[1-r_{ij}/R_0)^2]$ for $r_{ij} < R_0$ and $V_{FENE}(r_{ij}) = \infty$ for $r_{ij} \geq R_0$. In our simulation, we set $k = 50$ and $R_0 = 1.5r_c$. The time scale is set to $\tau = (mr_c^2/ k_BT)^{1/2}$, and the energy scale is given by $k_BT = 1$, where k_B is Boltzmann constant and T is the temperature. We use the modified Velocity-Verlet algorithm with $\lambda = 0.65$ in integrating the equation of motion. What's more, we set time step $\Delta t= 0.03\tau$ and the amplitude of random noise $\sigma = 3.0$ to avoid divergence of the simulation.

The molecules P-CyPt and CyPt can be represented by amphiphilic copolymer ABC and AB, which are dissolved in the solvent S, where S is the good solvent for A and C. The length of the block N_B is fixed at 8. The interaction parameters chosen are shown in the following symmetric matrix:

$$a_{ij} = \begin{Bmatrix} & \mathrm{A} & \mathrm{B} & \mathrm{C} & \mathrm{S} \\ \mathrm{A} & 25 & & & \\ \mathrm{B} & 28 & 25 & & \\ \mathrm{C} & 25 & 28 & 25 & \\ \mathrm{S} & 25 & 35 & 25 & 25 \end{Bmatrix}$$

When we fix the three-dimension simulation with a fixed system number density of 3.0, the relationship between the a_{ij} and Flory-Huggins interaction parametersχ_{ij} is:

$$a_{ij} \approx a_{ii} + 3.497\chi_{ij}. \tag{8}$$

We set $a_{ii} = 25$ for the same type of DPD particles (i = A, B, C, S), guaranteeing the correct excluded volume of the molecules. a_{ij} rises from 25 with increasing the

incompatibility between particles i and j. Considering the relative position and chemical components of all the beads, we always keep $a_{AB} = a_{AC} = 28$, $a_{AS} = a_{CS} = 25$, and $a_{BS} = 35$. The concentration of ABC or AB is fixed at 0.03. We performed the dynamics of total 81 000 DPD beads in a cubic box (30^3) under the periodic boundary conditions. In these conditions, we carry out 1.0×10^6 steps for each simulation.

Molecular simulation using the Materials Studio program. For each molecule, the most favourable crystal packing was predicted using the Polymorph module as implemented in Materials Studio. The crystal structure prediction protocol includes three parts, Packing, Geometry Optimization and Clustering. The main part of the procedure is the Packing phase, in which the space of possible crystal structures is searched for candidates of low energy. In addition, the space groups of interest must be provided (usually a small subset is required, not the full set of 230 space groups). In the present study, the most frequent 10 space groups ($P2_1/c$, P-1, $P2_12_12_1$, $C2/c$, $P2_1$, *Pbca*, $Pna2_1$, *Cc*, *Pbcn* and *C*2) were provided in the Packing phase, and Monte Carlo Simulated Annealing was used as the search algorithm. Smart algorithm was used in the Geometry Optimization phase. Ewald summation method and Atom based summation method were used for electrostatic and van der Waals interactions, respectively. All energy calculations were performed with the Universal force field.

Evaluation of the dephosphorylated efficacy of P-CyPt towards ALP. P-CyPt (10 μM) in 100 μL Tris buffer was placed in a 96-well black plate. The reactions were initiated upon addition of varying concentrations of 100 μL ALP (0, 10, 20, 40, 100, 200, 400 and 1000 U/L) to each solution. The resulting fluorescence intensities in each well in the first 30 minutes were recorded on a Spark™ 10M Multimode Microplate Reader ($\lambda_{ex/em}$ = 680/710 nm), and every two-minute intervals record one point. The amount of **CyPt** at each time point was determined by a standard curve under the same conditions.

Log*P* measurement in solutions. P-CyPt or CyPt (0.2 mM) in 1.0 mL octanol was subjected to partition with 1.0 mL octanol-saturated water. The resulting mixture was stirred vigorously for 5 min and centrifuged at 2040 × *g* for 5 min. Then, the octanol layer and water layer were separated. The UV-vis absorption spectroscopy or fluorescence spectrum was recorded after being diluted in MeOH/H_2O (1:1) system (<1% octanol). The log*P* value was calculated by dividing the amount in the octanol layer to that in water layer.

Release of CDDP from Pt^{IV}NPs during disassembly process. P-CyPt (10 μM) in 5 mL Tris buffer was incubated with ALP (100 U/L) at 37 °C for 30 min to form Pt^{IV}NPs, followed by addition with 10 mM GSH at 37 ºC. After 10, 20, 30, 40, 50 and 60 min, 50 mM *N*-ethylmaleimide was added into the solution to scavenge GSH. The reaction solution was centrifuged at 13,000 × *g* and the supernatant was collected. The supernatants were digested with concentrated HNO_3 (65~70%) and HCl (36~38%) at 90 °C for 24 h. The solution was diluted to 5 mL 2% HNO_3 and 5% HCl solution, and the concentration of Pt element was measured by ICP-OES, which was further applied

to calculate the amount of CDDP (denoted as N_{Pt}) released in the solution. The percentage of Pt release at each time point was calculated according to the formula:

$$\text{Pt release } \% = N_{Pt}/N_0 \times 100\% \qquad (9)$$

Where N_0 represents the total amount of Pt in the the in situ formed Pt^{IV}NPs, and N_{Pt} represents the amount of Pt released from Pt^{IV}NPs following incubation with GSH.

Characterization of enzyme kinetics of P-CyPt towards ALP. Briefly, varying concentrations of P-CyPt (0.5, 1.0, 2.0, 3.0, 4.0, and 5.0 μM) in 100 μL Tris buffer were placed in a 96-well black plate. The reactions were initiated upon addition of 100 μL ALP (30 U/L) to each solution. The resulting fluorescence intensities in each well in the first five minutes were recorded on a Spark™ 10M Multimode Microplate Reader ($\lambda_{ex/em}$ = 680/710 nm). The amount of CyPt at each time point was determined by a standard curve under the same conditions. Kinetics values, including *Km* and *Vmax*, were determined according to the Lineweaver-Burk plot.

Determination of the sensitivity of P-CyPt towards ALP. To evaluate the sensitivity of P-CyPt towards ALP, P-CyPt (10 μM) was incubated with varying concentrations of ALP (0, 0.5, 1, 2, 5, 10, 15, 20, 50, 100, 150 and 200 U/L) in Tris buffer at 37 °C for 30 min, and the fluorescence spectra were then recorded on a HORIBA Jobin Yvon Fluoromax-4 fluorometer, with an excitation at 680 nm. The resulting fluorescence intensities at 710 nm were plotted to the concentrations of ALP, and a linear regression fitted from 0 -20 U/L ALP was obtained, affording the slope *k*. The detection limit was calculated from $3\sigma/k$, where σ represents the standard deviation of 11 blank measurements.

Determination of the specificity towards ALP. The specificity towards ALP *in vitro* was evaluated based on fluorescence, respectively. P-CyPt (10 μM) in Tris buffer was incubated with 10 nmol/L of MMP-2, 100 U/L of γ-glutamyl transferase (GGT), 100 U/L Cathepsin B (CTB), 0.2 μg/mL of Caspase-3/7, 2.5 mg/mL of Trypsin, 100 U/L of ALP or 100 U/L ALP pretreated with its inhibitors, Na_3VO_4 (100 μM) for 10 min. The solutions were kept at 37 °C for 30 min. The fluorescence spectra were then acquired with an excitation at 680 nm.

Immunofluorescence staining of membrane-bound ALP. Briefly, HeLa cells were grown onto at glass-bottom dish and allowed to grow overnight. The cells were fixed with 4% paraformaldehyde for 15 min, treated with 0.1% Triton X-100-PBS for 1 h, and then blocked with 1% bovine serum albumin (BSA)/10 % normal goat serum. Then, the fixed HeLa cells were incubated with the primary antibody of tissue nonspecific ALP (Mouse monoclonal [2F4] to ALP, Tissue Non-Specific, ab126820, lot: GR3428682-4, dilution 1:100) at 4°C overnight. After being washed with PBS three times, the cells were then incubated with the Alexa Fluor®488-labeled secondary antibody (Alexa Fluor®488 goat anti-rabbit IgG (H+L), ab150077, lot: GR3376391-4, dilution 1:1000) at room temperature for 1 h. After that, Hoechst 33342 was used to stain the cell nuclei (blue). Last, cells were washed with PBS three times and mounted for imaging. The confocal fluorescence using the IX73 optical microscope equipped

with DAPI and FITC filters, microscope (Olympus, IX73) with U plan supper apochromat objective 60x/1.35 oil lens (Olympus), and captured at 8 bit on a DP80 detector (Olympus), the analysis of the pictures captured using the microscope equipped with DAPI and FITC filters.

General procedure for fluorescence imaging of cells. Cells (~ 5×10^4) were seeded onto a glass-bottom dish (In Vitro Scientific, D35-20-1-N) and allowed to grow overnight. P-CyPt, or Pt^{IV}NPs (10 μM) in FBS free DMEM was added into dishes and incubated at 37 °C for 30 or 60 min. To inhibit ALP activity, cells were pretreated with ALP inhibitor Na_3VO_4 (10 mM) for 20 min, and then incubated with P-CyPt (10 μM) for another 30 or 60 min. The medium was removed, gently washed with 1 mL PBS buffer once. After adding fresh medium, Fluorescence images were captured on a Leica TCS SP8 confocal laser scanning microscope, microscope (TCS SP8 STED 3X) with 63x/1.4 oil lens (Leica), and captured at RGB on a HyD detector (Leica Dmi8), with the excitation wavelength at 670 nm, and the emission wavelength from 690 nm to 750 nm.

Co-localization study. To examine the intracellular location, HeLa cells were incubated with 2 μM Hoechst 33342 and 200 nM Lyso Tracker Green DND-26 for 20 min. After being washed with PBS for three times, the cells were then incubated with 10 μM P-CyPt for another 1 h. The cells were washed gently with 1 mL PBS buffer once. After adding fresh DMEM medium, the fluorescence images of the cells were captured on the confocal laser scanning microscope, using blue, green and NIR channels.

Investigation of Endocytosis pathway. Three endocytosis inhibitors, including chlorpromazine (CPZ) for clathrin-mediated endocytosis, filipin III for caveolae-mediated endocytosis and ethylisopropyl amiloride (EIPA) for micropinocytosis were used to determine the possible pathways of cellular uptake of assembled Pt^{IV}NPs. HeLa cells were treated with respective inhibitor (30 μM CPZ, 5 μg/mL filipin III or 100 μM EIPA) for 30 min, washed with PBS for three times, and then treated with 10 μM P-CyPt for another 1 h. After being washed with 1 mL PBS once, the fluorescence images were acquired using the confocal laser scanning microscope.

Bimodality FL and PA imaging of cell pellets. To examine the ability for FL and PA bimodality imaging of ALP activity in cells, HeLa or HEK29T cells were seeded in 10-cm dishes at a density of 4×10^6 cells/well and allowed to grow overnight. P-CyPt or Pt^{IV}NPs (10 μM) in 4 mL FBS free DMEM was added into wells and incubated at 37 °C for 30 min. To inhibit ALP activity, cells were pretreated with inhibitor Na_3VO_4 (10 mM) for 20 min, and then incubated with P-CyPt (10 μM) for another 30 min. To evaluate the disassembly of in situ formed Pt^{IV}NPs of P-CyPt in response of intracellular GSH, P-CyPt (10 μM) in 4 mL FBS free DMEM was added into wells and incubated at 37 °C for 30 min, then the medium was removed and the cells were incubated for another 3 h after adding fresh medium. Then, the medium was removed, and cells were washed gently with 1 mL PBS once. Trypsin (1 mL) was added into each dish, maintained at 37 °C for 2 min to detach the cells. The cell pellets were then

collected after centrifugation at 161× *g* for 4 min. The fluorescence imaging and PA imaging of the cell pellets were then acquired on an IVIS Lumina XR III system ($\lambda_{ex/em}$ = 670/750 ± 50) and VisualSonics Vevo 2100 LAZR system at 700 and 750 nm, respectively.

HPLC analysis of cell pellets. HeLa or HEK29T cells were seeded in 6-cm dishes at a density of 1×10^6 cells/well and allowed to grow overnight. P-CyPt or Pt^{IV}NPs (10 μM) in 2 mL FBS free DMEM was added into wells and incubated at 37 ºC for 30 min. To inhibit ALP activity, cells were pretreated with inhibitor Na_3VO_4 (10 mM) for 20 min, and then incubated with P-CyPt (10 μM) for another 30 min. To evaluate the disassembly of in situ formed Pt^{IV}NPs of P-CyPt in response of intracellular GSH, P-CyPt (10 μM) in 4 mL FBS free DMEM was added into wells and incubated at 37 °C for 30 min, then the medium was removed and the cells were incubated for another 3 h after adding fresh medium. Then, the corresponding culture mediums were collected. The cells were trypsinized, centrifuged and counted. After being lysed with 200 μL DMSO, the cell lysates were mixed with 300 μL cold MeOH and 500 μL D.I. water, and centrifuged at 13,000× *g* at 4 °C for 10 min. Aliquots of supernatant containing cell lysates (250 μL) and 500 μL of culture medium were injected into an HPLC system for analysis, respectively.

To establish the standard curve, varying concentrations of P-CyPt, CyPt or Cy-COOH (0.5-10 μM) was injected into analytical HPLC, and the corresponding peak area at each concentration was integrated to give a linear standard curve of HPLC peak area versus the concentration of P-CyPt, CyPt or Cy-COOH. Using the established standard curves, the concentrations of P-CyPt, CyPt and Cy-COOH in the lysates could be calculated, which were further divided by the number of cells, giving the cellular uptake of them (fmol/cell).

TEM analysis of the in situ self-assembled Pt^{IV}NPs in HeLa cells. To further demonstrate the formation of NPs in culture HeLa cells, HeLa cells were seeded on a 6-well cell culture plats at a density of 2×10^5 cells/well. After growing overnight, the cells were incubated with or without P-CyPt (10 μM) for 1 h and washed gently with cold PBS buffer for three times. Afterwards, trypsin (500 μL/well) was added into each well, maintained at 37 °C for 2 min to detach the cells. The cell pellets were then collected, and lysed via repeated freezing and thawing in liquid nitrogen. The cell lysates were separated according to a reported fragmentation procedure to obtained different cell fractions: pellet sample N (nuclei) was separated by 600 g for 10 min; pellet sample L (lysosomes and mitochondria) separated by 15000 g for 5 min; pellet sample M (plasma membrane) separated 100000g for 60 min and the remaining supernatant was soluble portion cytoplasm named as cell pellet C (cytosol) Pellets N, L, and M were all re-dispersed in 200 μL of D.I. water. Then, the solutions were collected and dropped on a carbon-coated copper grid, followed by freeze-drying respectively. The samples were then examined in a JEM-2800 Transmission Electron Microscope.

Quantitative analysis of Pt in HeLa cells and different subcellular organelles. HeLa

cells were seeded in 10-cm dishes at a density of 4×10^6 cells/well and allowed to grow overnight. The cells were incubated with P-CyPt (10 μM), Pt^{IV}NPs (10 μM) and CDDP (10 μM) for 1 h, respectively. In P-CyPt + Na_3VO_4 group, HeLa cells were first incubated with Na_3VO_4 (10 mM) for 20 min, and then incubated with P-CyPt (10 μM) for another 60 min. After being washed with PBS for three times, the cells were trypsinized, centrifuged and counted. For Pt distribution analysis, the cells were treated with P-CyPt (10 μM) or CDDP (10 μM) for 1 h, and then washed with PBS for three times, followed by incubation with fresh medium for another 24 h. After that, cells were washed gently with 2 mL PBS once. The cells were trypsinized, centrifuged and counted. The cell lysates were separated according to a reported fragmentation procedure to obtained different cell fractions, including N (nuclei), M (plasma membrane), L (lysosomes and mitochondria) and C (cytosol). The Cells and different cell fractions were digested with concentrated HNO_3 (65~70%) and HCl (36~38%) at 90 °C for 24 h. The solution was diluted to 5 mL 2% HNO_3 and 5% HCl solution, and the concentration of Pt element was measured by ICP-OES, which was further applied to calculate the amount of Pt in cells and subcellular organelles.

Flow cytometry analysis of HeLa cell death. Approximately 2×10^5 cells/well HeLa cells in 2 mL DMEM medium were seeded onto 6-well plates and allowed to grow overnight. The medium was replaced with 1 mL of fresh medium containing P-CyPt (20 μM), Pt^{IV}NPs (20 μM) and CDDP (20 μM), respectively. After 2 h incubation, medium was removed and cells were washed with PBS for three times. Then, cells were incubated with fresh medium for another 48 h to induce different levels of apoptosis. After gentle washing with PBS (×1), the cells were detached from the plates with trypsin (500 μL per well), and the cell pellets were collected after centrifugation at 1000 161× *g* at 4 °C for 4 min. After washing with PBS, the pellets were re-suspended in 500 μL PBS buffer and stained with the annexin V-FITC/PI double staining apoptosis detection kit for flow cytometry. All assays were performed according to the manufacturer instructions, and the cell population was analyzed by annexin V-FITC and PI channels. The count number for each flow cytometry analysis was ~5000 to 10000, and the data was processed using FlowJo software.

Evaluation of the ability to break DNA double strand. Supercoiled pBR322 plasmid DNA (20 ng μL^{-1}) was incubated with blank Tris buffer (i) or Tris buffer containing GSH (2 mM, ii), Tris buffer containing CDDP (100 μM, iii), Tris buffer containing GSH (2 mM) and CDDP (100 μM, iv), Tris buffer containing P-CyPt (100 μM, v), Tris buffer containing P-CyPt (100 μM) and GSH (2 mM, vi), Tris buffer containing P-CyPt (100 μM) and ALP (500 U/L, vii), and Tris buffer containing P-CyPt (100 μM), ALP (500 U/L) and GSH (2 mM, viii) or incubated with P-CyPt ((0, 1, 2, 5, 10, 20, 50 and 100 μM, from i-viii) respectively in the presence of ALP (500 U/L), followed by incubation with GSH (2 mM) in Tris buffer) at 37 ºC for 16 h. Then, the reactions (10 μL) were quenched by loading buffer (2 μL, including 30 mM EDTA, 36% glycerol, 0.05% xylene cyanol FF, and 0.05% bromophenol blue). The resulting solutions and 1kb-I DNA ladder (GsDL10001) were loaded into native agarose gel (1%) and subjected to electrophoresis in a TAE buffer (40 mM Tris acetate, 1 mM EDTA). The

DNA simples pre-stained with 5x loading buffer in the TAE buffer and the resultant gel visualized by Gel Doc^{TM} XR+ (Bio-Rad) system.

Depletion of intracellular GSH. HeLa cells (2×10^5 cells/well) in 2 mL DMEM medium were seeded onto 6-well plates and allowed to grow overnight. Then cells were then treated with P-CyPt, Pt^{IV}NPs, or CDDP (10 μM), respectively. After that, cells were digested with trypsin and washed with PBS for 3 times. Then, cell pellets were dispersed in pre-chilled extraction solution (1 mL, 0.1 M potassium phosphate buffer containing 0.1% Triton, 0.6% sulfosalicylic acid, and 5 mM EDTA, pH 7.4) and sonicated with an US probe on ice for 2 min. To ensure that cells were lysed completely, the solution was further frozen and thawed twice. Finally, the solution was centrifuged at 3000 g at 4 °C for 4 min. The supernatant was taken to measure the GSH content using commercial GSH detection kit, according to a reported procedure (*Nat. Protoc.* ***2006**, 1,* 3159).

MCTS imaging and cytotoxicity. The three-dimensional multicellular spheroids (MCTS) of HeLa were cultured according to the method previously described (*Nat. Protoc.* ***2009**, 4,* 309) with minor modifications. Briefly, a well of 96-well plates was covered by 50 μL of hot agarose solution (1.5 w/v %) and then cooled to form a layer of agaropectin. HeLa cells were seeded to the agaropectin-coated well at a density of 2×10^3 cells/well, and incubated to grow into spheroids. DMEM medium was replaced with fresh medium every 2 days until the diameter of tumor spheroids grows near 400 μm. Then, the MCSs were incubated with 10 μM P-CyPt or Pt^{IV}NPs for 1 h, washed with PBS for 3 times. Blank DMEM medium was added, and incubated for another 24 h. After that, PI (4 μM) was added and incubated for 1 h. The medium was removed, and the spheroids were gently washed with PBS (×1), which was then carefully transferred to a glass bottom dish. The fluorescence images of the MCTS were captured on a Leica TCS SP8 confocal laser scanning microscope, microscope (TCS SP8 STED 3X) with 10x/0.4 dry lens (Leica), and captured at RGB on a HyD detector (Leica Dmi8) under PI and Cy5.5 channels.

Ex vivo FL imaging of resected organs. Mice bearing s.c. HeLa tumors were i.v. injected with P-CyPt (100 μM) or Pt^{IV}NPs (100 μM) in 200 μL saline, and the mice were sacrificed after 4 h. Tumors and main organs, including liver, kidneys, intestines, heart, lung, stomach and spleen were resected. The fluorescence images of these organs and tumors were acquired with the IVIS Lumina XR III imaging system using a 660 nm excitation filter and a 750 ± 50 nm emission filter. Each experiment was conducted in three mice.

Biodistribution study of Pt. HeLa tumor-bearing mice were i.v. injected with P-CyPt (100 μM), Pt^{IV}NPs (100 μM) or CDDP (100 μM) in 200 μL saline. Mice were sacrificed at 4 h post injection. Tumors and major organs including heart, liver, spleen, lung, and kidneys, were collected and weighed. The tissues were cut into small pieces and digested with concentrated HNO_3 (65-70%, 2 mL) and HCl (36~38%, 1 mL) at 90 °C overnight. The residue in each organ was then diluted with 5 mL 2% HNO_3 and 5% HCl solution, and the concentration of Pt was determined by ICP-OES. The %ID/g was

calculated for comparison.

HPLC analysis of tumor tissues. HeLa tumor-bearing mice were i.v. injected with P-CyPt (100 μM) or Pt^{IV}NPs (100 μM) in 200 μL saline. Mice were euthanized and their tumors were resected after 4 h. The tumor tissues were cut into small pieces, to which NEM (100 mM, 200 μL), PBS buffer (200 μL) and RIPA buffer (200 μL) were added. The mixtures were sonicated in ice bath for 5 min to prepare the tumor homogenates. Then, 200 μL DMSO were added into the homogenates and sonicated for another 5 min to dissolve compounds. Finally, 200 uL methanol were added into the homogenates and centrifuged at 2,700× *g* for 10 min to remove proteins. The supernatant was injected into HPLC to analyze the compounds.

Fluorescence imaging of tumor tissue slices. Mice bearing s.c. HeLa tumors were i.v. injected with P-CyPt (100 μM) or Pt^{IV}NPs (100 μM) in 200 μL saline, and the mice were sacrificed after 4 h. The tumors were resected and cut using a vibrating-blade microtome to obtain 10 μm-thickness slices. After staining with DAPI, the fluorescence images of tumor tissue slices were acquired with the Olympus IX73 fluorescent inverted microscope, using the microscope equipped with DAPI and Cy5.5 filters.

Histopathological staining. HeLa tumor-bearing mice were i.v. injected with PBS (200 μL), P-CyPt, Pt^{IV}NPs, or cisplatin (2.25 mg kg^{-1} Pt). After 2 days, the HeLa tumors were excised and fixed in formalin. Then, the tumors were cryosectioned at 10-μm thickness and stained with anti-gammaH2A.X (ab81299, lot: GR3338944-19, dilution 1:200) at 4°C overnight. After being washed with PBS three times, the cells were then incubated with the Alexa Fluor®488-labeled secondary antibody (Alexa Fluor®488 goat anti-rabbit IgG (H+L), ab150077, lot: GR3376391-4, dilution 1:1000) at room temperature for 1 h. After that, Hoechst 33342 was used to stain the cell nuclei), H&E and TUNNEL (KeyGen Biotech. Co. Ltd., Nanjing, China) staining kit, respectively, according to the manufacturer's instructions. The confocal fluorescence using the IX73 optical microscope equipped with DAPI and FITC filters, microscope (Olympus, IX73) with U plan semi Apochromat phase objective 10x/0.3 dry lens (Olympus), and captured at 8 bit on a DP80 detector (Olympus), the analysis of the pictures captured using the microscope equipped with DAPI and FITC filters.

To examine the potential side effects, major organs including liver, spleen, kidney, heart, and lung were resected from, mice on 21-day post 5-dose treatment with PBS, P-CyPt, Pt^{IV}NPs, or cisplatin (2.25 mg kg^{-1} Pt), and applied for histopathological analysis.

Blood analysis. Mice were i.v. injected with P-CyPt, Pt^{IV}NPs, CDDP (2.25 mg kg^{-1} Pt) or PBS. The bloods (~1.0 mL) were collected from the venous sinus at 24 h post-injection, and preserved into 1.5-mL EDTA coated Eppendorf tubes that are chilled on ice. The collected blood samples were divided into two parts. Part one was applied for blood count analysis. Part two was centrifuged at 2,400 × *g* for 20 min. Biochemistry relative biomarkers including alanine aminotransferase (ALT), glutamic oxaloacetic transaminase (AST), γ-glutamyl transpeptidase (GGT), total bilirubin (TBil), albumin (Alb), total protein (TP), blood urea nitrogen (BUN) and creatinine (CRE) were

determined using ELISA kits according to manufacturer's protocol.

Determination of the blood circulation half-life. P-CyPt, **Pt^{IV}NPs**, or CDDP (at a dose of 2.25 mg kg^{-1} Pt) was i.v. injected into healthy female BALB/c nude mice (6-8 weeks old). After injection at 0, 0.5, 1, 2, 4, 8, 12, and 24 h, around 1.2 mL of bloods from every three mice at each time point were collected from the venous sinus and preserved into 1.5-mL EDTA-coated Eppendorf tubes that are chilled on ice. The collected blood was digested with concentrated HNO_3 (65-70%, 2 mL) and HCl (36~38%, 1 mL) at 90 °C overnight. The residues were then diluted with 5 mL 2% HNO_3 and 5% HCl solution, and the concentrations of Pt element were determined by ICP-OES, which were further applied to calculate the contents of Pt element in the blood. The plot of the percentage of Pt element remained in the blood versus injection time to yield the pharmacokinetic time.

Urine and Faeces analysis. Healthy female BALB/c nude mice were i.v. injected with P-CyPt, Pt^{IV}NPs or CDDP (2.25 mg kg^{-1} Pt, dissolved in saline) and placed in metabolic cages for 12 h. Urine and faeces were collected at 2 (for 0-2 h), 4 (for 2-4 h), 8 (for 4-8 h) and 12 h (for 8-14 h), which were diluted in methanol and centrifuged at 3,600 × *g* for 10 min, followed by filtering via a 0.22 μm syringe filter. The solutions were analyzed by HPLC (660 nm detection) to characterize all the mCy-containing compounds. Using the established standard curves for P-CyPt, CyPt and Cy-COOH, the amount of each compound in the urine and faeces could be calculated, which were further applied to determine the percentage of them in the urine and faeces. The data were summarized in Supplementary Tables 5-8.

Healthy female BALB/c nude mice were i.v. injected with P-CyPt, Pt^{IV}NPs or CDDP (2.25 mg kg^{-1} Pt, dissolved in saline) and placed in metabolic cages for 12 h. Urine and faeces were collected and weighed. The urine and faeces were digested with concentrated HNO_3 (65~70%) and HCl (36~38%) at 90 °C for 24 h. The solution was diluted to 5 mL 2% HNO_3 and 5% HCl solution, and the concentration of Pt element was measured by ICP-OES, which was further applied to calculate the percentage of Pt in corresponding urine and faeces. The data were summarized in Supplementary Tables 9 and 10.

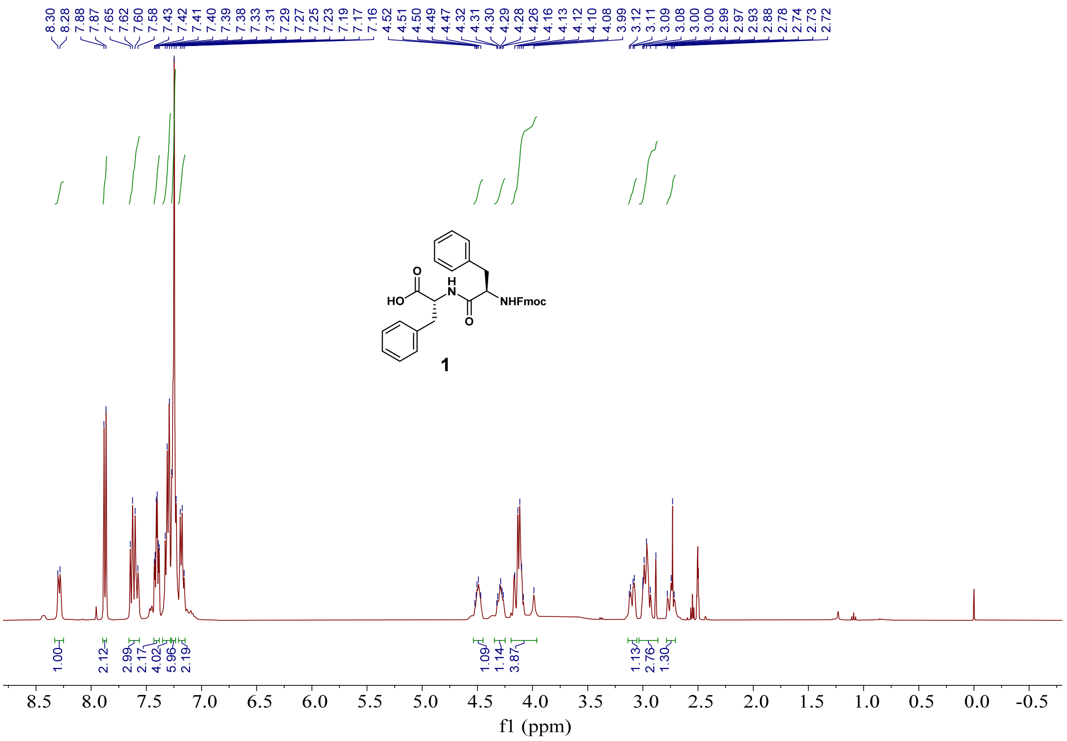

Supplementary Figure 40. ^{1}H-NMR spectra of compound **1** (400 MHz, DMSO-d_6).

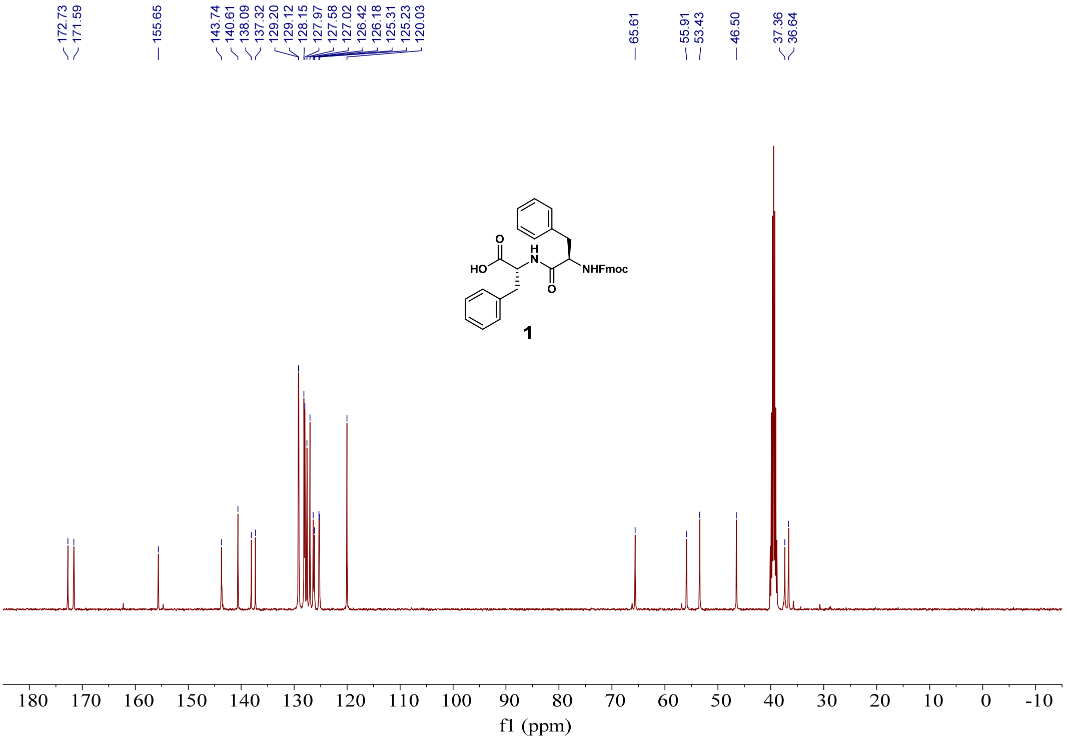

Supplementary Figure 41. ^{13}C-NMR spectra of compound **1**(101 MHz, DMSO-d_6).

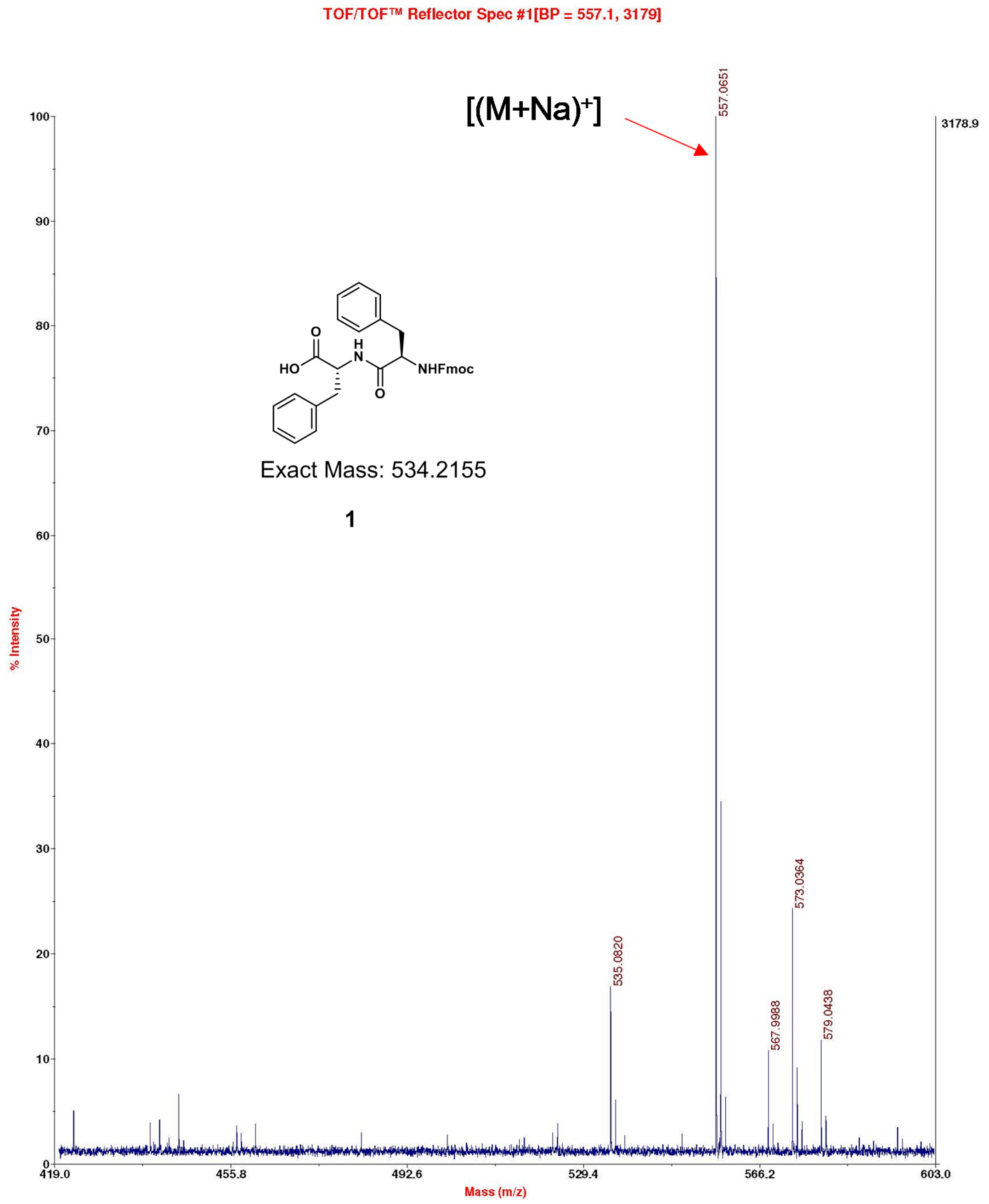

Supplementary Figure 42. MALDI-MS spectra of compound **1**.

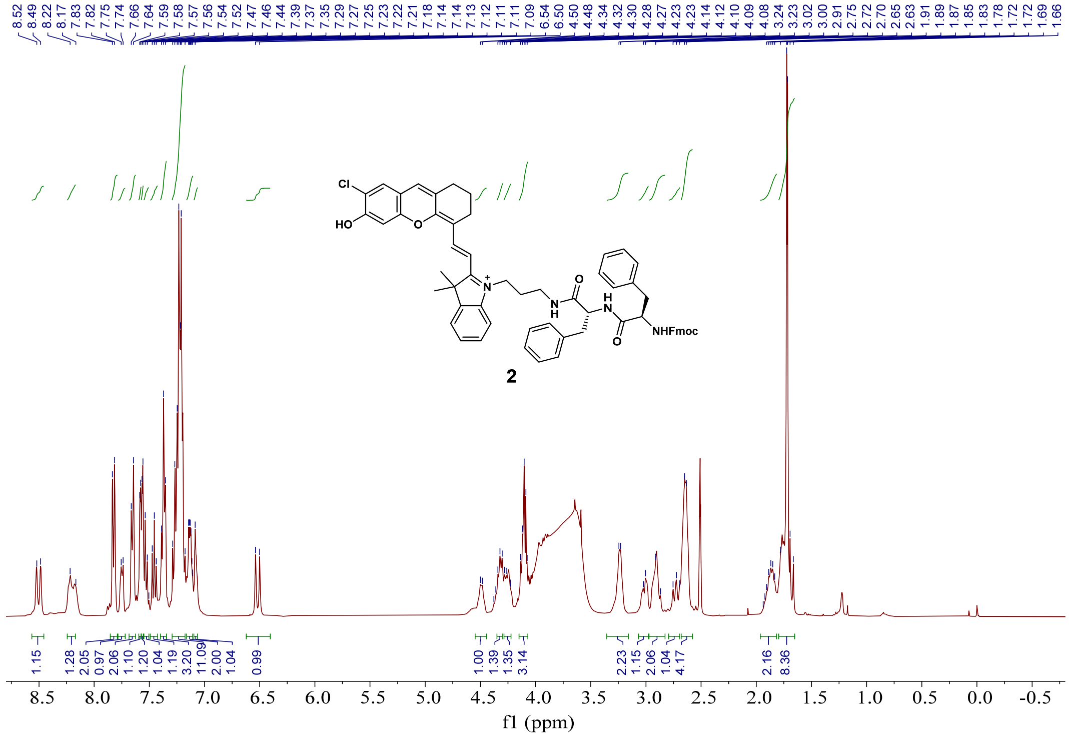

Supplementary Figure 43. ^{1}H-NMR spectra of compound **2** (400 MHz, DMSO-d_6).

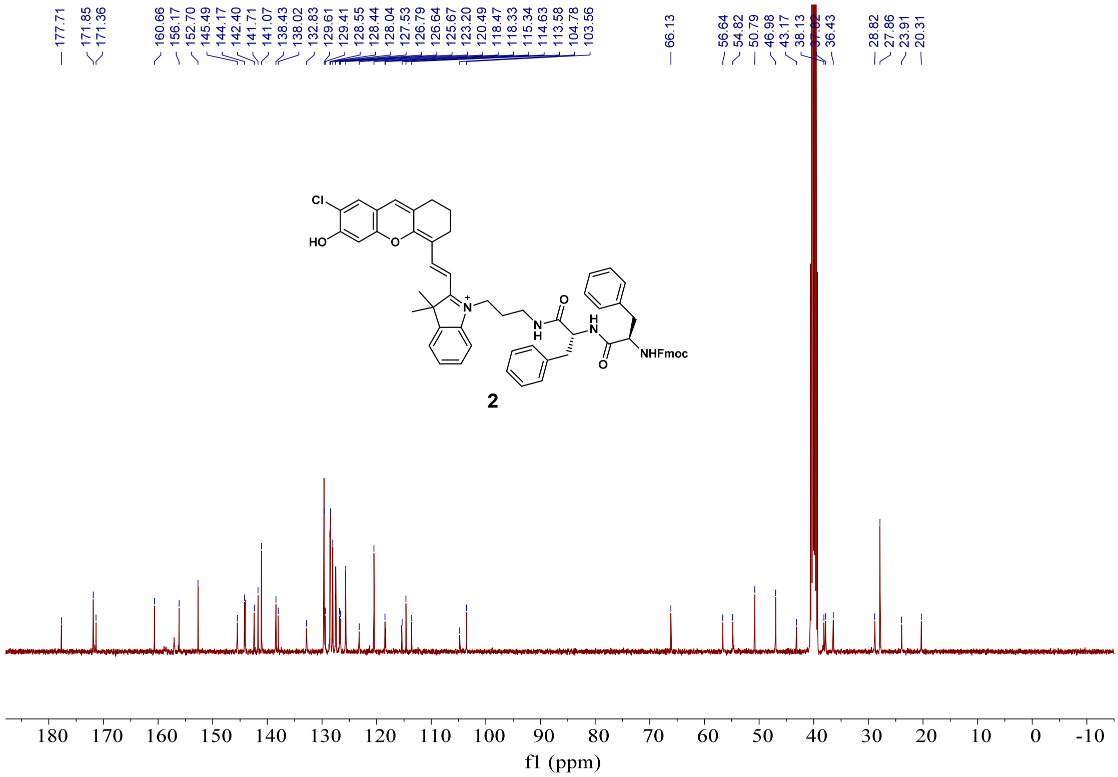

Supplementary Figure 44. ^{13}C-NMR spectra of compound **2** (101 MHz, DMSO-d_6).

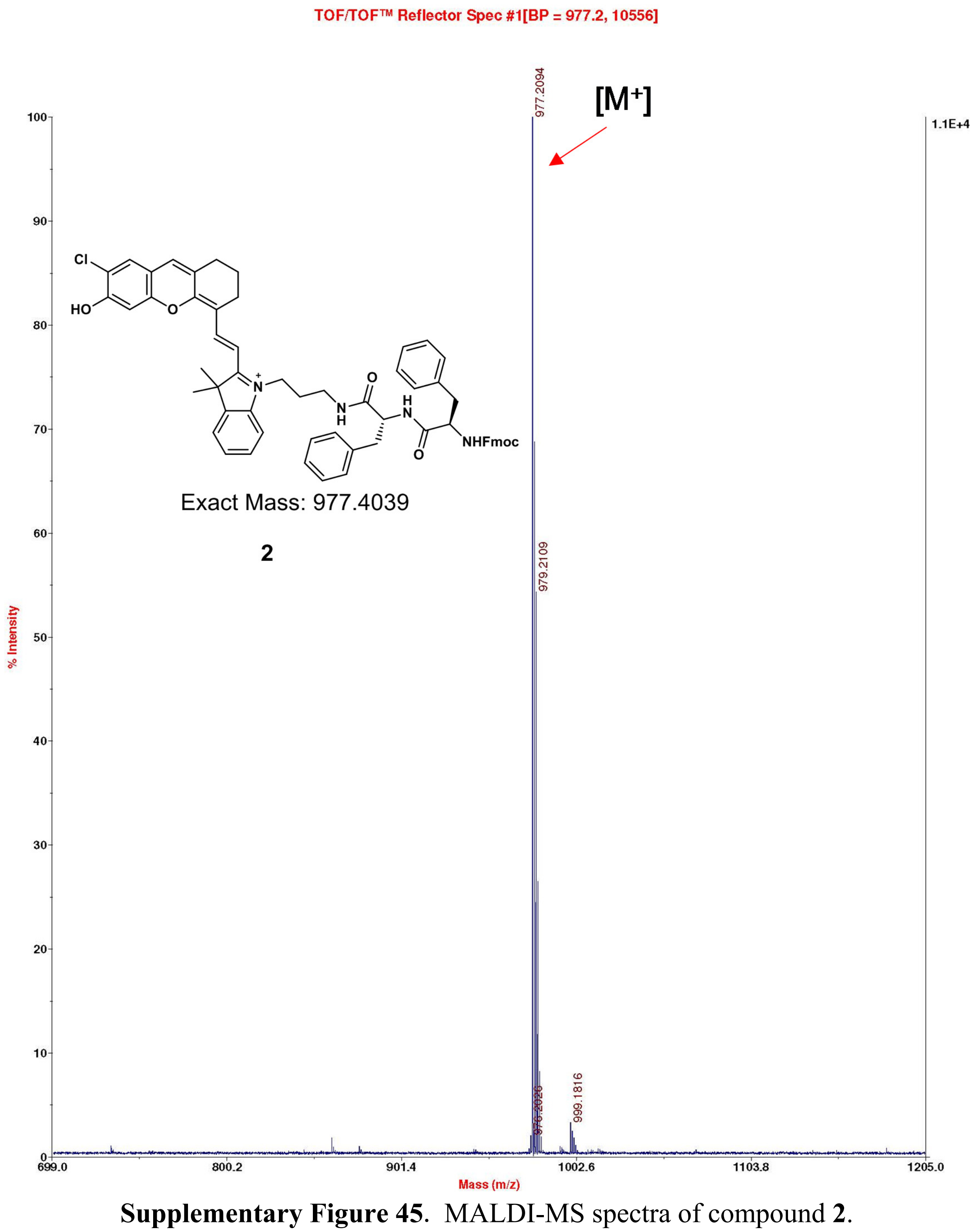

Supplementary Figure 45. MALDI-MS spectra of compound **2**.

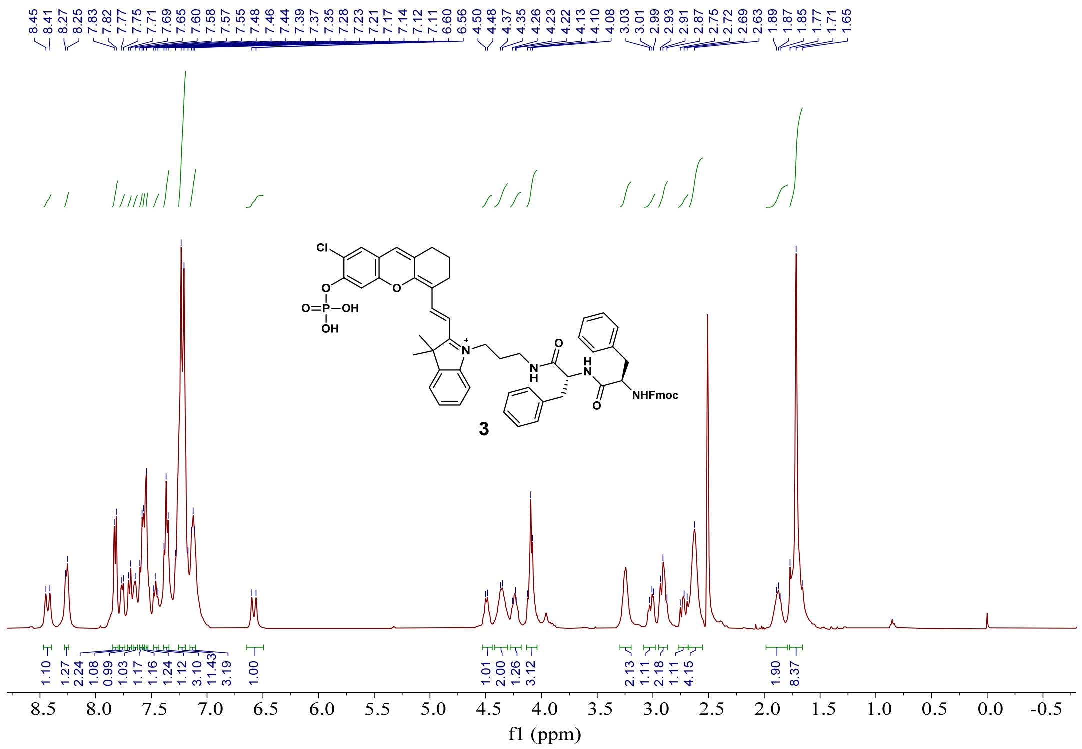

Supplementary Figure 46. ^{1}H-NMR spectra of compound **3** (400 MHz, DMSO-d_6).

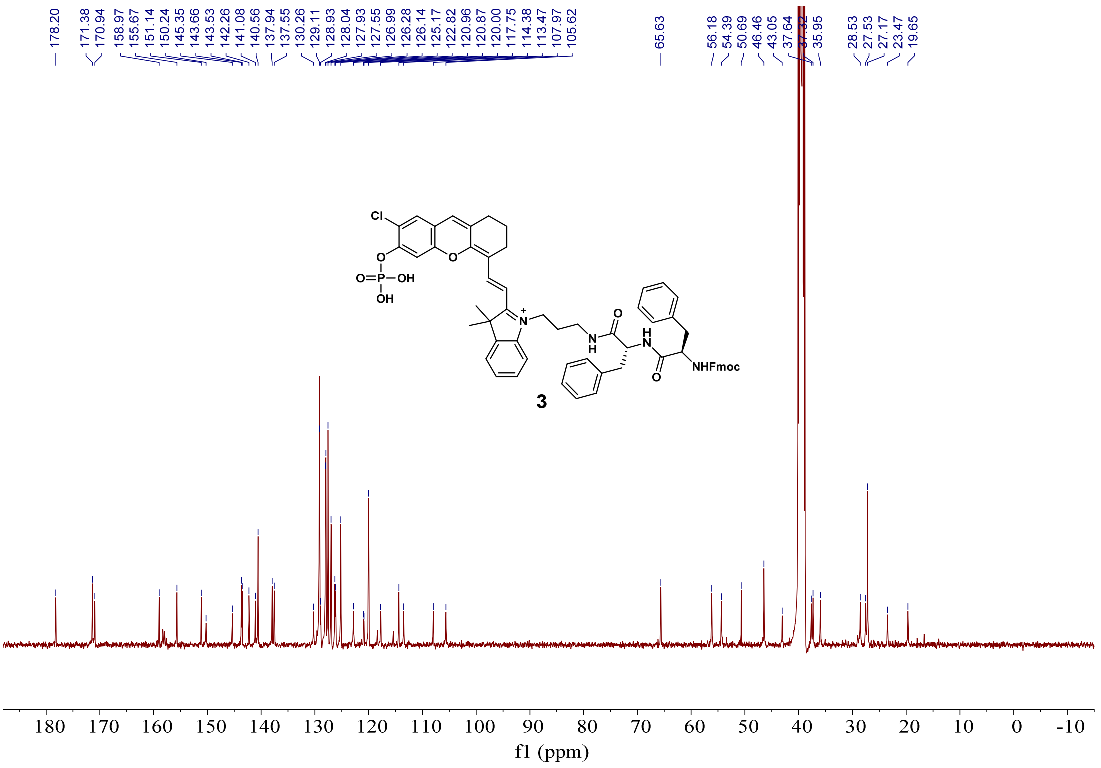

Supplementary Figure 47. ^{13}C-NMR spectra of compound **3** (101 MHz, DMSO-d_6).

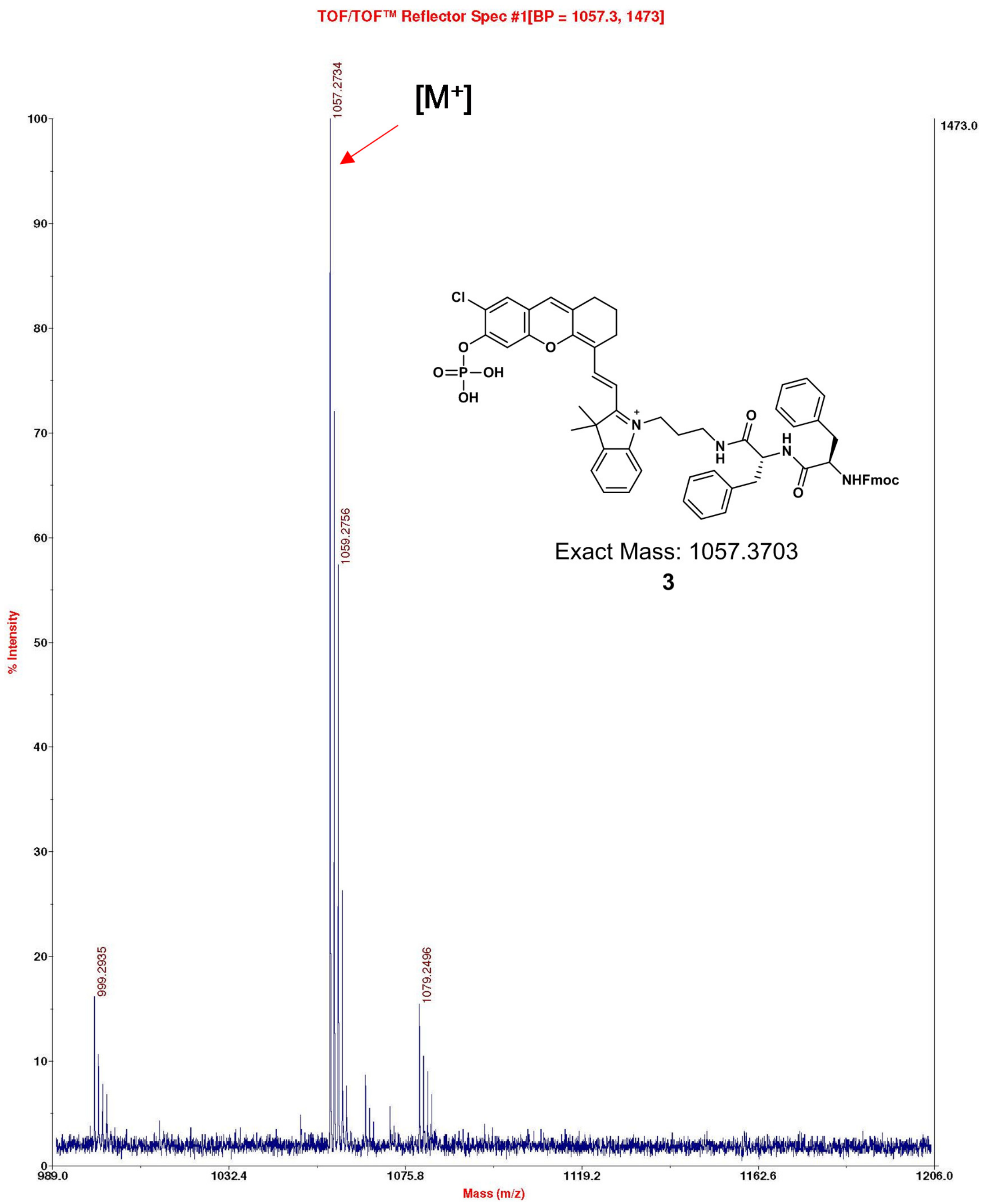

Supplementary Figure 48. MALDI-MS spectra of compound **3**.

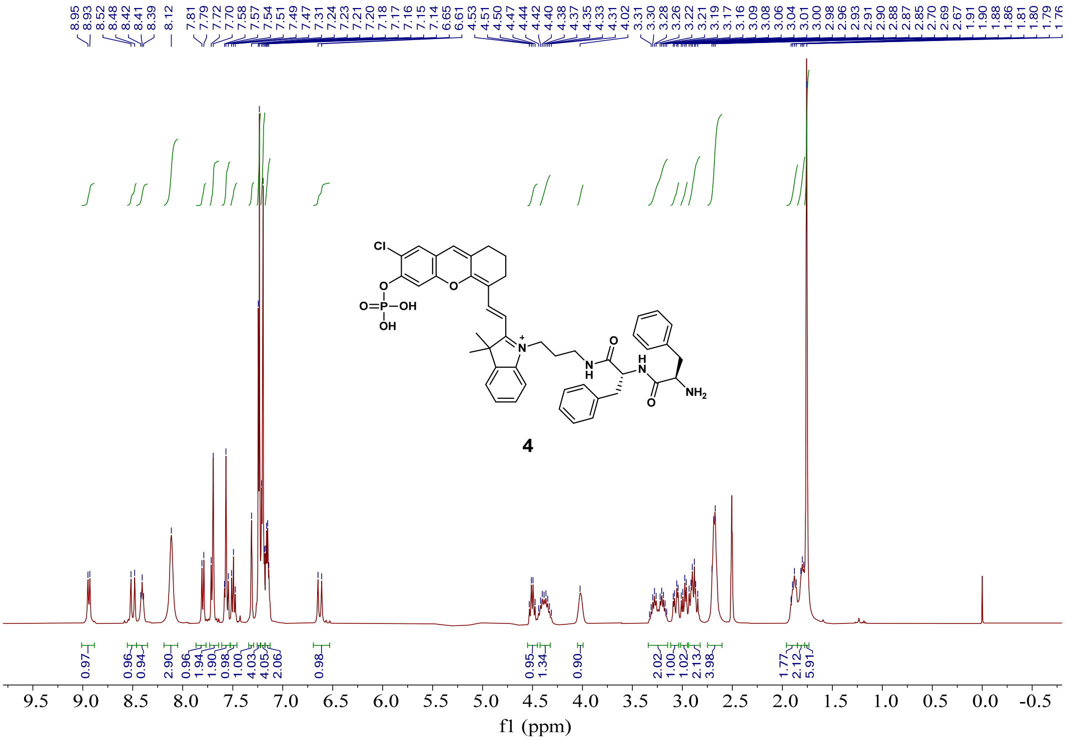

Supplementary Figure 49. ^{1}H-NMR spectra of compound **4** (400 MHz, DMSO-d_6).

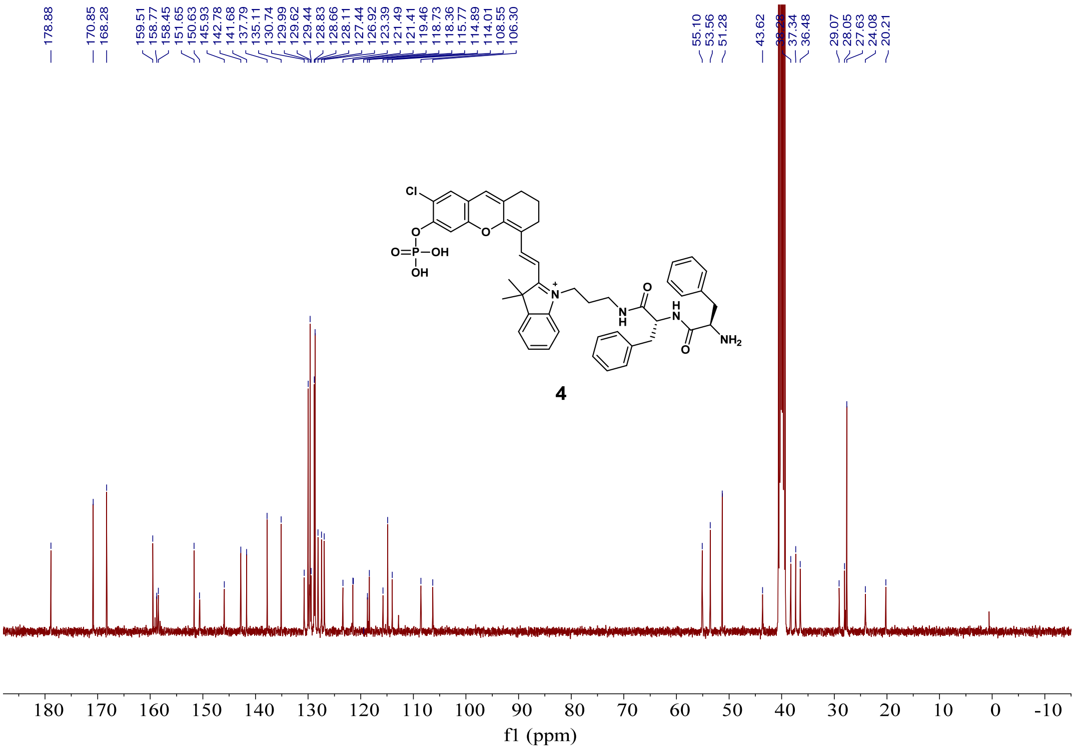

Supplementary Figure 50. ^{13}C-NMR spectra of compound **4** (101 MHz, DMSO-d_6).

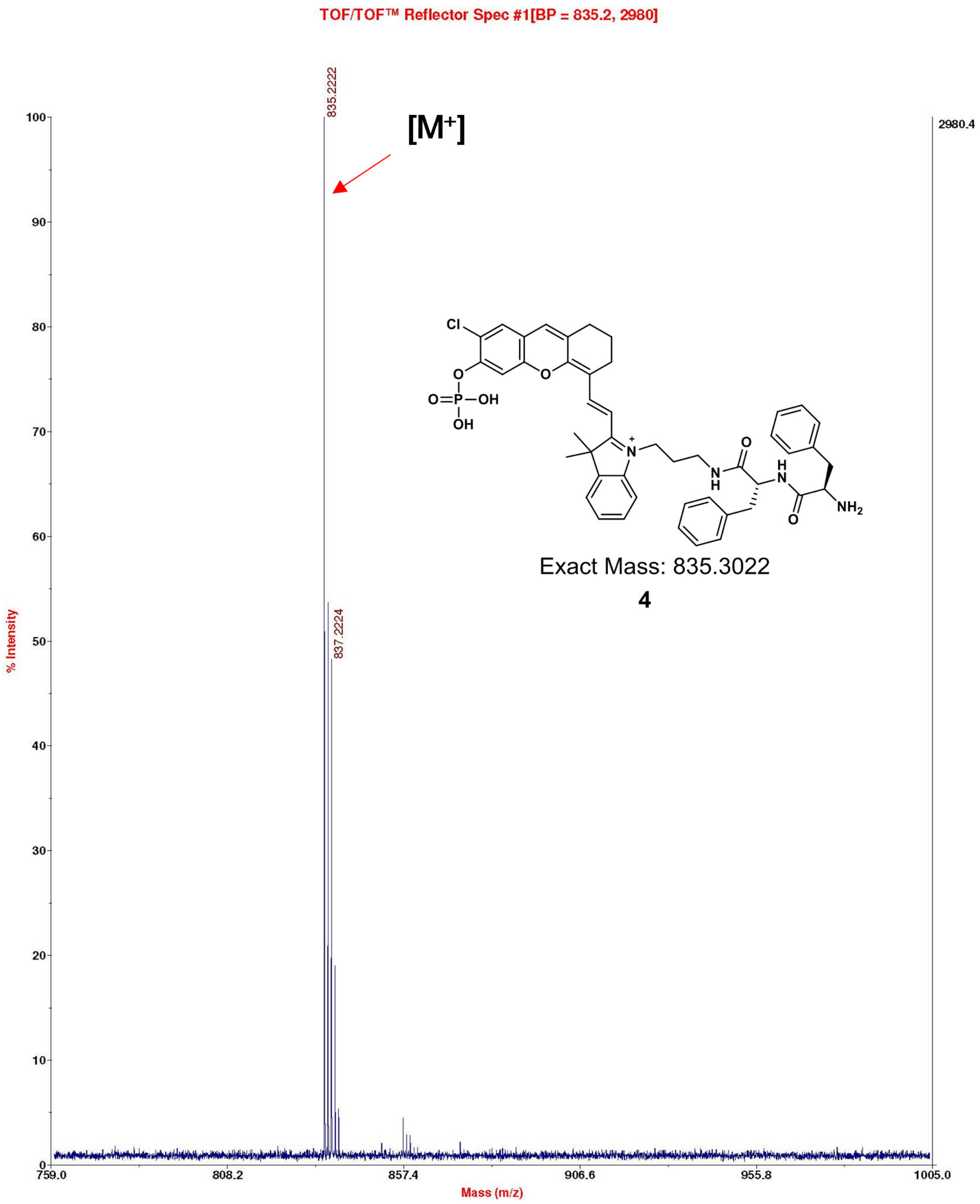

Supplementary Figure 51. MALDI-MS spectra of compound **4**.

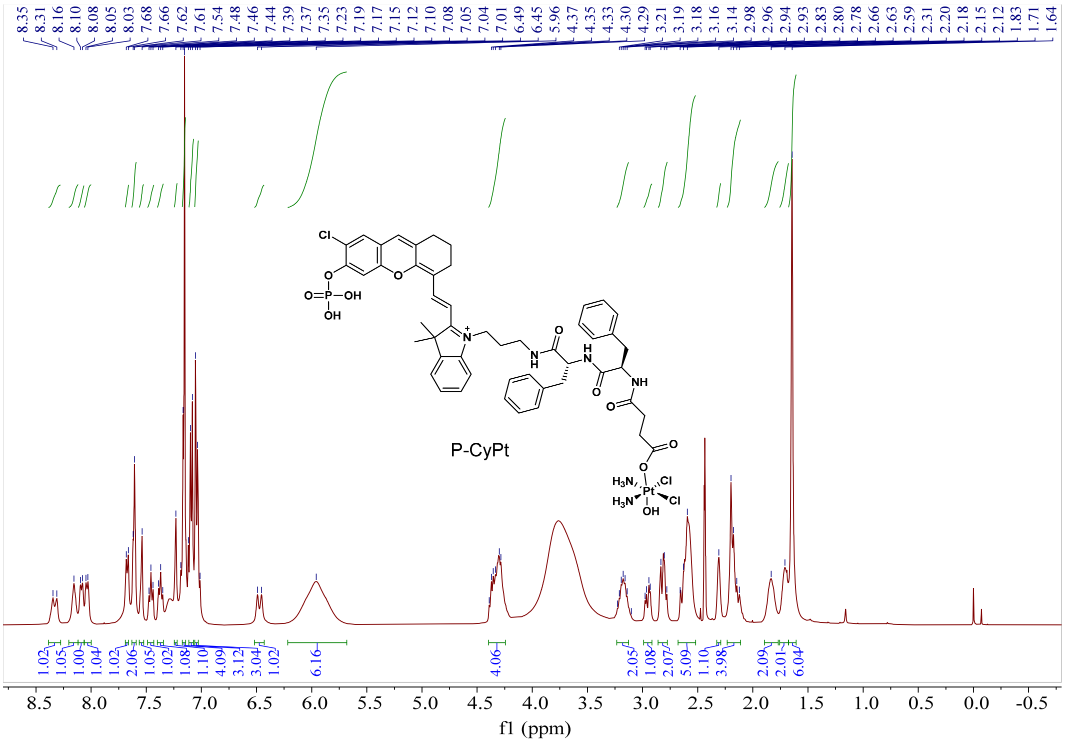

Supplementary Figure 52. ^{1}H-NMR spectra of P-CyPt (400 MHz, DMSO-d_6).

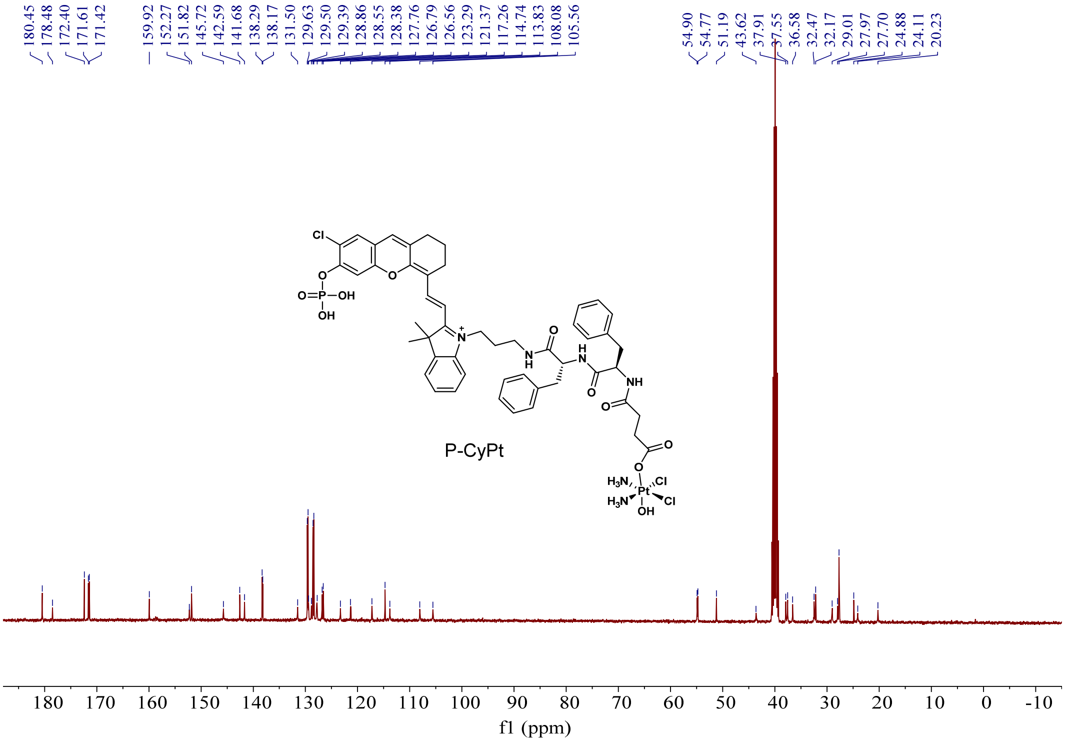

Supplementary Figure 53. ^{13}C-NMR spectra of P-CyPt (101 MHz, DMSO-d_6).

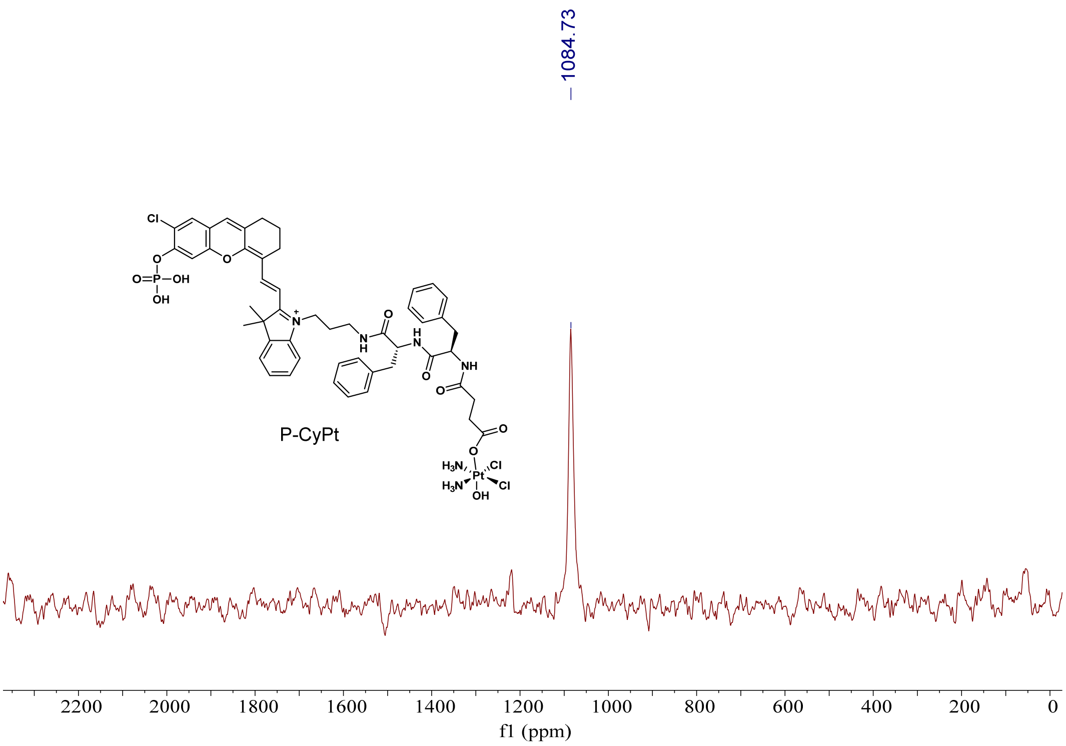

Supplementary Figure 54. ^{195}Pt-NMR spectra of P-CyPt (86 MHz, DMSO-*d*6).

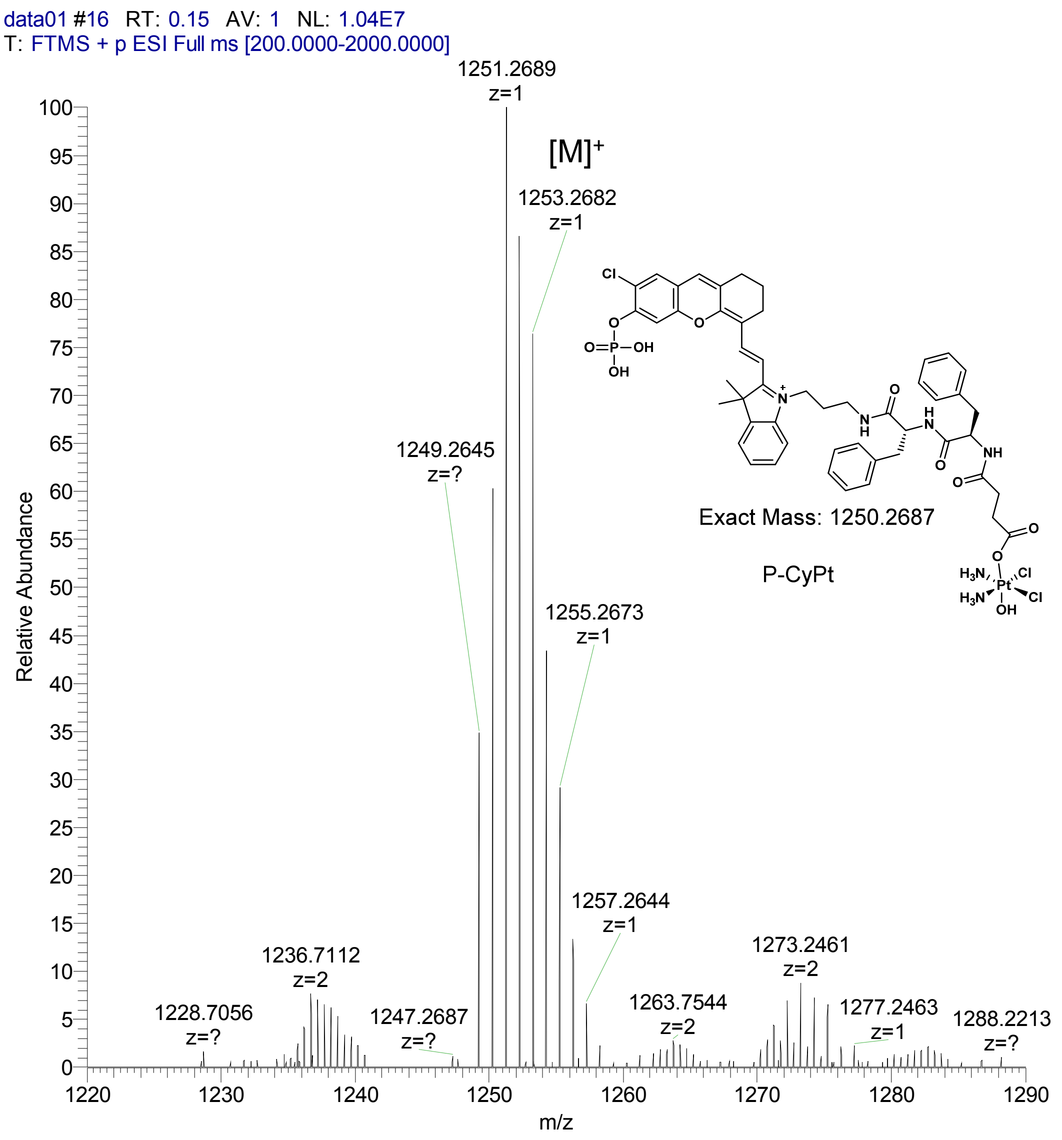

Supplementary Figure 55. High resolution ESI-MS spectra of P-CyPt.

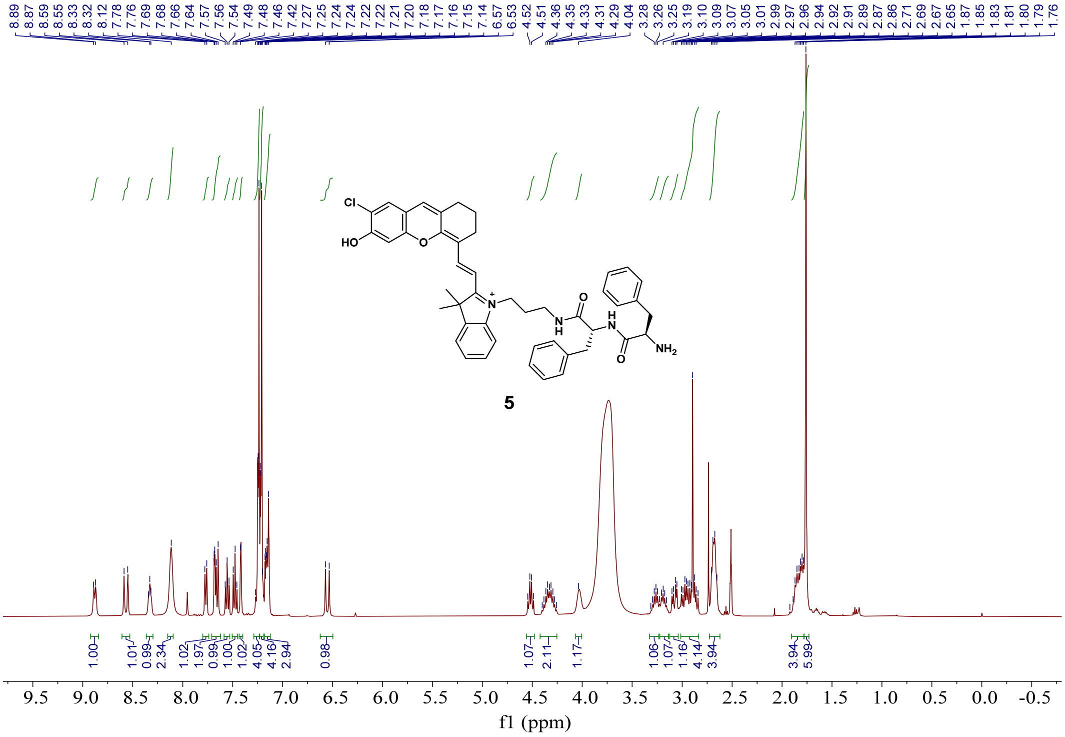

Supplementary Figure 56. ^{1}H-NMR spectra of compound **5** (400 MHz, DMSO-d_6).

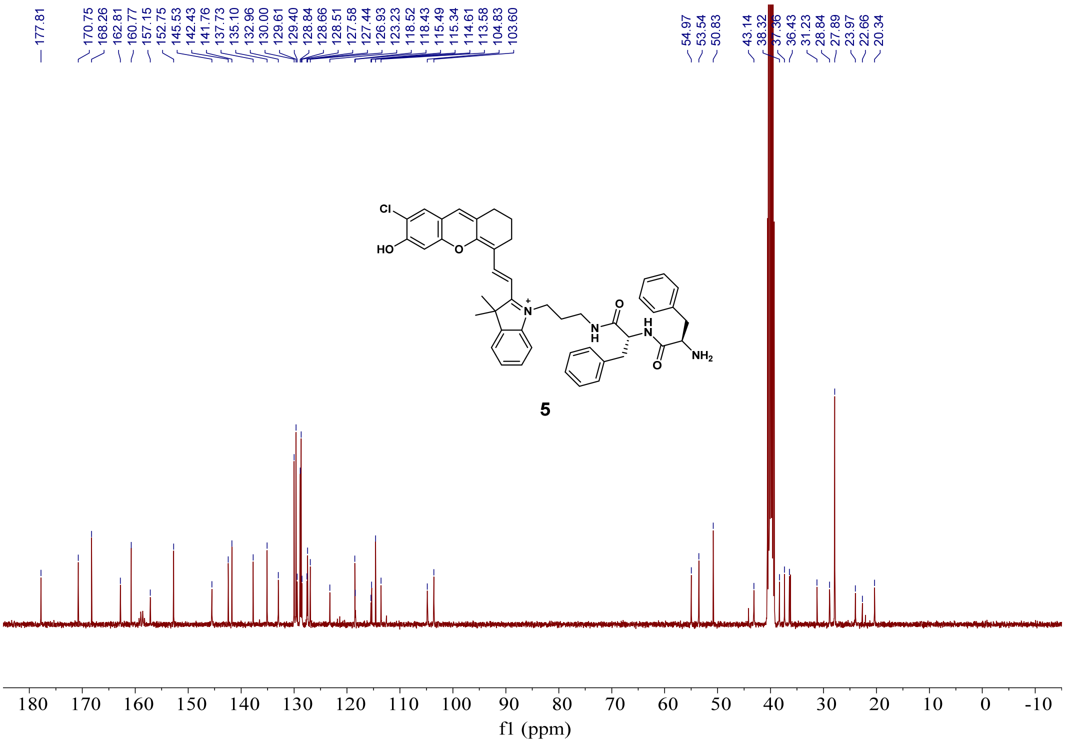

Supplementary Figure 57. ^{13}C-NMR spectra of compound **5** (101 MHz, DMSO-d_6).

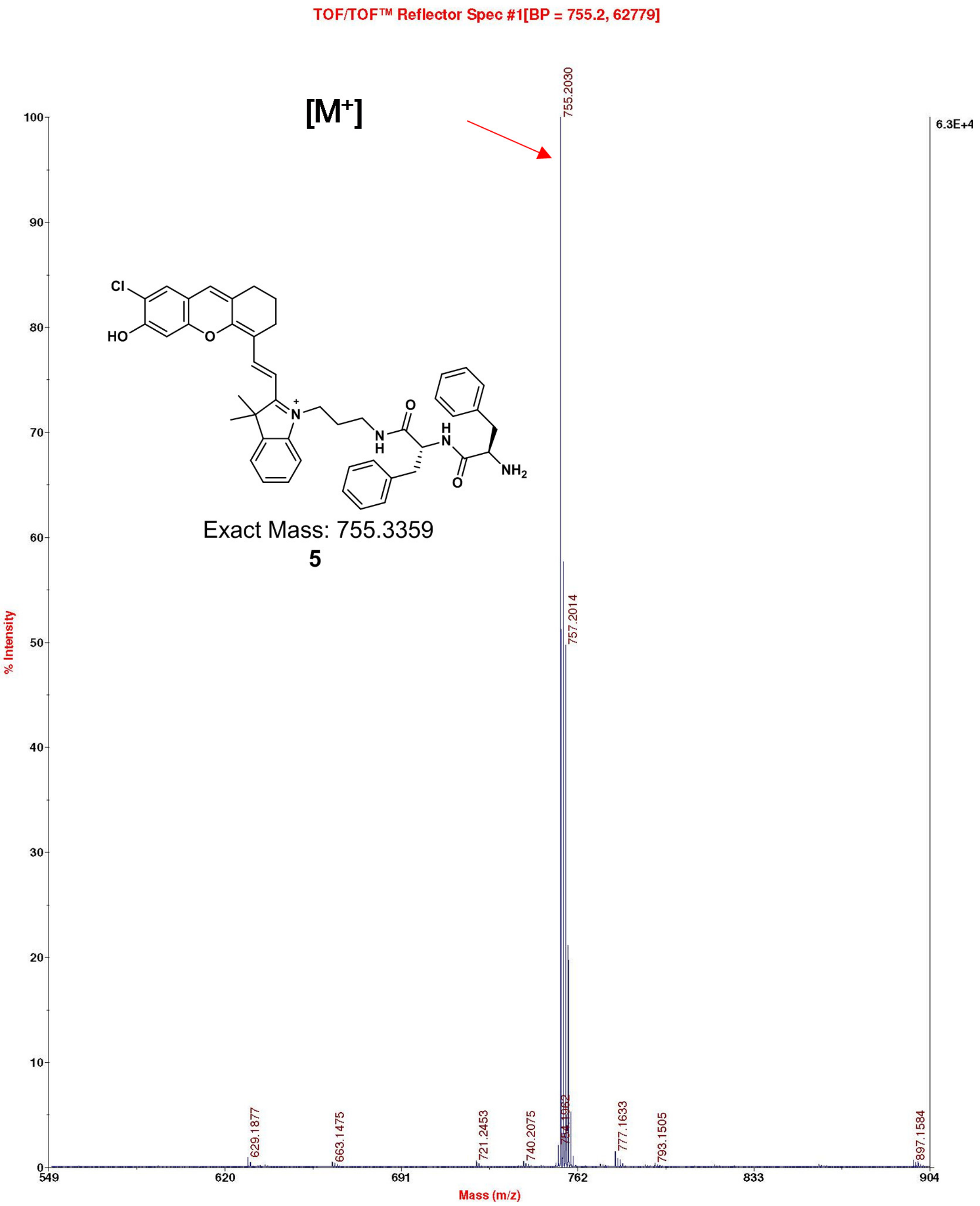

Supplementary Figure 58. MALDI-MS spectra of compound **5**

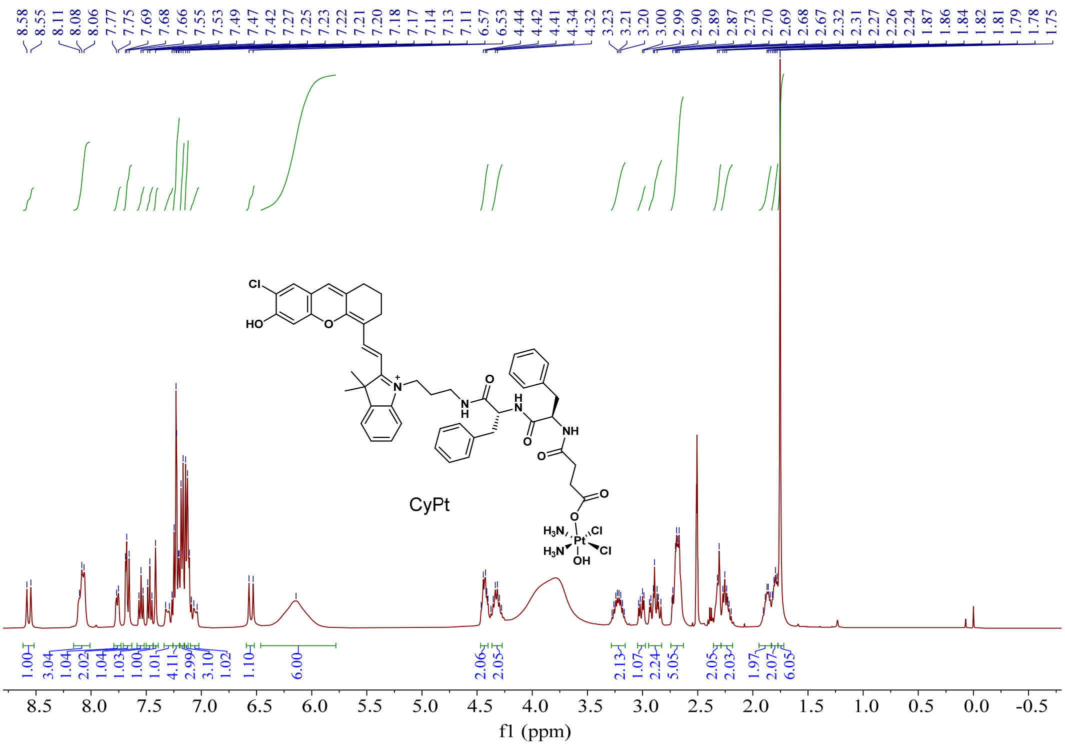

Supplementary Figure 59. ^{1}H-NMR spectra of CyPt (400 MHz, DMSO-d_6).

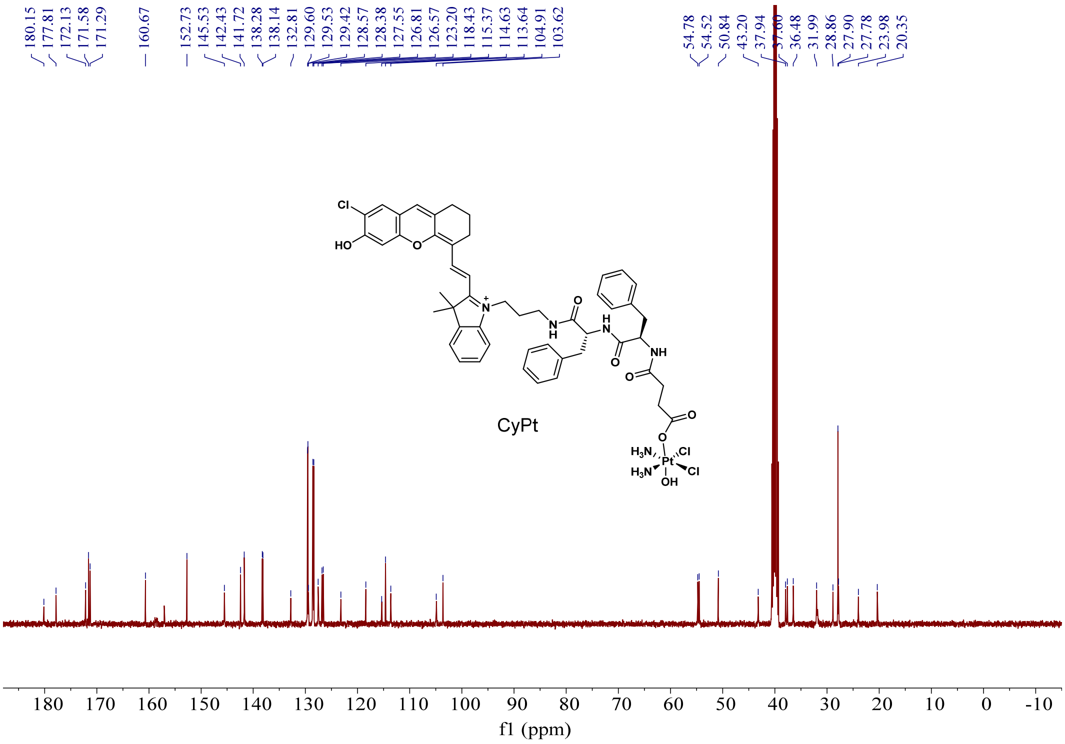

Supplementary Figure 60. ^{13}C-NMR spectra of CyPt (101 MHz, DMSO-d_6).

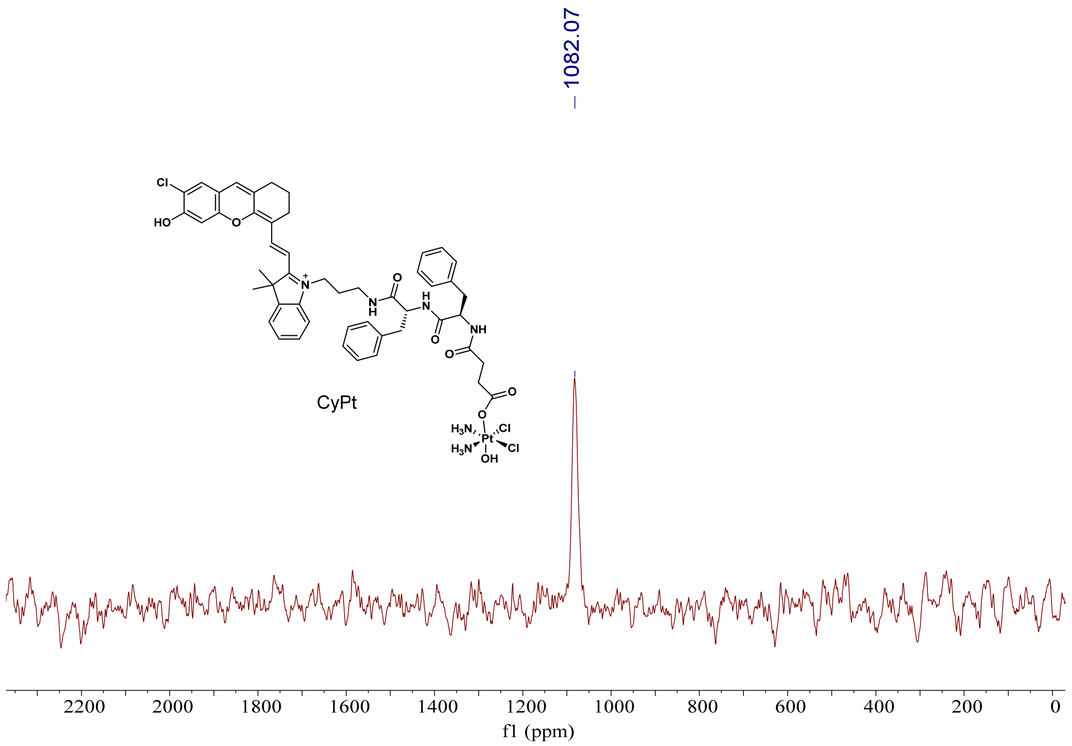

Supplementary Figure 61. ^{195}Pt-NMR spectra of CyPt (86 MHz, DMSO-d_6).

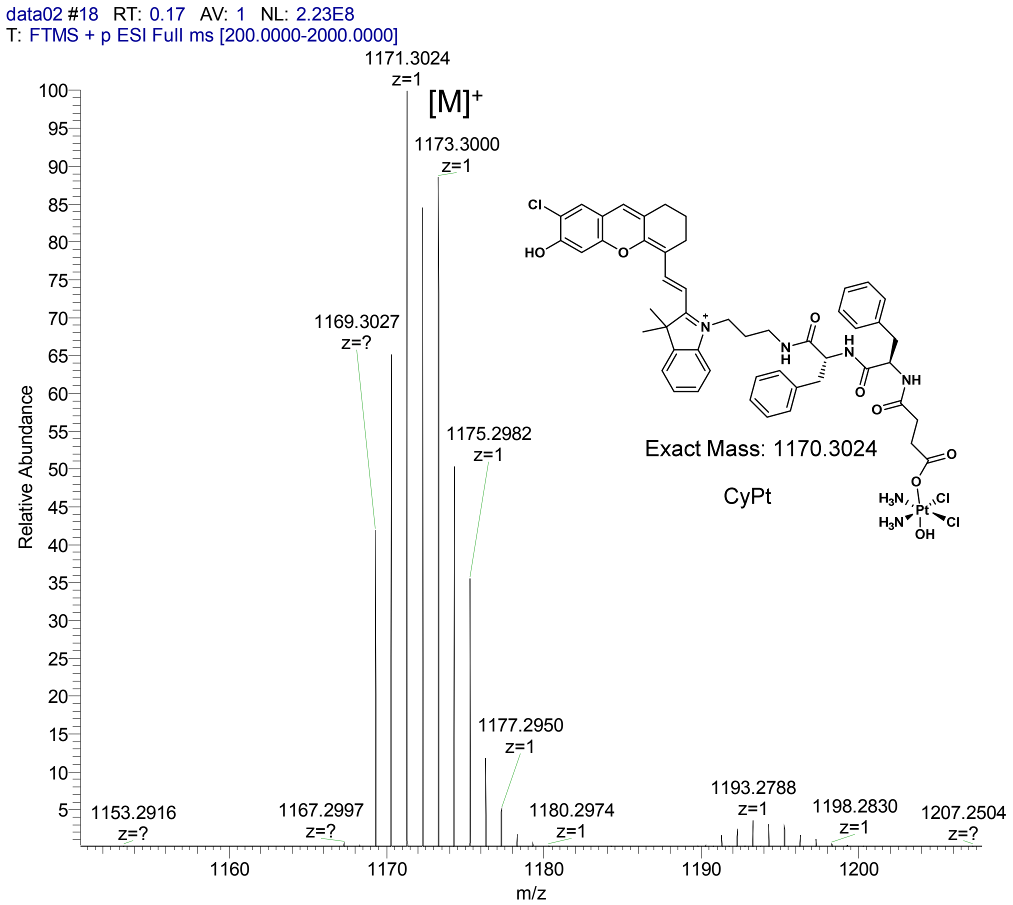

Supplementary Figure 62. High resolution ESI-MS spectrum of CyPt.

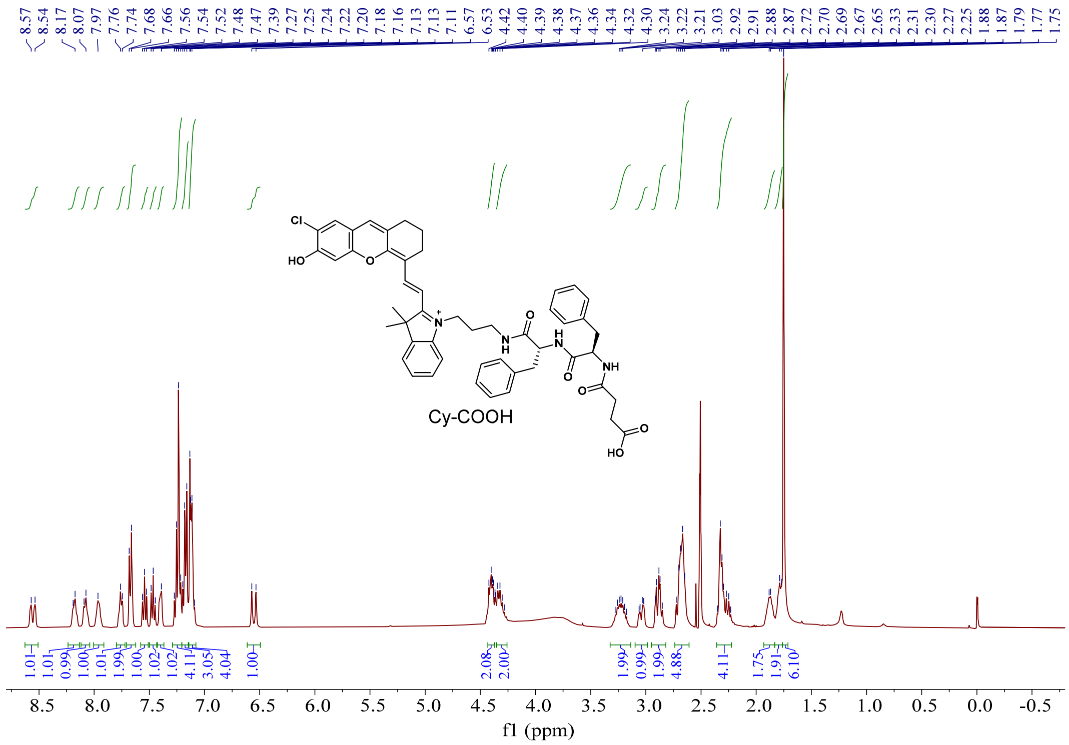

Supplementary Figure 63. ^{1}H-NMRspectra of Cy-COOH (400 MHz, DMSO-d_6).

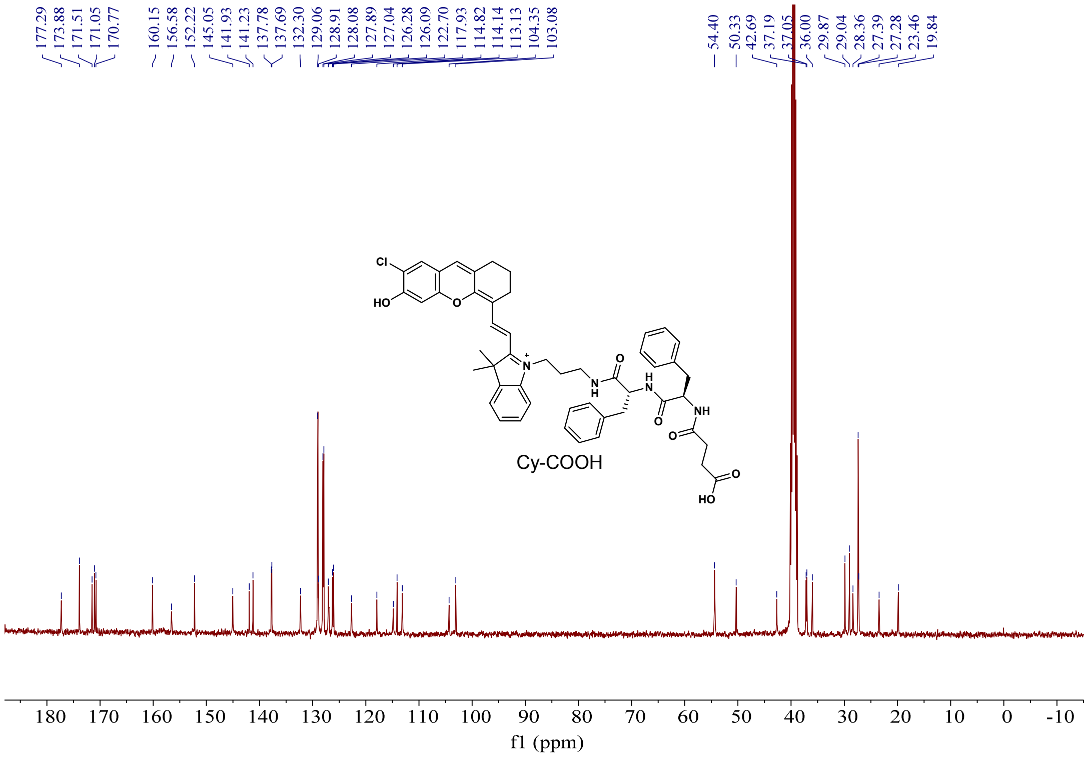

Supplementary Figure 64. ^{13}C-NMR spectra of Cy-COOH (101 MHz, DMSO-d_6).

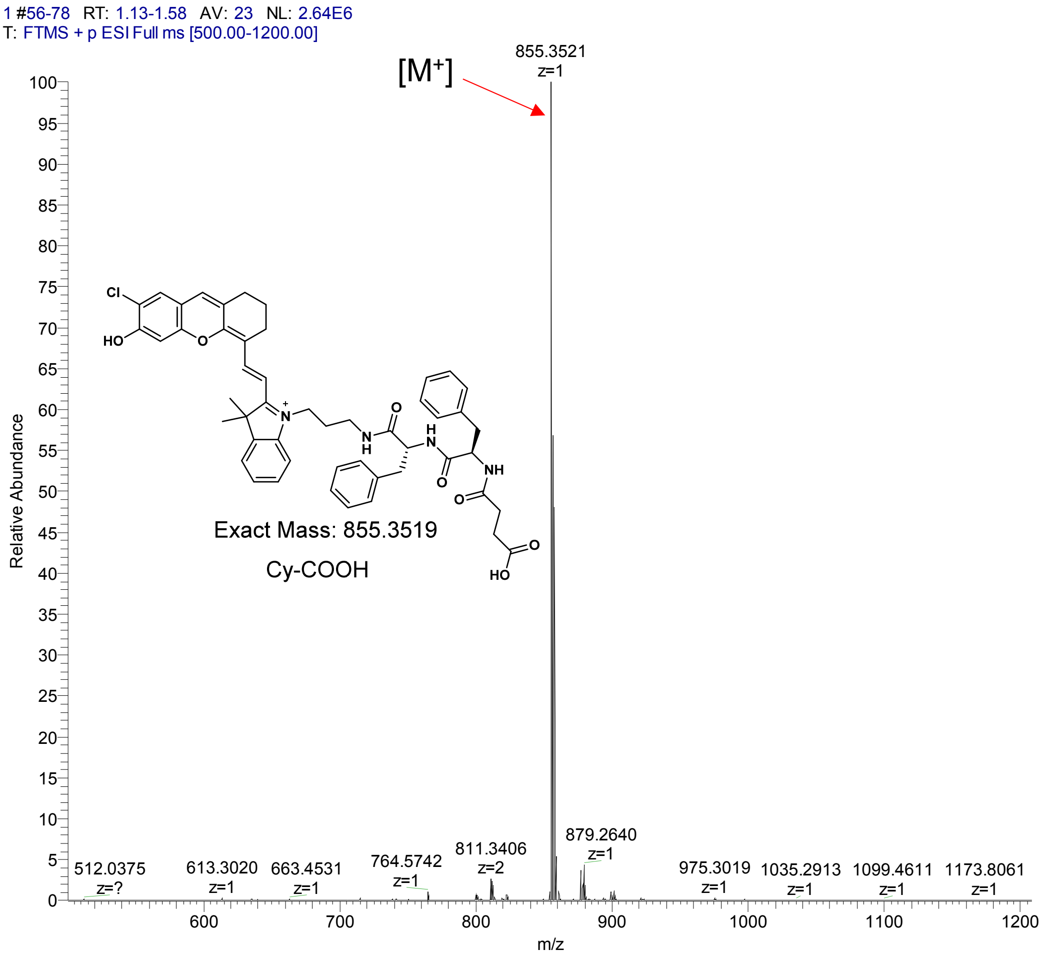

Supplementary Figure 65. High resolution ESI-MS spectrum of Cy-COOH.
