# Supplementary material for: Controlled sequential in situ self-assembly and disassembly of a fluorogenic cisplatin prodrug for cancer theranostics
Source: Nat Commun. 2023 Feb 13;14:800. doi: 10.1038/s41467-023-36469-1 (PMC9925730; doi:10.1038/s41467-023-36469-1)
Supplement: Supplementary file 2 — Reporting Summary [file 41467_2023_36469_MOESM2_ESM.pdf]

nature portfolio

Corresponding author(s): Hong Liu*, Zijian Guo,* and Deju Ye*

Last updated by author(s): Jan 31, 2023

Reporting Summary

Nature Portfolio wishes to improve the reproducibility of the work that we publish. This form provides structure for consistency and transparency in reporting. For further information on Nature Portfolio policies, see our Editorial Policies and the Editorial Policy Checklist.

Statistics

For all statistical analyses, confirm that the following items are present in the figure legend, table legend, main text, or Methods section.

n/a	Confirmed	
☐	☒	The exact sample size (n) for each experimental group/condition, given as a discrete number and unit of measurement
☐	☒	A statement on whether measurements were taken from distinct samples or whether the same sample was measured repeatedly
☐	☒	The statistical test(s) used AND whether they are one- or two-sided<br>*Only common tests should be described solely by name; describe more complex techniques in the Methods section.*
☐	☒	A description of all covariates tested
☐	☒	A description of any assumptions or corrections, such as tests of normality and adjustment for multiple comparisons
☐	☒	A full description of the statistical parameters including central tendency (e.g. means) or other basic estimates (e.g. regression coefficient) AND variation (e.g. standard deviation) or associated estimates of uncertainty (e.g. confidence intervals)
☐	☒	For null hypothesis testing, the test statistic (e.g. F, t, r) with confidence intervals, effect sizes, degrees of freedom and P value noted<br>*Give P values as exact values whenever suitable.*
☒	☐	For Bayesian analysis, information on the choice of priors and Markov chain Monte Carlo settings
☒	☐	For hierarchical and complex designs, identification of the appropriate level for tests and full reporting of outcomes
☐	☒	Estimates of effect sizes (e.g. Cohen's d, Pearson's r), indicating how they were calculated

Our web collection on statistics for biologists contains articles on many of the points above.

Software and code

Policy information about availability of computer code

Data collection	No software was used.
Data analysis	All software used for data analysis are fully described in the materials and methods of the manuscript. Fluorescence and bioluminescece images were analyzed using the Living Image Software (4.5.2, PerkinElmer, MA, U.S.A). Photoacoustic (PA) images were acquired with Vevo 2100 LAZR (FUJIFILM VisualSonics, Canada). Statistical calculations were performed using GraphPad Prism 6 (GraphPad Software Inc., CA, USA), The chemical draw were performed using ChemDraw professional 19.0.0.22(PerkinElmer, U.S.A). The data anlaysis were performed using OriginPro 2022b(64-bit) SR1 9.9.5171 (learing Edition). Flow cytometric analysis were performed using FlowJoX (10.0.7)

For manuscripts utilizing custom algorithms or software that are central to the research but not yet described in published literature, software must be made available to editors and reviewers. We strongly encourage code deposition in a community repository (e.g. GitHub). See the Nature Portfolio guidelines for submitting code & software for further information.

Data

Policy information about availability of data

All manuscripts must include a data availability statement. This statement should provide the following information, where applicable:

- Accession codes, unique identifiers, or web links for publicly available datasets
- A description of any restrictions on data availability
- For clinical datasets or third party data, please ensure that the statement adheres to our policy

The data that support the findings of this study are available within the main text and its Supplementary Information file. Source data is provided as Source Data file.

Data is also available from the corresponding author upon request.

Human research participants

Policy information about studies involving human research participants and Sex and Gender in Research.

Reporting on sex and gender	Irrelevant to experiments.
Population characteristics	Irrelevant to experiments.
Recruitment	Irrelevant to experiments.
Ethics oversight	Irrelevant to experiments.

Note that full information on the approval of the study protocol must also be provided in the manuscript.

Field-specific reporting

Please select the one below that is the best fit for your research. If you are not sure, read the appropriate sections before making your selection.

☒ Life sciences ☐ Behavioural & social sciences ☐ Ecological, evolutionary & environmental sciences

For a reference copy of the document with all sections, see nature.com/documents/nr-reporting-summary-flat.pdf

Life sciences study design

All studies must disclose on these points even when the disclosure is negative.

Sample size	No calculations were done to determine sample size. Sample sizes were determined according to precedent in previous literatures to enable statistical analyses such as standard deviation and t-tests. In vitro studies were repeated at least two or three times independently and in vivo sample sizes were determined based on standards for animal studies, attempting to have a minimum of N = 3 biological replicates with sufficient reproducibility.
Data exclusions	No data was excluded from this study.
Replication	Experiments were repeated at least two or three independent experiments with similar results. All experiments were reproduced to reliably support conclusions stated in the manuscript.
Randomization	For in vivo study, mice were inoculated with the same bath of tumor cells and randomly divided into experimental groups. For in vitro studies, all cells from different dishes were combined, counted and then plated into wells, then treated with various conditions. Thus, all treatment groups have the same cell stocks. Biological independent experiments were performed on independent aliquots of cells thawed from the liquid nitrogen freezer.
Blinding	Data acquisition and analyses were not blinded but all assays were performed at the same time for all groups of a given experiment. Since all conditions were subjected to the same analyses, blinding was not considered to be necessary.

Reporting for specific materials, systems and methods

We require information from authors about some types of materials, experimental systems and methods used in many studies. Here, indicate whether each material, system or method listed is relevant to your study. If you are not sure if a list item applies to your research, read the appropriate section before selecting a response.

Materials & experimental systems

n/a	Involved in the study	
☐	☒	Antibodies
☐	☒	Eukaryotic cell lines
☒	☐	Palaeontology and archaeology
☐	☒	Animals and other organisms
☒	☐	Clinical data
☒	☐	Dual use research of concern

Methods

n/a	Involved in the study	
☒	☐	ChIP-seq
☐	☒	Flow cytometry
☒	☐	MRI-based neuroimaging

Antibodies

Antibodies used	Mouse monoclonal ALP, Tissue Non-Specific antibody (Catalog # ab126820) , Anti-gamma H2A.X (phospho S139) rabbit monoclonal antibody (Cata# ab81299), and Alexa Fluor®488 goat anti-rabbit IgG(H+L), (Catalog # ab150077) were purchased from Abcam (Cambridge,UK).
Validation	Validation details of the primary antibodies are available on the manufacturers' websites:<br>https://www.abcam.cn/alkaline-phosphatase-tissue-non-specific-antibody-2f4-ab126820.html<br>https://www.abcam.cn/gamma-h2ax-phospho-s139-antibody-ep8542y-ab81299.html<br>https://www.abcam.cn/goat-rabbit-igg-hl-alexa-fluor-488-ab150077.html

Eukaryotic cell lines

Policy information about cell lines and Sex and Gender in Research

Cell line source(s)	Human cervical cancer HeLa cells, Human hepatic cancer HepG2 cells and human embryonic kidney HEK293T cells were purchased from from Stem Cell Bank, Chinese Academy of Sciences (Shanghai, China).
Authentication	All cell lines were authenticated by the supplier using Short Tandem Repeat test.
Mycoplasma contamination	All cell lines tested were negative for mycoplasma contamination.
Commonly misidentified lines (See ICLAC register)	No commonly misidentified cell lines were used.

Animals and other research organisms

Policy information about studies involving animals; ARRIVE guidelines recommended for reporting animal research, and Sex and Gender in Research

Laboratory animals	Animal experiments were carried out under the guidelines of the Institutional Animal Care and Use Committee (IACUC) of Nanjing University. BALB/c female nude mice (~5-6 weeks old and ~6-8 weeks old) were purchased from the Model Animal Research Center (MARC) of Nanjing University. Mice were grouped and housed under a 12h light-dark cycle, 50–70% humidity, and at 18–22 °C ambient temperature, with free access to water and food.
Wild animals	The study did not involve wild animals.
Reporting on sex	Female nude mice and Female mice.
Field-collected samples	No field collected samples were used in the study.
Ethics oversight	The Institutional Animal Care and Use Committee (IACUC) of Nanjing University approved and provided guidance on the study protocol (Approval No: IACUC-2107004).

Note that full information on the approval of the study protocol must also be provided in the manuscript.

Flow Cytometry

Plots

Confirm that:

- [x] The axis labels state the marker and fluorochrome used (e.g. CD4-FITC).
- [x] The axis scales are clearly visible. Include numbers along axes only for bottom left plot of group (a 'group' is an analysis of identical markers).
- [x] All plots are contour plots with outliers or pseudocolor plots.
- [x] A numerical value for number of cells or percentage (with statistics) is provided.

Methodology

Sample preparation	All assays were performed according to the manufacturer's instructions. The Annexin V-FITC/PI double staining apoptosis detection kit (Catalog # KGA108) were purchased from keygentec (Nanjing,China).<br>Validation details of the primary Annexin V-FITC/PI reagent kits are available on the manufacturers' websites:<br>http://www.keygentec.com.cn/pro_detail.php?cid=102874&bid=446
Instrument	Coulter FC-500 flow cytometer
Software	FlowJoX 10.0.7.

Cell population abundance	The single sample the count number for each flow cytometry analysis was ~5000 to 10000
Gating strategy	All samples were gated equally.

☒ Tick this box to confirm that a figure exemplifying the gating strategy is provided in the Supplementary Information.
